# Posicionamento Brasileiro sobre Síndrome da Quilomicronemia Familiar – 2023

**DOI:** 10.36660/abc.20230203

**Published:** 2023-03-23

**Authors:** Maria Cristina de Oliveira Izar, Raul Dias dos Santos, Marcelo Heitor Vieira Assad, Antonio Carlos Palandri Chagas, Alceu de Oliveira Toledo, Ana Cláudia Cavalcante Nogueira, Ana Cristina Carneiro Fernandes Souto, Ana Maria Lottenberg, Ana Paula Marte Chacra, Carlos Eduardo dos Santos Ferreira, Charles Marques Lourenço, Cynthia Melissa Valerio, Dennys Esper Cintra, Francisco Antonio Helfenstein Fonseca, Gustavo Aguiar Campana, Henrique Tria Bianco, Josivan Gomes de Lima, Maria Helane Costa Gurgel Castelo, Marileia Scartezini, Miguel Antonio Moretti, Natasha Slhessarenko Fraife Barreto, Rayana Elias Maia, Renan Magalhães Montenegro, Renato Jorge Alves, Roberta Marcondes Machado Figueiredo, Rodrigo Ambrosio Fock, Tânia Leme da Rocha Martinez, Viviane Zorzanelli Rocha Giraldez

**Affiliations:** 1 Universidade Federal de São Paulo São Paulo SP Brasil Universidade Federal de São Paulo (UNIFESP), São Paulo, SP – Brasil; 2 Universidade de São Paulo São Paulo SP Brasil Universidade de São Paulo (USP), São Paulo, SP – Brasil; 3 Instituto Nacional de cardiologia Rio de Janeiro RJ Brasil Instituto Nacional de cardiologia (INC), Rio de Janeiro, RJ – Brasil; 4 Faculdade de Medicina do ABC São Caetano do Sul SP Brasil Faculdade de Medicina do ABC, São Caetano do Sul, SP – Brasil; 5 Universidade Estadual de Ponta Grossa Ponta Grossa PR Brasil Universidade Estadual de Ponta Grossa (UEPG), Ponta Grossa, PR – Brasil; 6 Hospital de Base do Distrito Federal Brasília DF Brasil Hospital de Base do Distrito Federal, Brasília, DF – Brasil; 7 Hospital das Clínicas Faculdade de Medicina Universidade de São Paulo São Paulo SP Brasil Instituto do Coração (Incor) do Hospital das Clínicas da Faculdade de Medicina da Universidade de São Paulo (HCFMUSP), São Paulo, SP – Brasil; 8 Hospital das Clínicas Faculdade de Medicina Universidade de São Paulo São Paulo SP Brasil Laboratório de Lípides (LIM 10) do Hospital das Clínicas da Faculdade de Medicina da Universidade de São Paulo (HCFMUSP), São Paulo, SP – Brasil; 9 Hospital Israelita Albert Einstein São Paulo SP Brasil Hospital Israelita Albert Einstein (HIAE), São Paulo, SP – Brasil; 10 Faculdade de Medicina de São José do Rio Preto São José do Rio Preto SP Brasil Faculdade de Medicina de São José do Rio Preto (FAMERP), São José do Rio Preto, SP – Brasil; 11 Instituto Estadual de Diabetes e Endocrinologia Luiz Capriglione Rio de Janeiro RJ Brasil Instituto Estadual de Diabetes e Endocrinologia Luiz Capriglione (IEDE-RJ), Rio de Janeiro, RJ – Brasil; 12 Universidade Estadual de Campinas Campinas SP Brasil Universidade Estadual de Campinas (UNICAMP), Campinas, SP – Brasil; 13 Alliar Medicina Diagnóstica São Paulo SP Brasil Alliar Medicina Diagnóstica, São Paulo, SP – Brasil; 14 Hospital Universitário Onofre Lopes Universidade Federal do Rio Grande do Norte Natal RN Brasil Hospital Universitário Onofre Lopes da Universidade Federal do Rio Grande do Norte (UFRN), Natal, RN – Brasil; 15 Diagnósticos da América S.A. Fortaleza CE Brasil Diagnósticos da América S.A. (DASA), Fortaleza, CE – Brasil; 16 Universidade Federal do Paraná Curitiba PR Brasil Universidade Federal do Paraná (UFPR), Curitiba, PR – Brasil; 17 Universidade Federal de Mato Grosso Cuiabá MT Brasil Universidade Federal de Mato Grosso (UFMT), Cuiabá, MT – Brasil; 18 Universidade Federal da Paraíba João Pessoa PB Brasil Universidade Federal da Paraíba (UFPB), João Pessoa, PB – Brasil; 19 Universidade Federal do Ceará Empresa Brasileira de Serviços Hospitalares Fortaleza CE Brasil Complexo Hospitalar da Universidade Federal do Ceará (UFCE), Empresa Brasileira de Serviços Hospitalares (EBSERH), Fortaleza, CE – Brasil; 20 Hospital Santa Casa de Misericórdia de São Paulo São Paulo SP Brasil Hospital Santa Casa de Misericórdia de São Paulo, São Paulo, SP – Brasil; 21 Faculdade Israelita de Ciências da Saúde Albert Einstein São Paulo SP Brasil Faculdade Israelita de Ciências da Saúde Albert Einstein (FICSAE), São Paulo, SP – Brasil; 22 Hospital Beneficência Portuguesa de São Paulo São Paulo SP Brasil Hospital Beneficência Portuguesa de São Paulo, São Paulo, SP – Brasil

**Table:** 

**Posicionamento Brasileiro sobre Síndrome da Quilomicronemia Familiar – 2023**	
**O relatório abaixo lista as declarações de interesse conforme relatadas à SBC pelos especialistas durante o período de desenvolvimento deste posicionamento, 2022.**	
**Especialista**	**Tipo de relacionamento com a indústria**
Alceu de Oliveira Toledo Júnior	Nada a ser declarado
Ana Cláudia Cavalcante Nogueira	Declaração financeira A - Pagamento de qualquer espécie e desde que economicamente apreciáveis, feitos a (i) você, (ii) ao seu cônjuge/ companheiro ou a qualquer outro membro que resida com você, (iii) a qualquer pessoa jurídica em que qualquer destes seja controlador, sócio, acionista ou participante, de forma direta ou indireta, recebimento por palestras, aulas, atuação como proctor de treinamentos, remunerações, honorários pagos por participações em conselhos consultivos, de investigadores, ou outros comitês, etc. Provenientes da indústria farmacêutica, de órteses, próteses, equipamentos e implantes, brasileiras ou estrangeiras: - Servier: Vastarel/Angina. Outros relacionamentos Financiamento de atividades de educação médica continuada, incluindo viagens, hospedagens e inscrições para congressos e cursos, provenientes da indústria farmacêutica, de órteses, próteses, equipamentos e implantes, brasileiras ou estrangeiras: - PTC: Waylivra/SQF. Participação societária de qualquer natureza e qualquer valor economicamente apreciável de empresas na área de saúde, de ensino ou em empresas concorrentes ou fornecedoras da SBC: - Área de ensino e pesquisa clínica.
Ana Cristina Carneiro Fernandes Souto	Nada a ser declarado
Ana Maria Pitta Lottenberg	Declaração financeira A - Pagamento de qualquer espécie e desde que economicamente apreciáveis, feitos a (i) você, (ii) ao seu cônjuge/ companheiro ou a qualquer outro membro que resida com você, (iii) a qualquer pessoa jurídica em que qualquer destes seja controlador, sócio, acionista ou participante, de forma direta ou indireta, recebimento por palestras, aulas, atuação como proctor de treinamentos, remunerações, honorários pagos por participações em conselhos consultivos, de investigadores, ou outros comitês, etc. Provenientes da indústria farmacêutica, de órteses, próteses, equipamentos e implantes, brasileiras ou estrangeiras: - PTC Therapeutics: Apoio Financeiro para palestras.
Ana Paula Marte Chacra	Nada a ser declarado
Antonio Carlos Palandri Chagas	Declaração financeira A - Pagamento de qualquer espécie e desde que economicamente apreciáveis, feitos a (i) você, (ii) ao seu cônjuge/ companheiro ou a qualquer outro membro que resida com você, (iii) a qualquer pessoa jurídica em que qualquer destes seja controlador, sócio, acionista ou participante, de forma direta ou indireta, recebimento por palestras, aulas, atuação como proctor de treinamentos, remunerações, honorários pagos por participações em conselhos consultivos, de investigadores, ou outros comitês, etc. Provenientes da indústria farmacêutica, de órteses, próteses, equipamentos e implantes, brasileiras ou estrangeiras: - Novo Nordisk: Semaglutida. Outros relacionamentos Financiamento de atividades de educação médica continuada, incluindo viagens, hospedagens e inscrições para congressos e cursos, provenientes da indústria farmacêutica, de órteses, próteses, equipamentos e implantes, brasileiras ou estrangeiras: - Novo Nordisk: Semaglutida. Possui qualquer outro interesse (financeiro ou a qualquer outro título) que deva ser declarado tendo em vista o cargo ocupado na SBC, ainda que não expressamente elencado anteriormente: - Membro do Comitê Científico Instituto Vita Nova.
Carlos Eduardo dos Santos Ferreira	Nada a ser declarado
Charles Marques Lourenço	Outros relacionamentos Financiamento de atividades de educação médica continuada, incluindo viagens, hospedagens e inscrições para congressos e cursos, provenientes da indústria farmacêutica, de órteses, próteses, equipamentos e implantes, brasileiras ou estrangeiras: - Sanofi: Biomarin/Acondroplasia; PTC: NiemannPick B/Deficiência de AADC.
Cynthia Melissa Valerio	Declaração financeira A - Pagamento de qualquer espécie e desde que economicamente apreciáveis, feitos a (i) você, (ii) ao seu cônjuge/ companheiro ou a qualquer outro membro que resida com você, (iii) a qualquer pessoa jurídica em que qualquer destes seja controlador, sócio, acionista ou participante, de forma direta ou indireta, recebimento por palestras, aulas, atuação como proctor de treinamentos, remunerações, honorários pagos por participações em conselhos consultivos, de investigadores, ou outros comitês, etc. Provenientes da indústria farmacêutica, de órteses, próteses, equipamentos e implantes, brasileiras ou estrangeiras: - Novo Nordisk: Saxenda, Rybelsus, Ozempic; PTC: Waylivra; Amryt: Myalept. B - Financiamento de pesquisas sob sua responsabilidade direta/pessoal (direcionado ao departamento ou instituição) provenientes da indústria farmacêutica, de órteses, próteses, equipamentos e implantes, brasileiras ou estrangeiras. - Amryt: Metreleptina.
Dennys Esper Cintra	Nada a ser declarado
Francisco Antonio Helfenstein Fonseca	Nada a ser declarado
Gustavo Aguiar Campana	Nada a ser declarado
Henrique Tria Bianco	Nada a ser declarado
Josivan Gomes de Lima	Declaração financeira A - Pagamento de qualquer espécie e desde que economicamente apreciáveis, feitos a (i) você, (ii) ao seu cônjuge/ companheiro ou a qualquer outro membro que resida com você, (iii) a qualquer pessoa jurídica em que qualquer destes seja controlador, sócio, acionista ou participante, de forma direta ou indireta, recebimento por palestras, aulas, atuação como proctor de treinamentos, remunerações, honorários pagos por participações em conselhos consultivos, de investigadores, ou outros comitês, etc. Provenientes da indústria farmacêutica, de órteses, próteses, equipamentos e implantes, brasileiras ou estrangeiras: - Novo Nordisk: Semaglutida. Outros relacionamentos Financiamento de atividades de educação médica continuada, incluindo viagens, hospedagens e inscrições para congressos e cursos, provenientes da indústria farmacêutica, de órteses, próteses, equipamentos e implantes, brasileiras ou estrangeiras: - Novo Nordisk: Semaglutida.
Marcelo Heitor Vieira Assad	Declaração financeira A - Pagamento de qualquer espécie e desde que economicamente apreciáveis, feitos a (i) você, (ii) ao seu cônjuge/ companheiro ou a qualquer outro membro que resida com você, (iii) a qualquer pessoa jurídica em que qualquer destes seja controlador, sócio, acionista ou participante, de forma direta ou indireta, recebimento por palestras, aulas, atuação como proctor de treinamentos, remunerações, honorários pagos por participações em conselhos consultivos, de investigadores, ou outros comitês, etc. Provenientes da indústria farmacêutica, de órteses, próteses, equipamentos e implantes, brasileiras ou estrangeiras: - Novo Nordisk: Rybelsus. Outros relacionamentos Financiamento de atividades de educação médica continuada, incluindo viagens, hospedagens e inscrições para congressos e cursos, provenientes da indústria farmacêutica, de órteses, próteses, equipamentos e implantes, brasileiras ou estrangeiras: - Novo Nordisk: Rybelsus.
Maria Cristina de Oliveira Izar	Declaração financeira A - Pagamento de qualquer espécie e desde que economicamente apreciáveis, feitos a (i) você, (ii) ao seu cônjuge/ companheiro ou a qualquer outro membro que resida com você, (iii) a qualquer pessoa jurídica em que qualquer destes seja controlador, sócio, acionista ou participante, de forma direta ou indireta, recebimento por palestras, aulas, atuação como proctor de treinamentos, remunerações, honorários pagos por participações em conselhos consultivos, de investigadores, ou outros comitês, etc. Provenientes da indústria farmacêutica, de órteses, próteses, equipamentos e implantes, brasileiras ou estrangeiras: - Amgen: Repatha; Amryt Pharma: Lojuxta; AstraZeneca: Dapagliflozina; Aché: Trezor, Trezete; Biolab: Livalo; Abbott: Lipidil; EMS: Rosuvastatina; Eurofarma: Rosuvastatina; Sanofi: Praluent, Zympass, Zympass Eze, Efluelda; Libbs: Plenance, Plenance Eze; Novo Nordisk: Ozempic, Victoza; Servier: Acertamlo, Alertalix; PTCBio: Waylivra. B - Financiamento de pesquisas sob sua responsabilidade direta/pessoal (direcionado ao departamento ou instituição) provenientes da indústria farmacêutica, de órteses, próteses, equipamentos e implantes, brasileiras ou estrangeiras. - PTCBio: Waylivra; Amgen: Repatha; Novartis: Inclisiran, Pelacarsen; NovoNordisk: Ziltivekimab. Outros relacionamentos Financiamento de atividades de educação médica continuada, incluindo viagens, hospedagens e inscrições para congressos e cursos, provenientes da indústria farmacêutica, de órteses, próteses, equipamentos e implantes, brasileiras ou estrangeiras: - Novo Nordisk: Diabetes.
Maria Helane Costa Gurgel Castelo	Nada a ser declarado
Marileia Scartezini	Nada a ser declarado
Miguel Antonio Moretti	Nada a ser declarado
Natasha Slhessarenko Fraife Barreto	Nada a ser declarado
Raul Dias dos Santos Filho	Declaração financeira A - Pagamento de qualquer espécie e desde que economicamente apreciáveis, feitos a (i) você, (ii) ao seu cônjuge/ companheiro ou a qualquer outro membro que resida com você, (iii) a qualquer pessoa jurídica em que qualquer destes seja controlador, sócio, acionista ou participante, de forma direta ou indireta, recebimento por palestras, aulas, atuação como proctor de treinamentos, remunerações, honorários pagos por participações em conselhos consultivos, de investigadores, ou outros comitês, etc. Provenientes da indústria farmacêutica, de órteses, próteses, equipamentos e implantes, brasileiras ou estrangeiras: - Amgen: Evolocumabe; Astra Zenec:Dapagliflozina; Aché:/Rosuvastatina; PTC Therapeutics:Volanesorsen; Novartis: Inclisiran; Novo Nordisk: Semaglutide; Sanofi: Rosuvastatina; Libbs: Rosuvastatina. B - Financiamento de pesquisas sob sua responsabilidade direta/pessoal (direcionado ao departamento ou instituição) provenientes da indústria farmacêutica, de órteses, próteses, equipamentos e implantes, brasileiras ou estrangeiras. - Amgen: Evolocumabe; Sanofi: Alirocumabe; Novartis: Inclisiran; Esperion: Ácido Bempedóico; Novartis: Pelacarsen; Kowa: Pemafibrato. Outros relacionamentos Financiamento de atividades de educação médica continuada, incluindo viagens, hospedagens e inscrições para congressos e cursos, provenientes da indústria farmacêutica, de órteses, próteses, equipamentos e implantes, brasileiras ou estrangeiras: - Amgen: Colesterol; Ache: Colesterol; AstraZeneca: Diabete; Libbs: Colesterol; Novartis: Colesterol; Novo Nordisk: Diabetes; Eli-Lilly: Diabetes; Merck: Diabetes; Biolab: Colesterol.
Rayana Elias Maia	Declaração financeira A - Pagamento de qualquer espécie e desde que economicamente apreciáveis, feitos a (i) você, (ii) ao seu cônjuge/ companheiro ou a qualquer outro membro que resida com você, (iii) a qualquer pessoa jurídica em que qualquer destes seja controlador, sócio, acionista ou participante, de forma direta ou indireta, recebimento por palestras, aulas, atuação como proctor de treinamentos, remunerações, honorários pagos por participações em conselhos consultivos, de investigadores, ou outros comitês, etc. Provenientes da indústria farmacêutica, de órteses, próteses, equipamentos e implantes, brasileiras ou estrangeiras: - Amgen: PTC: Volanersorsen. Outros relacionamentos Financiamento de atividades de educação médica continuada, incluindo viagens, hospedagens e inscrições para congressos e cursos, provenientes da indústria farmacêutica, de órteses, próteses, equipamentos e implantes, brasileiras ou estrangeiras: - Amgen: PTC: Volanersorsen.
Renan Magalhães Montenegro Junior	Declaração financeira A - Pagamento de qualquer espécie e desde que economicamente apreciáveis, feitos a (i) você, (ii) ao seu cônjuge/ companheiro ou a qualquer outro membro que resida com você, (iii) a qualquer pessoa jurídica em que qualquer destes seja controlador, sócio, acionista ou participante, de forma direta ou indireta, recebimento por palestras, aulas, atuação como proctor de treinamentos, remunerações, honorários pagos por participações em conselhos consultivos, de investigadores, ou outros comitês, etc. Provenientes da indústria farmacêutica, de órteses, próteses, equipamentos e implantes, brasileiras ou estrangeiras: - Boheringer, AstraZeneca, Novo Nordisk, PTC, Nestlé, Amryt, Jansen. Outros relacionamentos Financiamento de atividades de educação médica continuada, incluindo viagens, hospedagens e inscrições para congressos e cursos, provenientes da indústria farmacêutica, de órteses, próteses, equipamentos e implantes, brasileiras ou estrangeiras: - Novo Nordisk, Amryt, PTC, Boheringer, Abbott.
Renato Jorge Alves	Declaração financeira A - Pagamento de qualquer espécie e desde que economicamente apreciáveis, feitos a (i) você, (ii) ao seu cônjuge/ companheiro ou a qualquer outro membro que resida com você, (iii) a qualquer pessoa jurídica em que qualquer destes seja controlador, sócio, acionista ou participante, de forma direta ou indireta, recebimento por palestras, aulas, atuação como proctor de treinamentos, remunerações, honorários pagos por participações em conselhos consultivos, de investigadores, ou outros comitês, etc. Provenientes da indústria farmacêutica, de órteses, próteses, equipamentos e implantes, brasileiras ou estrangeiras: - Boheringer, AstraZeneca, Novo Nordisk, PTC, Nestlé, Amryt, Jansen. Outros relacionamentos Financiamento de atividades de educação médica continuada, incluindo viagens, hospedagens e inscrições para congressos e cursos, provenientes da indústria farmacêutica, de órteses, próteses, equipamentos e implantes, brasileiras ou estrangeiras: - Novo Nordisk, Amryt, PTC, Boheringer, Abbott.
Roberta Marcondes Machado Figueiredo	Declaração financeira A - Pagamento de qualquer espécie e desde que economicamente apreciáveis, feitos a (i) você, (ii) ao seu cônjuge/ companheiro ou a qualquer outro membro que resida com você, (iii) a qualquer pessoa jurídica em que qualquer destes seja controlador, sócio, acionista ou participante, de forma direta ou indireta, recebimento por palestras, aulas, atuação como proctor de treinamentos, remunerações, honorários pagos por participações em conselhos consultivos, de investigadores, ou outros comitês, etc. Provenientes da indústria farmacêutica, de órteses, próteses, equipamentos e implantes, brasileiras ou estrangeiras: - PTC Therapeutics. Outros relacionamentos Financiamento de atividades de educação médica continuada, incluindo viagens, hospedagens e inscrições para congressos e cursos, provenientes da indústria farmacêutica, de órteses, próteses, equipamentos e implantes, brasileiras ou estrangeiras: - PTC Therapeutics.
Rodrigo Ambrosio Fock	Outros relacionamentos Financiamento de atividades de educação médica continuada, incluindo viagens, hospedagens e inscrições para congressos e cursos, provenientes da indústria farmacêutica, de órteses, próteses, equipamentos e implantes, brasileiras ou estrangeiras: - Biomarin.
Tânia Leme da Rocha Martinez	Nada a ser declarado

Sumário

1. Carta de Apresentação 5

2. Objetivos do Documento 6

3. Definição de Graus de Recomendação e Níveis de Evidência 6

4. Definição de Hipertrigliceridemia (>150mg/dL), Hipertrigliceridemia Grave (>500mg/dL) e Quilomicronemia (>1.000mg/dL) 7

4.1. Introdução 7

4.2. Definição de Hipertrigliceridemia 7

5. Definição de Quilomicronemia – Síndrome da Quilomicronemia Familiar e Síndrome da Quilomicronemia Multifatorial: Critérios Clínicos, Laboratoriais e Modo de Transmissão da Doença 7

5.1. Introdução 7

5.2. Conceitos 8

**5.2.1. Síndrome da Quilomicronemia Familiar** 8

**5.2.2. Síndrome da Quilomicronemia Multifatorial** 8

6. Epidemiologia da Síndrome da Quilomicronemia Familiar no Mundo e no Brasil 9

6.1. Definição de Síndrome da Quilomicronemia Familiar e Aspectos Clínicos 9

**6.1.1. Primeiros Casos de Síndrome da Quilomicronemia** 9

6.2. Epidemiologia da Síndrome da Quilomicronemia Familiar no Mundo 9

6.3. Epidemiologia da Síndrome da Quilomicronemia Familiar em Crianças 11

6.4. Epidemiologia da Síndrome da Quilomicronemia Familiar no Brasil 11

7. Manifestações Clínicas na Síndrome da Quilomicronemia Familiar, Diagnóstico Diferencial e Abordagem das Complicações 12

7.1. Manifestações Clínicas na Síndrome da Quilomicronemia Familiar 12

**7.1.1. Hipertrigliceridemia** 12

**7.1.2. Dor Abdominal e Pancreatite Aguda** 12

**7.1.3. Manifestações Neurológicas** 12

**7.1.4. Hepatosplenomegalia** 12

**7.1.5. Xantomas Eruptivos** 13

**7.1.6. Lipemia Retinalis** 13

**7.1.7. Qualidade de Vida** 13

**7.1.8. Escore Diagnóstico** 13

7.2. Diagnóstico Diferencial 13

**7.2.1. Síndrome da Quilomicronemia Multifatorial** 13

**7.2.2. Lipodistrofias** 14

7.3. Abordagem das Complicações da Síndrome da Quilomicronemia Familiar 14

**7.3.1. Pancreatite Aguda** 14

8. Diagnóstico Laboratorial da Síndrome da Quilomicronemia Familiar 15

8.1. Fase Pré-analítica (Orientações para Pacientes) 15

**8.1.1. Instruções para Coleta** 15

**8.1.2. Interferentes Pré-analíticos para Análise dos Triglicérides** 15

**8.1.3. Orientações para o Laboratório (Período Pré-analítico)** 15

8.2. Fase Analítica 15

**8.2.1. Metodologias que Avaliam os Quilomícrons** 15

**8.2.1.1. Ultracentrifugação** 15

**8.2.1.2. Aspecto do Soro** 15

**8.2.1.3. Eletroforese de Lipoproteínas** 16

**8.2.2. Metodologias que Avaliam os Triglicérides** 16

**8.2.3. Interferência no Resultado dos Triglicérides** 16

**8.2.4. Interferência dos Triglicérides em Outros Analitos** 16

**8.2.4.1. LDL-C** 16

**8.2.4.2. Plaquetas** 16

**8.2.4.3. Analitos com Avaliação Colorimétrica** 16

**8.2.4.4. Enzimas** 16

**8.2.4.5. Eletrólitos** 17

**8.2.5. Análises Laboratoriais para Diagnóstico Diferencial** 17

**8.2.5.1. Atividade da LPL com Heparina** 17

**8.2.5.2. Dosagem de Apolipoproteína C3 Plasmática** 17

8.3. Fase Pós-analítica 17

**8.3.1. Recomendações para as NOTAS nos Laudos Laboratoriais** 17

9. Aconselhamento Genético e as Etapas no Diagnóstico e Acompanhamento das Hipertrigliceridemias Graves 17

10. Orientação Nutricional na Quilomicronemia em Adultos, Crianças e Adolescentes 19

10.1. Classificação e Absorção dos Ácidos Graxos 19

10.2. Absorção das Gorduras 20

10.3. Tratamento Nutricional 20

**10.3.1. Gorduras** 20

**10.3.2. Triglicérides de Cadeia Média** 21

**10.3.3. Carboidratos** 21

**10.3.4. Álcool** 21

**10.3.5. Lactentes e Primeira Infância** 21

**10.3.6. Gestantes** 22

**10.3.7. Recomendações Gerais** 22

10.4. Exemplos de Cardápios 23

11. Aférese 27

11.1. Diagnóstico e Tratamento 27

11.2. Tratamento Não Medicamentoso 27

11.3. Tratamento Farmacológico 27

11.4. Aférese 27

11.5. Gestação e PH nos Pacientes com Síndrome da Quilomicronemia Familiar 28

12. Novas Terapêuticas para Tratamento da Síndrome da Quilomicronemia familiar 28

12.1. ApoC3 28

**12.1.1 Antissentido Anti-APOC3** 29

13. Aspectos Sociais, Psicológicos e Impacto Econômico da Doença 31

13.1. Aspecto Social na Síndrome da Quilomicronemia Familiar 32

13.2. Aspectos Psicológicos na Síndrome da Quilomicronemia Familiar 32

**13.2.1. Os Pais das Crianças com Diagnóstico de Síndrome da Quilomicronemia Familiar** 33

13.3. Para Reduzir os Impactos da Doença: Modos de Enfrentamento 33

**13.3.1. Modelos Ativos e Passivos de Enfrentamento: Foco no Paciente** 33

**13.3.2. Modelo Social de Enfrentamento: Foco nos Pares** 33

13.4. Custo-efetividade do Manejo de Riscos Psicossociais 31

14. Resumo das Recomendações 34

Referências 35

## Carta de Apresentação

A síndrome da quilomicronemia familiar (SQF) é uma forma grave de dislipidemia e compreende um conjunto de múltiplos sinais e sintomas causados pela deficiência da enzima lipoproteína lipase (LPL) ou de um de seus cofatores, comprometendo o metabolismo de triglicérides. Apresenta modo de herança autossômico recessivo e acomete cerca de 1 a 2 pessoas por milhão de indivíduos, mas pode ser mais frequente quando existe consanguinidade.

Existe grande desconhecimento sobre essa condição e, por esse motivo, o seu diagnóstico ocorre tardiamente, quando complicações já se instalaram. O paciente portador de SQF pode se apresentar com dores abdominais recorrentes, episódios de pancreatite, xantomas eruptivos, lipemia *retinalis* , hepatoesplenomegalia, além do aspecto cremoso do soro.

Nas formas clássicas e mais graves, os achados clínicos podem ser reconhecidos logo ao nascimento, ou ainda na infância, mas estes podem se apresentar em qualquer idade, especialmente nos portadores de novas mutações. Não é infrequente que o paciente com SQF tenha consultado várias especialidades médicas antes de ter seu diagnóstico firmado.

A apresentação clínica da SQF pode, ainda, ser indistinguível da síndrome da quilomicronemia multifatorial (SQM), mais frequente e que também tem uma base genética, mas sofre influência de fatores ambientais e ligados ao estilo de vida. Além disso, o quadro clínico pode ser secundário a condições como hipotireoidismo, diabetes não controlado, doenças renais, consumo abusivo de álcool e uso de certos medicamentos, o que dificulta ainda mais seu diagnóstico.

A confirmação genética, com um painel de genes causais para a SQF, é, atualmente, realizada em poucos centros em nosso meio. No entanto, quando uma mutação em homozigose em um dos genes causais, ou duas mutações em um mesmo gene (heterozigoto composto), ou em diferentes genes causais (heterozigoto duplo), for encontrada, confirma-se a condição de SQF, embora exista um percentual em que nenhuma mutação causal esteja presente. Algoritmos validados podem auxiliar na suspeição clínica da SQF e indicar quem deve realizar o teste genético.

O tratamento da SQF requer uma abordagem multiprofissional, incluindo nutricionista, psicólogo, entre outros profissionais de saúde, visando manter o bem-estar do indivíduo e o estado nutricional. Restrição do consumo de gorduras e de carboidratos simples, suplementação de vitaminas lipossolúveis e de ácidos graxos essenciais devem ser recomendados ao longo da vida. O suporte psicológico visa ajudar o indivíduo a conviver com as restrições dietéticas impostas.

O tratamento farmacológico convencional frequentemente se associa a uma resposta inferior a 20% na redução dos triglicérides, razão pela qual a grande esperança desses pacientes reside na chegada de novos fármacos ao Brasil, com benefício comprovado em reduzir os triglicérides nessa população. Situações peculiares no manuseio da SQF são a gestação e os episódios de pancreatite recorrentes, em que a mortalidade pode ser elevada e tratamentos individualizados são requeridos.

O intuito deste documento é conscientizar profissionais de saúde dos aspectos peculiares à SQF, capacitando-os no reconhecimento e na abordagem precoces da condição, mitigando o sofrimento do paciente e as complicações pelo retardo do diagnóstico.

Membros do Departamento de Aterosclerose da Sociedade Brasileira de Cardiologia e renomados especialistas de nosso país reuniram-se com o objetivo de transmitir as melhores informações científicas disponíveis sobre a SQF para melhoria da prática clínica, de forma clara e objetiva.

**Sinceramente,**

*Prof. Dra. Maria Cristina de Oliveira Izar*

*Prof. Dr. Raul Dias Santos*

*Prof. Dr. Antonio Carlos Palandri Chagas*

*Dr. Marcelo Heitor Vieira Assad*

*Coordenadores*

## 2. Objetivos do Documento

Este documento tem por objetivo conscientizar profissionais de saúde, especialmente cardiologistas, clínicos e endocrinologistas, de uma doença muito rara, subdiagnosticada, que causa intenso sofrimento às pessoas acometidas e que, até recentemente, não era diagnosticada, além de ser subtratada.

Escrito por especialistas na área, o Posicionamento Brasileiro sobre Síndrome da Quilomicronemia Familiar vem suprir uma lacuna no conhecimento dos dados epidemiológicos no mundo e em nosso país de manifestações clínicas, diagnóstico laboratorial e genético, e diagnóstico diferencial com outras formas de hipertrigliceridemia (HTG) graves. Além disso, o manejo nutricional, peculiar, e a abordagem de neonatos e crianças, gestantes e das complicações, como a pancreatite, são destacados neste documento. Vale ressaltar que, recentemente, tivemos em nosso país a aprovação de uma nova terapia antissentido anti-APOC3, com evidências de redução dos triglicérides e perspectivas de prevenir as complicações e melhorar a qualidade de vida dos pacientes.

## 3. Definição de Graus de Recomendação e Níveis de Evidência

Classes (graus) de recomendação:

Classe I – Condições para as quais há evidências conclusivas, ou, na sua falta, consenso geral de que o procedimento é seguro e útil/eficaz.

Classe II – Condições para as quais há evidências conflitantes e/ou divergência de opinião sobre segurança e utilidade/eficácia do procedimento.

Classe IIa – Peso ou evidência/opinião a favor do procedimento. A maioria aprova.

Classe IIb – Segurança e utilidade/eficácia menos bem estabelecida, não havendo predomínio de opiniões a favor.

Classe III – Condições para as quais há evidências e/ou consenso de que o procedimento não é útil/eficaz e, em alguns casos, pode ser prejudicial.

Níveis de evidência:

Nível A – Dados obtidos a partir de múltiplos estudos randomizados de bom porte, concordantes e/ou de metanálise robusta de estudos clínicos randomizados

Nível B – Dados obtidos a partir de metanálise menos robusta, a partir de um único estudo randomizado ou de estudos não randomizados (observacionais).

Nível C – Dados obtidos de opiniões consensuais de especialistas.

## 4. Definição de Hipertrigliceridemia (>150mg/dL), hipertrigliceridemia Grave (>500mg/dL) e Quilomicronemia (>1.000mg/dL)

### 4.1. Introdução

Primeiramente, antes de definirmos valores e classificar a HTG em discreta, moderada ou grave, devemos levar em consideração alguns fatores relevantes.

Para avaliação do perfil lipídico, recomenda-se estado metabólico estável e dieta habitual. Entretanto, orienta-se suspender o consumo etílico com 5 dias de antecedência.

Ao interpretar o perfil lipídico, deve-se levar em consideração a possibilidade de variação biológica intraindividual e possíveis variações interlaboratoriais. Tais variações podem atingir valores de 10% para o colesterol total, HDL-c e LDL-c e de até 25% para os triglicérides.^1^

As mais recentes diretrizes brasileiras de dislipidemia e de diabetes adotam como norma a dispensa do jejum para determinação dos triglicérides séricos. Contudo, na presença de concentração plasmática de triglicérides >400mg/dL, deve-se realizar uma nova dosagem, em jejum de 12h, diante da possível existência de HTG primária, na qual o jejum é necessário.^2 , 3^ Nessa situação, com HTG >400mg/dL, deixamos de utilizar a fórmula de Friedewald, habitualmente usada para cálculo das frações do colesterol.^4^ Algumas publicações sugerem maior risco cardiovascular relacionado à HTG pós-prandial.^5 , 6^ Em 2016, as diretrizes da *European Atherosclerosis Society* (EAS) e da *European Federation of Clinical Chemistry and Laboratory Medicine* retiraram a recomendação de jejum para coleta do perfil lipídico.^7^

A Diretriz Brasileira de Dislipidemia e Aterosclerose classifica laboratorialmente as dislipidemias conforme a Tabela 1 .

**Tabela 1:** – Classificação laboratorial das dislipidemias, segundo a Diretriz Brasileira de Dislipidemia e Aterosclerose ^2^

Hipercolesterolemia isolada	Aumento do LDL-c (>160mg/dL)
Hipertrigliceridemia isolada	Aumento dos triglicérides (>150mg/dL em jejum ou >175mg/dL sem jejum)
Hiperlipidemia mista	Aumento do LDL-c e dos triglicérides
Diminuição do HDLc	HDLc <40mg/dL em homens ou <50mg/dL em mulheres, com ou sem aumento dos triglicérides ou LDL-c

*HDLc: colesterol de lipoproteína de alta densidade; LDL-c: colesterol da lipoproteína de baixa densidade. Grau de Recomendação: I, Nível de Evidência: C.*

A classificação fenotípica de Fredrickson, demonstrada na Tabela 2 , baseia-se na separação eletroforética e/ou por ultracentrifugação das frações lipoproteicas. Embora tenha sido muito importante, atualmente, é pouco utilizada, exceto em serviços terciários e especializados no atendimento de dislipidemias. Como exemplo de sua relevância, citaremos pacientes com HTG, com diferentes classificações fenotípicas, de acordo com a anormalidade lipoproteica primária: SQF (tipo I), hiperlipidemia familiar combinada (tipo IIb), disbetalipoproteinemia (tipo III), HTG primária simples (tipo IV) e HTG com quilomicronemia (tipo V).^8 , 9^

**Tabela 2:** – Classificação fenotípica de Fredrickson ^8^

Classificação	Lipoproteína elevada
Tipo I	Quilomícrons
Tipo IIa	LDL
Tipo IIb	LDL e VLDL
Tipo III	IDL
Tipo IV	VLDL
Tipo V	Quilomícrons e VLDL

*IDL: lipoproteína de densidade intermediária; LDL: lipoproteína de baixa densidade; VLDL: lipoproteína de muito baixa densidade.*

### 4.2. Definição de Hipertrigliceridemia

Do ponto de vista laboratorial, define-se HTG quando a concentração plasmática de triglicérides estiver >150mg/dL. No entanto, quando a coleta do perfil lipídico não for realizada em jejum, considera-se HTG com valores >175mg/dL.^1^

Desse modo, podemos classificar as HTG^10^ em:

- Discretas: triglicérides plasmáticos >150mg/dL;
- Moderadas: entre 151 e 499mg/dL;
- Graves: entre 500 e 1.000mg/dL;
- Muito graves: >1.000mg/dL.

As HTG resultam do acúmulo de lipoproteínas ricas em ácidos graxos e glicerol (como VLDL, IDL e remanescentes). A principal anormalidade lipoproteica nas formas graves e muito graves é a quilomicronemia, definida como a presença de quilomícrons circulantes no estado de jejum. Com concentrações de triglicérides >1.000mg/dL, já se pode detectar a presença de quilomícrons no sangue; contudo, a quilomicronemia é mais provável quando essas concentrações ultrapassarem 1.500mg/dL. A relevância clínica das formas graves e muito graves da HTG deve-se à sua associação com um risco duas vezes maior de pancreatite aguda, cuja incidência aumenta em 3% para cada 100mg/dL >1.000mg/dL de trigliceridemia.^11^

## 5. Definição de quilomicronemia – síndrome da quilomicronemia familiar e síndrome da quilomicronemia multifatorial: critérios clínicos, laboratoriais e modo de transmissão da doença.

### 5.1. Introdução

Quilomicronemia é caracterizada por acúmulo de quilomícrons na circulação e aumento importante da concentração plasmática de triglicérides.

Quanto maior a concentração de triglicérides plasmático, maior o risco de ocorrer pancreatite. Entretanto, casos de valores >1.000mg/dL ou quadro de HTG muito grave são mais propensos a desenvolver pancreatite aguda. Essas alterações laboratoriais devem ser associadas a alterações clínicas que estariam presentes desde a infância ou adolescência, para suspeitar-se do diagnóstico de SQF. Entre essas alterações, estariam: lipemia *retinalis* , xantomas eruptivos, hepatoesplenomegalia e, principalmente, pancreatites agudas, que ajudariam a confirmar o diagnóstico de SQF.^12^

A relevância clínica das formas graves e muito graves da HTG deve-se à sua associação com um risco duas vezes maior de pancreatite aguda. Além de ser uma emergência médica potencialmente fatal, a pancreatite aguda também pode levar a várias complicações clínicas, tais como pancreatite crônica, insuficiência pancreática e diabetes *mellitus* .^11 , 13^

Os quilomícrons são formados pela incorporação dos lípides provenientes da dieta com as apolipoproteínas (A1, A2, A4, B48, C2, C3 e E) e secretados na linfa mesentérica.^14^ A LPL é uma enzima que se localiza na superfície endotelial dos capilares do tecido adiposo e de músculos, que, ao ser ativada, inicia o processo de hidrólise dos triglicérides dos quilomícrons, gerando os remanescentes de quilomícrons. A atividade da LPL é modulada pela ação da APOC2 e da APOA5, que atuam como cofatores na sua ativação; pelo fator de maturação da lipase 1 (LMF1; do inglês, *lipase maturation factor 1* ), necessário para a produção de LPL em adipócitos e miócitos; e pela GPIHBP1 ( *glycosylphosphatidylinositol anchored high-density lipoprotein binding protein 1* ), que transporta LPL do espaço intersticial para o lúmen capilar. Qualquer alteração na função e/ou ativação da LPL resulta em aumento da meia-vida dos quilomícrons na corrente sanguínea e, consequentemente, quilomicronemia.^14^

Existem duas formas distintas de quilomicronemia: a SQF e a SQM. Estas são, respectivamente, os protótipos das condições monogênicas e poligênicas subjacentes à HTG grave de origem genética. Estima-se que a quilomicronemia possa ser encontrada em 1:600 adultos, mas os pacientes com SQF representam apenas 5% desses indivíduos.^15^

A diferenciação entre as duas doenças pode ser feita pelas características clínicas e/ou laboratoriais de seus portadores. Pacientes com SQF costumam se apresentar geralmente com pancreatite, e os com SQM são mais propensos a terem doença cardiovascular aterosclerótica. O diagnóstico precoce e correto das duas entidades é fundamental para o sucesso terapêutico e a prevenção de mortalidade.

Devido à sua considerável sobreposição fenotípica, as duas formas são difíceis de distinguir, e ainda existem várias perguntas sem resposta relacionadas à prevalência, às características clínicas e genéticas e ao manuseio clínico.

### 5.2. Conceitos

#### 5.2.1. Síndrome da Quilomicronemia Familiar

A SQF é uma doença metabólica grave e muito rara, caracterizada por quilomicronemia associada a episódios recorrentes de dor abdominal e/ou pancreatite.

A estimativa mundial é que a SQF ocorra em 1 para cada 500.000 a 1.000.000 pessoas.^15 , 16^ Frequentemente, manifesta-se na infância ou adolescência e tem sido descrita em todas as etnias, com maior prevalência em algumas áreas geográficas, como Quebec, devido ao efeito fundador.^17^

Também chamada de hiperlipoproteinemia tipo I de Fredrickson,^15^ a SQF é um distúrbio lipídico monogênico, autossômico recessivo, cujo diagnóstico é baseado na detecção de mutações raras, bialélicas (homozigoto ou heterozigoto composto) na LPL (>80% dos casos) e em outros genes que codificam as proteínas necessárias para sua atividade, tais como *APOC2* , *APOA5* , *GPIHBP1* e *LMF1* , levando a uma redução drástica da depuração dos quilomícrons.^15 , 18^ Normalmente, esses pacientes têm pouca resposta a medicamentos para reduzir os triglicérides plasmáticos, de modo que seu tratamento representa um desafio clínico. A pedra angular da terapia da SQF é representada por uma redução drástica da ingestão de gordura (8 a 10% do total de calorias), terapêutica esta difícil de ser mantida ao longo do tempo. A adesão a restrições alimentares dessa magnitude ao longo da vida do paciente é difícil, afeta negativamente a qualidade de vida e não elimina completamente o risco de pancreatite em todos os pacientes. A pancreatite aguda recorrente ocorre em 50% dos pacientes com SQF; a taxa geral de mortalidade associada é de 5 a 6%, mas aumenta para 30% em subgrupos de pacientes que evoluem com necrose pancreática ou falência persistente de múltiplos órgãos.^15 , 19^

#### 5.2.2. Síndrome da Quilomicronemia Multifatorial

A SQM, também chamada de hiperlipoproteinemia tipo V de Fredrickson, é um distúrbio lipídico oligogênico ou poligênico agravado pela presença de comorbidades conhecidas por aumentar a trigliceridemia (diabetes não controlado, hipotireoidismo, gravidez, obesidade), fatores ambientais (consumo abusivo de álcool e dieta rica em gorduras e açúcares simples) e certos medicamentos, como glicocorticoides, etinilestradiol e neurolépticos.^20^ A ocorrência de SQM tende a crescer de forma linear com o aumento da prevalência de obesidade, síndrome metabólica e diabetes tipo 2 na população mundial. Nos portadores dessa síndrome, a quilomicronemia é flutuante e, na grande maioria, se manifesta tardiamente.^15^ Respondem bem a modificações no estilo de vida e ao tratamento de fatores secundários, com boa resposta às farmacoterapias redutoras de triglicérides. Caracteriza-se por um risco aumentado de pancreatite aguda, mas o *odds ratio* estimado em 50 é claramente menor que o *odds ratio* de 360 relatado em pacientes com SQF.^16 , 21^

É possível diferenciar essas duas formas de quilomicronemia com base na eletroforese de lipoproteínas ou na ultracentrifugação (presença de lipoproteína de densidade muito baixa [VLDL] e quilomícrons na SQM; apenas quilomícrons na SQF). O procedimento padrão-ouro atual para identificar pacientes com SQF continua sendo o teste genético ou a atividade da LPL pós-heparina.^22^ Como o tratamento dessas duas formas de quilomicronemia é muito diferente, é importante fazer um diagnóstico apropriado. Novas terapias, como inibidores da APOC3, estão em desenvolvimento para diminuir os triglicérides em indivíduos com SQF.^23^

## 6. Epidemiologia da Síndrome da Quilomicronemia Familiar no Mundo e no Brasil

### 6.1. Definição de Síndrome da Quilomicronemia Familiar e Aspectos Clínicos

A SQF é uma doença herdada, muito rara, acometendo cerca de 1-2:1.000.000 de indivíduos, com modo de transmissão autossômico recessivo, caracterizada por concentrações muito elevadas de triglicérides (em geral, muito acima de 1.000mg/dL), acompanhada de soro lipêmico com aspecto cremoso, lipemia *retinalis* , dores abdominais recorrentes, xantomas eruptivos, episódios de pancreatites de repetição, distúrbios cognitivos e neurológicos e comprometimento da qualidade de vida e da sociabilidade.^24^

As manifestações clínicas, no entanto, aparecem em frequência variável nos portadores de SQF. Os xantomas eruptivos foram descritos em 17 a 23%; a lipemia *retinalis* , em 4 a 36%; hepatoesplenomegalia ou esplenomegalia isolada, em 12% a 25%; dor abdominal, em 26 a 63%; pancreatite, em 60% a 88%; com múltiplas pancreatites, em 17 a 48% dos pacientes com SQF.^23 , 25 , 26^ O aspecto do soro é importante para diferenciar situações que causam o aumento do glicerol livre no sangue, levando a uma superestimação dos níveis de triglicérides, sem a turvação do soro, observado após permanecer por 12h em geladeira e excluindo-se causas de aumento de hiperglicerolemia (exercício físico recente, ingestão alcóolica, doença hepática aguda, diabetes descompensado, nutrição parenteral ou medicação intravenosa contendo glicerol).^27 , 28^

Na SQF, a HTG grave resulta da incapacidade da metabolização dos triglicérides e outras gorduras. As gorduras são absorvidas pelo intestino delgado, no qual os quilomícrons são formados. Quando a LPL tem sua atividade normal, ela participa da hidrólise dos triglicérides de quilomícrons em ácidos graxos livres, por meio da via dependente da LPL.^20^ Na SQF, os quilomícrons, os quilomícrons remanescentes e as lipoproteínas ricas em triglicérides não podem ser metabolizados e se acumulam no plasma. Dessa forma, o acúmulo de triglicérides pode prejudicar o fluxo sanguíneo pancreático e ativar processos inflamatórios, resultando em pancreatite aguda.^19 , 29 - 30^

O papel da LPL e de seus cofatores é crucial para se entender o metabolismo das lipoproteínas ricas em triglicérides.^24^ A síntese de LPL ocorre no intracelular de adipócitos e células musculares lisas. Ela é produzida como um monômero, e o fator de maturação da lipase (LMF-1) é necessário para que ocorra a correta homodimerização da LPL. Após esse passo, a GPIHBP1, uma glicoproteína envolvida no transporte da LPL no lúmen dos capilares, facilita a ancoragem da LPL aos capilares endoteliais, em que hidrolisa os triglicérides dos quilomícrons e de VLDL (do inglês, *very-low-density lipopprotein* ). As apolipoproteínas C2 e A5 participam como cofatores na ativação da LPL. A hidrólise dos triglicérides dessas lipoproteínas libera ácidos graxos livres e monoglicerídeos, que são transportados aos miócitos ou adipócitos, em que são utilizados para produção de energia ou para estocar lípides.^24^

Mutações em 5 genes diferentes têm sido implicadas no desenvolvimento de SQF, todas com efeito sobre a atividade da LPL, responsável pela remoção dos triglicérides dos quilomícrons e de outras lipoproteínas ricas em triglicérides na circulação, quebrando-os em ácidos graxos livres. Pacientes com SQF têm perda de função do gene *LPL* levando a níveis de quilomícrons extremamente elevados na circulação e, portanto, HTG grave. Outros genes também foram descritos como cofatores na ativação da LPL, a saber: *APOC2* , *APOA5* , *LMF1* e *GPIHBP1* .^15^

#### 6.1.1. Primeiros Casos de Síndrome da Quilomicronemia Familiar

A primeira descrição da SQF foi feita por Gaskins *et al* .,^28^ em 1953, quando acompanhou três casos em uma família de oito pessoas, com diagnóstico de hiperlipoproteinemia familiar idiopática. Os pacientes apresentavam soro leitoso, com triglicérides muito elevados, e a dieta restrita em gorduras, seguida da administração de heparina endovenosa, reduzia muito os triglicérides, sugerindo que o defeito fosse relacionado à remoção de triglicérides da circulação.^28^

Em 1960, essa família foi estudada e suspeitou-se que a LPL, enzima ancorada ao endotélio vascular e liberada da parede pela heparina, seria a responsável pelo defeito lipídico.^31^ Ao estudar três irmãos afetados pela condição, os autores também sugeriram que outro defeito, além da LPL, poderia causar a então chamada síndrome da hiperlipoproteinemia familiar idiopática.

## 6.2. Epidemiologia da Síndrome da Quilomicronemia Familiar no Mundo

Por ser uma doença muito rara, os relatos de especialistas contribuem grandemente nas estimativas de prevalência. Hegele *et al* .^32^ reportaram que, de uma série de amostras biológicas de 381 pacientes com triglicérides >1.000mg/dL, quatro pacientes (ou 1%) apresentavam duas mutações com largo efeito por perda de função em ambos os alelos do gene da *LPL* , caracterizando a clássica deficiência da LPL autossômica recessiva. Quando foram considerados pacientes com mutações em ambos os alelos dos quatro genes ditos menores (do inglês, *minor* ), que modulam a atividade da LPL – a saber, apolipoproteína C2 ( *APOC2* ), apolipoproteína A5 ( *APOA5* ), fator de maturação da lipase 1 ( *LMF1* ), e no gene *glycoprotein-inositol high-density lipoprotein–binding protein 1* ( *GPIHBP1* ) –, foram encontrados outros quatro pacientes, ou seja, mais 1%.^33 , 34^

Pacientes com duas mutações no gene *LPL* ou em seus genes reguladores, os heterozigotos compostos, possuem duas mutações diferentes com perda de função, e aqueles com duas mutações em heterozigose em dois genes causais distintos, ou seja, heterozigotos duplos, somaram mais 1%.^33 , 34^

Assim, estimou-se que cerca de 3% dos pacientes com HTG grave (triglicérides ≥1.000mg/dL) dessa amostra tinham mutações em ambos os alelos dos genes que codificam a LPL ou uma das proteínas moduladoras de sua atividade. Esses pacientes podem ser homozigotos, heterozigotos compostos ou heterozigotos duplos. Essas condições foram descritas entre os franco-canadenses da província de Quebec, onde a porcentagem de pacientes com dois alelos mutantes é maior devido a efeito fundador. Tal prevalência pode parecer pequena se comparada à imensa maioria de pacientes com HTG grave. Contudo, na ausência de teste genético, não se pode separar a SQF (tipo I) da SQM (ou tipo V) em pacientes com triglicérides ≥1.000mg/dL. Na verdade, a maioria dos pacientes com HTG grave (97%) apresenta uma base genética, ainda não muito bem esclarecida, que inclui heterozigose para uma mutação com perda de função no gene *LPL* ou seus cofatores e outras variantes de menor impacto, ou, ainda, possuem forte componente de fatores ambientais. Há, assim, uma base poligênica com muitas variantes possíveis em diferentes combinações que estão super-representadas entre esses pacientes com HTG graves, que perfazem a forma multifatorial (SQM).^32 - 37^

Os dados de Surendran *et al* .^33^ mostram que dos cinco genes causais, 34% das mutações encontradas foram no gene *LPL* .^33^ Comparando-se os dados clínicos e laboratoriais de pacientes com SQF de várias etiologias genéticas, as SQF decorrentes de defeito no gene *LPL* são fenotipicamente muito semelhantes aos defeitos não relacionados ao gene *LPL* . No entanto, pacientes com defeito no gene *LPL* apresentam menor atividade da lipase pós-heparina e tendem a ter triglicérides mais elevados. Já as concentrações de LDL-C são, em geral, maiores entre os portadores de defeitos em genes que não a *LPL* .^38^

Utilizando-se dos dados do *National Health and Nutrition Examination Survey* (NHANES) de 2001 a 2006, estimou-se a prevalência de HTG grave entre 5.680 adultos com mais de 20 anos, que dispunham de resultados de triglicérides obtidos em jejum. Nesses, a prevalência de triglicérides entre 500 e 2.000mg/dL foi de 1,7% (87 indivíduos), e >2.000mg/dL, encontrou-se apenas em três indivíduos.^20^ Esses dados extrapolados para a população norte-americana dariam uma estimativa de 3.357.214 de adultos com HTG grave com triglicérides entre 500 e 2.000mg/dL e 81.877 ≥2.000mg/dL.^39^

Um estudo retrospectivo transversal avaliou pacientes da *Oregon Health & Science University* de julho de 2012 a julho de 2017.^40^ Foram revisados os dados eletrônicos dos pacientes atendidos naquele período baseando-se em quatro critérios: triglicérides ≥880mg/dL, história de pancreatite aguda, ausência de causas secundárias de HTG e resposta insuficiente (<20%) à terapia redutora de triglicérides. Quando três desses quatro critérios eram preenchidos, considerava-se provável SQF. Na presença de quatro critérios, ou se houvesse confirmação da presença de mutação em genes causais, considerava-se diagnóstico definitivo de SQF. Dos 2.342.136 dados eletrônicos avaliados, 578 pacientes tinham triglicérides ≥880mg/dL (0,025%), dos quais 86 tinham história documentada de pancreatite. Cinco pacientes que preencheram os critérios de SQF foram identificados e três obtiveram confirmação genética, resultando em uma prevalência estimada de 1-2 por 1.000.000 de pessoas. Já a SQM foi identificada em 186 pacientes, correspondendo a uma prevalência estimada de 1 em 12.000 pessoas. Houve 5.181 casos de pancreatite (0,22% de toda a coorte), 86 destes ocorreram em indivíduos com triglicérides ≥880mg/dL (1,7% dos casos de pancreatite). As taxas de pancreatite nesta subamostra se elevaram para 6,5%, 100%, e 17,8% entre pacientes com SQM, SQF e HTG de causas secundárias, respectivamente.^40^

Em outro estudo retrospectivo, com dados de 70.201 pacientes atendidos na *Cleveland Clinic Lipid Center* de janeiro a dezembro de 2006, usando o valor de corte de triglicérides ≥750mg/dL e a presença de pancreatite prévia como critérios, foram encontrados 369 indivíduos que perfaziam essas condições. Desses, 333 correspondiam a causas secundárias, ou os dados eram inconsistentes ou faltantes e foram excluídos. Dos 36 participantes restantes, 14 tinham critérios de SQF.^41^ Segundo os autores, nessa coorte de SQF, a prevalência encontrada foi de pelo menos 1:5.000, com base em critérios diagnósticos estabelecidos.^22 , 42^ Esses dados representam uma prevalência >20-200 vezes os dados de prevalência de relatos anteriores. Um rastreamento de pacientes com triglicérides ≥1.000mg/dL e história de pancreatite a partir dos dados eletrônicos da *North Texas Division of the Baylor Scott & White Health System* , no período de setembro de 2015 a setembro de 2016, evidenciou que de 297.891 pacientes adultos com valores disponíveis de triglicérides, 334 (0,11%) tinham valores de triglicérides ≥1.000mg/dL, e 30 (9%) desses tiveram pancreatite. Desses, seis casos foram excluídos devido a causas secundárias. Dos 24 casos restantes, os maiores valores médios de triglicérides encontrados foram de 3.085 +/- 1.211mg/dL. Assim, o rastreio eletrônico dos triglicérides ≥1.000mg/dL e a história de pancreatite permitiram afastar 99,99% das HTG graves, restando 24 casos em que a SQF não pôde ser excluída, sugerindo uma prevalência de 1 em 12.413 pessoas. Uma importante limitação aos dados desses dois estudos foi a indisponibilidade de confirmação genética.^43^

Outro estudo em Quebec avaliou a aparência do plasma e classificou os pacientes de acordo com os valores de triglicérides, a provável etiologia e as características bioquímicas. Um total de 354 pessoas com plasma lactescente foi comparado a 482 pacientes com plasma claro, mas com triglicérides >5mmol/L (cerca de 440mg/dL) e com 364 controles normolipidêmicos (triglicérides <2mmol/L, ou <176mg/dL). Os autores observaram que o plasma lactescente representava um grupo heterogêneo de pacientes de alto risco e, entre aqueles, foram encontrados 28 pacientes com SQF, 62 com disbetalipoproteinemia (por defeitos no gene *APOE* , E2E2), 182 com HTG tipo IV e 82 pacientes com HTG tipo V. Do ponto de vista clínico, quanto maiores as concentrações de triglicérides e quanto mais leitoso o plasma, houve maior risco de pancreatites. O exame visual do plasma e o fenótipo clínico foram úteis para estabelecer o risco cardiometabólico dos pacientes, sendo o reconhecimento do plasma lactescente uma ferramenta diagnóstica simples que pode auxiliar na identificação daqueles de maior risco.^44^

Já os dados de Dron et al.^36^ sugeriram que apenas 1 a 2% dos pacientes com HTG ≥1.000mg/dL tinham SQF, sendo a maioria dos demais SQM. Analisando variantes raras e comuns em duas coortes independentes de 251 e 312 pacientes caucasianos com HTG grave e, sequenciando-se por NGS ( *next generation sequencing* ) 73 genes e 185 polimorfismos de nucleotídeo único (SNPs) associados com hiperlipidemia, além dos cinco genes causais para SQF ( *LPL* , *APOC2* , *GPIHBP1* , *APOA5* , e *LMF1* ), encontrou-se que 1,1% tinha variantes bialélicas raras, 14,4% tinham variantes raras em heterozigose e 32% dispunham de um acúmulo de variantes comuns, ou seja, um escore poligênico elevado, e 52% permaneceram não identificados. Os pacientes com HTG grave eram 5,77 vezes mais propensos a carrear uma dessas variantes de suscetibilidade do que os controles.^36^

Um relato de SQF em uma família com três membros afetados que apresentavam HTG grave e episódios de pancreatite teve seu painel genético analisado.^45^ O caso índice era de uma mulher com múltiplos episódios de pancreatite, um deles durante a gestação e que necessitou de plasmaférese. Foi também avaliada a prevalência de HTG grave a partir de dados populacionais obtidos de um laboratório de referência onde foram afastadas causas secundárias (207.926 participantes, com idade de 58 anos, 52% mulheres) e diabetes. A mulher de 28 anos tinha HTG e pancreatites recorrentes, com início aos 3 meses de idade. Obtinha controle razoável dos triglicérides com dieta pobre em gorduras até os 20 anos, quando passou a apresentar episódios recorrentes de pancreatites, e triglicérides em jejum >2.000mg/dL, necessitando de múltiplas hospitalizações, a despeito do tratamento. Além da dieta restrita, recebeu fenofibrato, triglicérides de cadeia média, ácido nicotínico e ácidos graxos ômega-3, sem resposta satisfatória. Durante a gestação, aos 30 anos, necessitou de plasmaférese semanal ou a cada 2 semanas, até o parto. Seus pais e uma irmã tinham HTG e história de pancreatite. A paciente era heterozigótica composta para mutações no gene *LPL* (deleção c.708delA [p.G237fs*15] e variante *missense* c.644G.A [p.G215E]), que comprometem a função da LPL. O pai apresentava a variante com deleção c.708delA (p.G237fs*15), a mãe e a irmã, a variante c.644G.A (p.G215E). A análise de 207.926 indivíduos da população encontrou 25 com triglicérides em jejum >2.000mg/dL, sem evidências de causas secundárias, estimando-se uma prevalência de 120/1 milhão de indivíduos.^45^

Em outro estudo, a prevalência de SQF foi avaliada em uma área basicamente rural na região central do estado de Nova York com uma população estimada em 870.000 habitantes. Analisando-se os dados de prontuários eletrônicos de 385.000 pacientes, foram encontrados 998 com triglicérides >750mg/dL, sendo que 994 foram eliminados por causas secundárias de HTG, resposta satisfatória ao tratamento ou por dados incompletos. Restaram 4 pacientes com critérios de SQF. Assim, a chance de encontrar 4 casos em 870.000 seria de 0,01, o que sugere que a prevalência de 1/1.000.000 seja subestimação. Atribuiu-se a alta prevalência a um provável efeito fundador.^46^

A prevalência de SQF foi também avaliada de maneira retrospectiva a partir de dados de prontuários eletrônicos de 7.699.288 pacientes da Universidade da Califórnia do Sul, com triglicérides >880mg/dL, pelo menos um episódio de pancreatite, resposta à terapia hipolipemiante <20% e afastadas causas secundárias. Essa análise mostrou uma prevalência de SQF de 0,26 a 0,65 por milhão de indivíduos.^47^

Finalmente, a prevalência de SQF foi determinada em um centro de atenção quaternária.^48^ Foram revistos dados de 1.627.763 pacientes atendidos no Hospital Johns Hopkins de 2013 a 2017. O critério para SQF incluiu pacientes com a) triglicérides >750mg/dL em pelo menos uma dosagem, b) história de pancreatite aguda, dores abdominais recorrentes não explicadas e/ou história familiar de HTG e c) ausência de causas secundárias de HTG. Foram encontrados 21 casos de SQF e 89 de causas secundárias de HTG. A prevalência de SQF nesse estudo foi de 13:1.000.000 (IC95% 8-20).^48^

## 6.3. Epidemiologia da Síndrome da Quilomicronemia Familiar em Crianças

Em crianças, não existem dados acerca da prevalência de HTG grave e de SQF. Análise retrospectiva de prontuários eletrônicos de um hospital pediátrico terciário ( *Children’s Medical Center* , *Dallas* ) e dos dados do NHANES de 2000-2015 foram pesquisados. De 30.623 crianças do *Children’s Medical Center* , 36 (1 em 1.000) tinham triglicérides com elevação extrema (≥2.000mg/dL), e um terço dessas desenvolveu pancreatite aguda. A maioria desses casos correspondia a causas secundárias de HTG, sendo a prevalência estimada de SQF em crianças de 1:6.000 em um centro de atenção terciária e de 1:300.000 em crianças da população geral. Dos dados do NHANES 2.000-2015, nenhuma das 2.362 crianças preencheu os critérios de HTG extrema, enquanto, nos adultos do NHANES, a prevalência estimada era de 0,02%.^49^

## 6.4. Epidemiologia da Síndrome da Quilomicronemia Familiar no Brasil

Em nosso país, os relatos de casos de SQF são muito escassos. São descritos casos de SQF em várias regiões do país, com maior concentração de casos em regiões onde existe efeito fundador, especialmente na região Nordeste, porém não foram encontradas publicações sobre o tema. Foram identificadas apresentações em congressos, com publicações em anais dos eventos.

O primeiro relato de caso foi de uma criança do sexo masculino, com 3 anos de idade, que apresentou soro lipêmico e concentrações de triglicérides plasmáticos de 25.000mg/dL aos 3 meses de idade com aleitamento materno exclusivo. Aos 3 anos, apresentava hepatoesplenomegalia e, após dieta restrita em gorduras e leite desnatado, os triglicérides foram para 990mg/dL. Apresentava atividade da LPL nula e foi detectada a mutação G188E no éxon 5 da lipoproteína lipase em homozigose na criança, e em heterozigose nos pais.^50^

Outro relato consiste em dois casos de crianças, uma com 21 dias e a outra com 4 meses e 15 dias de vida. Em ambos os casos, a HTG foi achado casual realizado a partir do aspecto descrito como xantocrômico do sangue durante coleta de exames. Os níveis de triglicérides ao diagnóstico eram de 18.019mg/dL e 5.333mg/dL, respectivamente. Após intervenção dietética hospitalar e ambulatorial, os menores níveis alcançados de triglicérides foram de 602mg/dL e 615mg/dL. Um dos pacientes evoluiu com episódios recorrentes de pancreatite aguda, relacionados a níveis elevados de triglicérides.^51^

Outro caso descrito é de uma criança de 15 meses com quilotórax e perfil lipídico sugestivo de SQF, com triglicérides >1.000mg/dL, sem episódios de pancreatite, em paciente oriundo de região no Rio Grande do Norte.^52^ Outro caso identificado foi o de uma mulher de 45 anos com HTG grave e diabetes e xantomas eruptivos exuberantes.^53^

Dois outros casos de irmãos com SQF com confirmação genética de mutação no gene *LPL* foram identificados, oriundos de região do interior da Paraíba.^54^ Outro relato de caso é de uma criança de 45 dias de vida com queixa de vômitos e irritabilidade, triglicérides de 6541mg/dL e com análise molecular alterada em 3 variantes: Chr8:19.811.733 G>A, promovendo a substituição do aminoácido glicina no códon 215 por glutamato (p.Gly215Glu); Chr8:19.813.385 G>A, promovendo a substituição do aminoácido arginina no códon 270 por histidina (p.Arg270His); e Chr8:19.811.823 T>C, promovendo a substituição do aminoácido isoleucina no códon 245 por treonina (p.Ile245Thr). A conduta dietética foi leite desnatado, triglicérides de cadeia média (TCM) e vitaminas A, D, E e K. Após a alta, foi mudada a dieta, recebendo fórmula láctea que levou a aumento dos triglicérides (11760mg/dL). Foi instituído jejum e restituída a conduta dietética anterior, o que permitiu controle razoável da trigliceridemia, crescimento e ganho ponderal adequados.^55^

Lima et al.^56^ publicaram recentemente 12 casos de SQF em pacientes com mutação em homozigose em região intrônica do gene *GPIHBP1* , todos com HTG grave (2351mg/dL [885-20600mg/dL]), HDL-c baixo (18mg/dL [5-41mg/dL]) e 33% com episódios de pancreatite aguda. Todos os pacientes eram oriundos de cidades do Nordeste do país, sugerindo um efeito fundador.^56^

A falta de critérios clínicos padronizados, a semelhança com a SQM, a escassez de testes genéticos confirmatórios, a falta de registros nacionais e internacionais e, ainda, o efeito fundador dos genes causais fazem com que os dados de prevalência da SQF sejam tão variáveis de estudo para estudo.

## 7. Manifestações clínicas mais frequentes na síndrome da quilomicronemia familiar

### 7.1. Manifestações Clínicas na Síndrome da Quilomicronemia Familiar

As manifestações clínicas das formas monogênicas de quilomicronemia, em geral, ocorrem na infância ou início da vida adulta. Entretanto, por se tratar de uma doença relativamente rara, atrasos no diagnóstico são comuns, fazendo com que o mesmo ocorra na vida adulta, quando as complicações já estão estabelecidas.^2^

Uma revisão de base de dados do estudo APPROACH demonstrou que a média de idade ao diagnóstico era 24 anos, com mais da metade dos 66 pacientes tendo sido diagnosticados após os 20 anos de idade. Ao diagnóstico, 75% já haviam apresentado o primeiro episódio de pancreatite.^57^ Outras séries descrevem uma média de avaliação por cinco médicos diferentes antes de o diagnóstico vir a ser estabelecido.^58^

Estes reforçam a importância do diagnóstico precoce e oportuno. As principais manifestações clínicas da SQF estão descritas a seguir.

#### 7.1.1. Hipertrigliceridemia

Na avaliação laboratorial, os pacientes afetados têm hiperquilomicronemia, apresentando-se com grande aumento dos triglicérides – em geral, na faixa de 1.500 a 5.000mg/dL –, às custas de aumento de VLDL-colesterol ( *very low-density lipoproteins* ) e, principalmente, quilomícrons circulantes. Como uma pequena quantidade de colesterol também é transportada e encontra-se presente nos quilomícrons, o colesterol total pode estar elevado, em geral em uma proporção triglicérides/colesterol <5:1. Muitos pacientes têm aumento moderado da VLDL-c, mas com níveis de LDL-colesterol e apolipoproteína B <100mg/dL.^21^

Na Classificação de Friedrickson, apesar de o fenótipo do tipo V ser o mais comum, o tipo I parece ser mais específico para o diagnóstico de SQF em adultos. Em crianças, o fenótipo tipo 1 é mais frequentemente observado.^21^

Habitualmente, a HTG grave dos pacientes com SQF apresenta pouca resposta a fibratos e/ou demais medicações hipolipemiantes. Para esses casos, que constituem enorme desafio na prática clínica diária, a principal arma terapêutica é a dieta com redução drástica de ingestão de gordura (8 a 10% do total de calorias diárias). Não raramente, a severidade da dieta dificulta a adesão dos pacientes ao tratamento a longo prazo e impacta de forma significativa a sua qualidade de vida.^5 , 21^

#### 7.1.2. Dor Abdominal e Pancreatite Aguda

Dor abdominal recorrente é uma manifestação presente em até 50% dos pacientes e não é necessariamente associada com os quadros de pancreatite aguda, podendo ser incapacitante.^27^

Em média, a partir do nível de triglicérides >1.000mg/dL, ocorre aumento da incidência de 3% no risco de pancreatite a cada elevação de 100mg/dL de triglicérides.^59^

Um estudo canadense comparou um grupo de 25 indivíduos com SQF e outro com 36 pacientes com SQM e demonstrou que, apesar de apresentarem mesmos níveis médios de triglicérides, o grupo com SQF apresentou 10 vezes mais risco (60 x 6%) de pancreatite.^22^ Provavelmente, isso decorre de um maior tempo de exposição à hiperquilomicronemia, que, no caso da SQF, tende a ocorrer nos primeiros anos de vida.

Os múltiplos episódios de pancreatite aguda e a severidade das restrições alimentares impactam de forma negativa a qualidade da vida do paciente e aumentam consideravelmente a morbimortalidade pela doença. Pancreatite recorrente ocorre em 50% dos pacientes com SQF; a taxa geral de mortalidade associada é de 5 a 6%, podendo chegar a 30% em subgrupos de pacientes que evoluem com necrose pancreática ou falência persistente de múltiplos órgãos.^59^

#### 7.1.3. Manifestações Neurológicas

Fadiga, confusão mental, irritabilidade e déficits cognitivos – descritos como “ *mental fog* ” – estão entre os sintomas mais comumente descritos entre os pacientes acometidos com SQF.^57 , 58^

#### 7.1.4. Hepatosplenomegalia

A hepatosplenomegalia é um dos achados reversíveis com o tratamento e resulta do acúmulo do excesso de quilomícrons nos macrófagos do sistema reticuloendotelial na SQF.^57^

#### 7.1.5. Xantomas Eruptivos

Os xantomas correspondem a lesões cutâneas eruptivas, de coloração amarelada, geralmente com halo eritematoso e cerca de 2 a 5 mm de diâmetro. São encontrados em superfícies extensoras (cotovelos e joelhos) e nádegas. Sua prevalência é baixa (acomete de 17 a 33% dos pacientes), e nem sempre se correlacionam com a ocorrência dos episódios de pancreatite.^27^

#### 7.1.6. Lipemia Retinalis

Trata-se da aparência leitosa do sangue nos vasos retinianos ao fundo de olho, e pode ser observada em até 30% dos pacientes, correlacionando-se com níveis maiores de triglicérides.^57^

#### 7.1.7. Qualidade de Vida

O estudo IN-FOCUS, com 166 pacientes com SQF, mostrou o importante impacto da doença na qualidade de vida. As taxas de internação podem interferir nas condições sociais e possibilidades de emprego, e mais de 22% referiram depressão ou ansiedade relacionada à dor ou a episódios de pancreatite.^58^

#### 7.1.8. Escore Diagnóstico

Algumas escalas ou escores de pontuação a partir das manifestações clínicas têm sido propostas para diagnóstico de SQF; entretanto, sua validação precisa ser feita em maiores amostras de populações com HTG grave.^21^ Adicionalmente, sua aplicabilidade é questionável, visto que utilizam a presença de episódios de pancreatite prévios nos seus critérios de pontuação.^59^ Fundamentalmente, o objetivo de utilização de escores de diagnóstico consiste em rastreamento de pacientes assintomáticos e prevenção de complicações como a pancreatite aguda. A avaliação de bases de dados com maior número de pacientes com SQF e o detalhamento de formas clínicas deverão contribuir para elaboração de critérios com melhor sensibilidade e especificidade para o diagnóstico da SQF.

O escore mais utilizado é o de Moulin et al.,^21^ que utiliza como critério de seleção a presença de HTG grave (>1.000mg/dL em jejum e fora da fase aguda), e pontua quando há presença de valores elevados de triglicérides, afastadas causas secundárias, história de pancreatite, dor abdominal recorrente, falta de resposta ao tratamento usual para redução de triglicérides, além de idade de início dos sintomas ( Quadro 1 ). Esse escore foi testado em coortes de pacientes com confirmação genética de SQF e na SQM, tendo sido validado em outras coortes mostrando uma área sob a curva de 0,91. Tal posicionamento recomenda que seja utilizado na triagem para o teste genético.

**Quadro 1:** – Escore clínico para suspeição de síndrome da quilomicronemia familiar

Critério	Pontuação
TG em jejum > 1.000mg/dL em 3 dosagens não necessariamente consecutivas	+ 5
* TG em jejum pelo menos uma vez > 1.760mg/dL	+ 2
TG prévio <200mg/dL	- 5
Sem fatores secundários, exceto gestação e etinilestradiol	+ 2
História de pancreatite	+ 1
Dor abdominal recorrente sem outra causa	+ 1
Sem história de hiperlipidemia familiar combinada	+ 1
Sem resposta ao tratamento hipolipemiante (redução de TG <20%)	+ 1
Idade de início dos sintomas:	
<40 anos	+ 1
< 20 anos	+ 2
< 10 anos	+ 3
Escore SQF	
Muito provável	≥10
Improvável	≤ 9
Muito improvável	≤ 8

TG: triglicérides; SQF: síndrome da quilomicronemia familiar. *Apenas se o critério anterior (TG em jejum *>* 1.000mg/dL em 3 dosagens não necessariamente consecutivas) for sim. Adaptado de Moulin et al. Atherosclerosis 2018;275:265-272. ^*21*^

## 7.2. Diagnóstico Diferencial

### 7.2.1. Síndrome da Quilomicronemia Multifatorial

Em adultos, o principal diagnóstico diferencial da SQF é com a SQM. Anteriormente denominada hiperlipoproteinemia tipo V de Fredrickson ou HTG severa poligênica, a HTG multifatorial é um distúrbio poligênico, que inclui variantes heterozigotas raras nos cinco genes SQF ou variantes comumente associadas a hipetrigliceridemia, agravado pela presença de comorbidades ou causas secundárias de aumento dos triglicérides como diabetes não controlado, hipotireoidismo, obesidade e síndrome metabólica.^22^ Fatores dietéticos, como uso abusivo de álcool, dieta rica em gorduras, açúcares simples e outros carboidratos de elevado índice glicêmico são causas comuns de exacerbação da hipetrigliceridemia. Entre as causas ambientais, destaca-se, ainda, a utilização de certos medicamentos (glicocorticoides, estrógenos orais, diuréticos tiazídicos, betabloqueadores não cardiosseletivos, antipsicóticos de segunda geração, inibidores de protease, ciclofosfamida, sequestrantes de ácidos biliares, amiodarona, ácido retinoico, isotretinoína, sirolimo, L-asparaginase e imunossupressores, como interferon e ciclosporina) e condições fisiológicas como gestação, principalmente no terceiro trimestre.^22 , 60^ Em geral, a prevalência de SQM tende a crescer de forma linear com o aumento da prevalência das causas secundárias mais comuns (obesidade, síndrome metabólica e diabetes tipo 2). Entre os portadores da SQM, a quilomicronemia é flutuante e se manifesta em fases mais tardias da vida quando comparada à SQF. Adicionalmente, a SQM tende a apresentar melhor resposta terapêutica às modificações no estilo de vida e ao tratamento dos fatores secundários, bem como às farmacoterapias redutoras de triglicérides. A SQM caracteriza-se por um risco aumentado de pancreatite, porém menor que aquele relatado em pacientes com SQF.^22 , 28 , 60^

### 7.2.2. Lipodistrofias

Outro relevante diagnóstico diferencial da SQF são as lipodistrofias, um grupo heterogêneo de enfermidades caracterizadas pela perda seletiva de tecido adiposo e que podem cursar com HTG grave e pancreatite. As lipodistrofias podem ser herdadas ou adquiridas e, quanto à extensão do acometimento, generalizadas ou parciais, sendo as formas parciais associadas à infecção pelo HIV as mais comuns. As lipodistrofias herdadas são distúrbios raros, que podem se manifestar no nascimento ou apresentar perda de gordura em fases mais tardias da vida. Tais condições ainda são um desafio diagnóstico, principalmente as formas parciais, que devem ter sua suspeita diagnóstica considerada na presença de HTG moderada a grave associada à medida de prega cutânea da coxa <22mm em mulheres, ou menor que 10 mm em homens, e/ou casos de diabetes com necessidade de uso de insulina subcutânea em doses diárias >2UI/kg.^61 , 62^

## 7.3. Abordagem das Complicações da Síndrome da Quilomicronemia Familiar

### 7.3.1. Pancreatite Aguda

Pancreatite aguda é um evento relativamente frequente, com diferentes causas, incluindo a HTG. Identificar a causa específica é fundamental para estabelecer o tratamento e prevenir futuros episódios. Nas diversas séries, colelitíase é a principal causa, seguida por consumo de álcool e HTG (menos de 10%).^63^ Apesar de ser uma causa menos frequente, valores elevados de trigliceridemia em pacientes com pancreatite se associam com maior mortalidade e pior prognóstico.^64 , 65^ Na gestação, o estrógeno estimula a produção de VLDL hepático e reduz a remoção de triglicérides pela LPL no fígado e tecido adiposo, de modo que a HTG passa a ser a causa mais frequente de pancreatite aguda.^66^

Os episódios de pancreatite em decorrência de HTG geralmente acontecem com valores de trigliceridemia >1.000mg/dL.^67^ Esse risco, bem como a severidade, aumenta mais ainda naqueles pacientes com valores >2.000mg/dL.^68 , 69^ Isso independe de a causa básica da HTG ser primária (genética) ou secundária. Contudo, as causas genéticas geralmente cursam com valores mais elevados de trigliceridemia e, consequentemente, apresentam risco mais elevado de pancreatite.

Na classificação de Fredrickson, os tipos I (quilomícron), IV (VLDL) e V (quilomícron e VLDL) apresentam HTG, sendo que a SQF (tipo I) apresenta valores mais elevados e podem evoluir com pancreatites independentemente de fatores desencadeantes (diabetes descompensado, obesidade, uso de corticoide, estrógenos ou outras drogas que causam HTG).

O mecanismo causador da pancreatite não é totalmente conhecido, mas os triglicérides, por si só, não parecem atuar diretamente no pâncreas. O acúmulo de ácidos graxo livre nas células pancreáticas acontece na presença de lipase pancreática e desencadeia a lesão celular e a inflamação pancreática.^70^ Outro potencial mecanismo é decorrente do acúmulo de GAD (descarboxilase do ácido glutâmico). Na falta de ação da LPL e consequente acúmulo de quilomícrons, há também aumento de GAD que desencadeia inflamação mediada por TNF-alfa e IL6. Também, o próprio quilomícron pode obstruir a circulação pancreática distal e causar isquemia.

Independentemente da etiologia, a apresentação clínica da pancreatite é semelhante. Não raramente, pacientes com SQF apresentam episódios repetidos de pancreatite, e alguns referem na anamnese que não sabem quantos foram, mas que foram muitos. Isso desencadeia alterações psicológicas, comprometendo a qualidade de vida.^58^ Alguns pacientes até evitam ir para festas e reuniões, porque temem comer e desencadear a pancreatite. Crianças precisam de vigilância constante dos pais, pois, uma vez que não entendem adequadamente a doença, querem comer como os colegas que não apresentam a doença. Após apresentarem um primeiro episódio de pancreatite (muitas vezes na adolescência, após a menarca), a dor do quadro agudo e a necessidade do internamento hospitalar são fatores que motivam seguir mais rigorosamente a dieta restritiva exigida para o controle da HTG severa.

Xantomas eruptivos não são frequentes, mesmo com a HTG severa. No entanto, quando presentes em um paciente com pancreatite aguda, sugerem a HTG como causa etiológica. Superfícies extensoras de braços e pernas são os locais mais frequentes. Infiltração gordurosa de fígado e baço, levando à hepatoesplenomegalia, também pode acontecer, mas é mais inespecífica.

O diagnóstico da pancreatite aguda deve se iniciar com uma suspeita clínica (dor abdominal aguda e persistente, que se irradia para o dorso), sendo confirmada por exames laboratoriais (amilase ou lipase três vezes ou mais o limite superior de normalidade) e de imagem (ultrassonografia, tomografia ou ressonância). Pelo menos dois desses três achados devem estar presentes para confirmação diagnóstica, e isso independe da etiologia da pancreatite. Não raramente, pacientes podem apresentar dor abdominal isolada, sem alterações laboratoriais ou de imagem. Na ausência de um quadro clínico sugestivo, dosagens de lipase e amilase podem mais confundir que ajudar. Valores de triglicérides <1.000mg/dL durante o episódio clínico sugestivo de pancreatite deixam a HTG como causa improvável da pancreatite.^67^

Uma vez confirmado o diagnóstico, o tratamento deverá objetivar reduzir/eliminar a dor, bem como manter hidratação adequada e, mesmo com dieta oral suspensa, permitir nutrição adequada ao quadro agudo. A redução da trigliceridemia é fundamental para reverter o processo inflamatório e, sendo ela às custas de quilomícron, responderá mais facilmente à restrição da dieta oral. Nos casos mais severos (temperatura corporal >38,5 ou <35,0; frequência cardíaca >90bpm; frequência respiratória >20/min ou pCO2<32mmHg; leucócitos >12.000 ou <4000/mL), com necessidade de uma redução mais rápida da trigliceridemia, plasmaférese pode ser utilizada. Se uma dieta adequada não for instituída ou fator desencadeante não forem controlados, a remissão do quadro é mais difícil. Insulina estimula LPL e também pode ser utilizada em alguns casos (insulina regular 0,1 a 0,3U/kg/h). Da mesma forma, heparina também atua estimulando a LPL, mas seu uso deve ser avaliado, pois pode não trazer benefícios ao médio prazo (aumenta risco de sangramento e de liberação de componentes tóxicos dos triglicérides).^63^

Uma vez que o paciente saia do quadro agudo da pancreatite, deve-se avaliar e tratar o fator que desencadeou o processo inflamatório. Manter peso adequado, praticar exercícios regularmente e evitar medicações ou outros fatores desencadeantes de HTG ajudam a prevenir novos eventos de pancreatite.

Diferentemente de outras causas de HTG que respondem bem aos fibratos, a SQF, caracteristicamente, não apresenta redução significativa da trigliceridemia com essas medicações, não sendo utilizada com finalidade de prevenir pancreatites nesses pacientes. A apo C3 é um fator inibitório da LPL, e sua inibição com o volanesorsen (um oligonucleotídeo antissentido da apo C3), com uma aplicação por semana, reduziu significativamente (77%) a trigliceridemia e, consequentemente, a chance de pancreatite.^23^ Do ponto de vista fisiopatológico e considerando os benefícios demonstrados nos estudos clínicos, pacientes com SQF se beneficiam do uso de volanesorsen. Entretanto, aqueles pacientes com pancreatites frequentes (geralmente um ou mais episódios por ano) e com dificuldade para controlar a trigliceridemia com o tratamento usual dietético teriam maior benefício.

## 8. Diagnóstico Laboratorial da Síndrome da Quilomicronemia Familiar

O laboratório clínico tem um papel coadjuvante no diagnóstico da SQF. O aspecto do soro leitoso é o principal indicador da presença de quilomícrons e acompanha os altos níveis de triglicérides. Algumas considerações devem ser observadas para que o diagnóstico laboratorial seja eficaz no rastreamento da SQF. As fases responsáveis pelo resultado da análise laboratorial dos exames que fazem parte da investigação da SQF são: pré-analítica, analítica e pós-analítica.

### 8.1. Fase Pré-analítica (Orientações para Pacientes)

#### 8.1.1. Instruções para Coleta

O jejum deixou de ser obrigatório para o exame do perfil lipídico; no entanto, em situações como nos distúrbios no metabolismo dos triglicérides, ele se impõe para a confirmação diagnóstica da SQF. Nesses casos, o jejum deve ser de 12 horas para os adultos acima de 20 anos.^2 , 71 - 73^ Para crianças, o tempo varia de acordo com a faixa etária. Para lactentes, até 1 ano, o jejum é de 3 horas ou imediatamente antes da próxima mamada; em não lactentes, de 2 a 5 anos, o jejum é de 6 horas. E para crianças acima de 5 anos e adolescentes, o jejum é de 12 horas.

#### 8.1.2. Interferentes Pré-analíticos para Análise dos Triglicérides

O preparo para a coleta da amostra para o exame dos triglicérides para o adulto (>20 anos) consta de jejum prévio de 12 horas, em que o paciente deve estar com a sua alimentação habitual mantida; o consumo de álcool deve ser evitado nas 72 horas antes, e não deve realizar exercícios físicos extenuantes nas 24 horas anteriores.^74^

Algumas situações causam o aumento do glicerol livre no sangue, levando a uma superestimação dos níveis de triglicérides, sem o acompanhamento de turvação do soro. Nesses casos, deve ser observado se o paciente teve um dos eventos descritos na literatura: exercício físico recente, ingestão alcóolica, doença hepática aguda, diabetes descompensado, nutrição parenteral ou medicação intravenosa contendo glicerol.^74^

#### 8.1.3. Orientações para o Laboratório (Período Pré-analítico)

Na HTG, o aspecto do soro varia de turvo para leitoso. Grau I – levemente turvo; Grau 2 – turvo; Grau 3 – muito turvo; Grau 4 – leitoso. Como o aspecto do soro é subjetivo, somente após a dosagem de triglicérides e o repouso de 12 horas do soro em geladeira teremos a informação da observação visual.^74^

## 8.2. Fase Analítica

### 8.2.1. Metodologias que Avaliam os Quilomícrons

As metodologias que podem ser utilizadas para indicar a presença de quilomícrons (Q) no soro são mostradas a seguir.

***8.2.1.1. Ultracentrifugação***

É o método padrão-ouro que separa as frações de lipoproteínas de acordo com o teor de lípides e a sua densidade. Contudo, esse método apresenta limitações inerentes, entre as quais falta de disponibilidade em laboratórios clínicos, alto custo e morosidade para a realização da técnica, tornando-se inviável nos laboratórios brasileiros.

***8.2.1.2. Aspecto do Soro***

Para a observação de quilomícrons no soro leitoso, recomendamos o uso de tubo coletor de sangue total com sistema de separação de fases na centrifugação e obtenção do soro no sobrenadante.^75^ Quando não for possível, depois da centrifugação e da retirada do volume para as análises laboratoriais, fazer a transferência de 1mL de soro para um tubo de hemólise descartável transparente (cristal). O soro leitoso obtido, em qualquer situação, deverá ficar em repouso na geladeira por 12 horas para ser observada a formação de uma capa cremosa, na superfície do tubo, indicando a presença de quilomícrons que deve ser reportada no laudo do paciente.^74^

***8.2.1.3. Eletroforese de Lipoproteínas***

O teste de eletroforese de lipoproteínas, também chamado de lipidograma, pode auxiliar na confirmação de presença de quilomícrons com uma banda colorida no ponto de aplicação da amostra.^74 - 76^ Contudo, esse método de separar as frações lipídicas no soro deixou de ser utilizado na rotina clínica por ser semiquantitativo e porque as frações de colesterol foram adotadas como marcadores de risco para a doença cardiovascular (DCV), não sendo recomendado o uso dessa metodologia por este documento.

Das três metodologias mencionadas, a mais acessível em todos os laboratórios é o aspecto do soro, que é a recomendada por este documento.

***8.2.2. Metodologias que Avaliam os Triglicérides***

A metodologia para dosar os triglicérides pode ser por reação enzimática colorimétrica e ou enzimática UV. Os métodos são precisos e de baixo custo. A reação inicia com a hidrolise de triglicérides em três ácidos graxos e um glicerol.^75^ Portanto, para cada molécula de triglicérides, teremos uma molécula de glicerol que irá reagir e dar a concentração de triglicérides naquela amostra. Qualquer situação fisiológica que elevar o glicerol no soro irá superestimar os níveis de triglicérides. Está descrita uma doença genética rara, *glycerol kinase deficiency* (GKD), também chamada de pseudo-hipertrigliceridemia, que causa a hiperglicerolemia e a HTG sem observação de soro lipêmico.^77^

### 8.2.3. Interferência no Resultado dos Triglicérides

A lipemia, dependendo da sua intensidade, acarreta concentrações falsamente elevadas de triglicérides devido à associação da coloração do método e a turbidez do soro. Nesse caso, para obtenção de resultado fidedigno, será necessária uma diluição da amostra, em salina tamponada (pH 7,4) ou com o diluente da automação, que é plataforma-dependente.^75^

A diluição do soro pode seguir uma escala em relação aos valores de triglicérides e ao intervalo analítico do método. Por exemplo, se o intervalo analítico for de 8 a 885mg/dL, pode-se estipular as diluições sugeridas: diluir 1:4 (triglicérides 400 a 600), 1:6 (triglicérides 601 a 1.000), 1:10 (triglicérides 1.001 a 2.000), ou 1:20 (triglicérides ≥2.001).

FUNDAMENTAL: Mesmo após realizar a diluição, deve-se manter os resultados obtidos inseridos na faixa dinâmica; isso é indispensável para mantermos a linearidade e/ou reprodutibilidade do método em uso.

IMPORTANTE: A utilização de um branco da amostra, usando-se a amostra diluída, para descontar a turvação mesmo após diluição. Usar a diferença (delta) das leituras = amostra diluída – branco amostra diluída, multiplicando esse “delta” pela diluição utilizada, e somente após isso associá-la aos controles e/ou calibradores da plataforma.

EXEMPLO: Se o resultado do soro diluído 1:4 foi de 250mg/dL, multiplica-se por 4, e o resultado será de 1.000mg/dL de triglicérides. Porém, ao realizar o branco da amostra e obtiver 50mg/dL, subtrair esse valor do soro diluído 1:4 (250 – 50 = 200) e, multiplicando por 4, o resultado será de 800mg/dL de triglicérides. Portanto, é fundamental descontar a turvação no soro diluído. Quanto maior a diluição, maior poderá ser a superestimação de triglicérides, caso o branco da amostra não seja utilizado.

Assim, é indispensável analisarmos a descrição técnica da metodologia em uso para obtermos informações e indicações, tais como intervalo analítico (faixa dinâmica), relação da diluição a ser utilizada, material diluente, uso de branco da amostra ou mesmo alteração na programação em uso (automação). Essas descrições são método-plataforma e fabricante-dependentes, e devem ser seguidas de acordo com as suas informações.^75^

### 8.2.4. Interferência dos Triglicérides em Outros Analitos

***8.2.4.1. LDL-C***

A análise laboratorial do LDL-C é prejudicada pela elevada quantidade de triglicérides no soro lipêmico. O cálculo do LDL-C pela fórmula de Friedewald, de uso comum, além de ser limitado aos níveis de triglicérides até 400mg/dL, também pode ser subestimado, e deixa-se de tratar o paciente pela interferência de triglicérides. No entanto, a fórmula de Martin aplica fatores de correção na fórmula de Friedewald que permitem estimar com maior fidedignidade o LDL-C e pode ser aplicada com valores de triglicérides de até 13.975mg/dL. Além disso, a dosagem pela metodologia direta pode ser usada, mas irá apresentar uma limitação, a depender do grau da lipemia.^2 , 71 - 73^

Na SQF, ou na SQM, a HTG é severa pela presença de Q, VLDL e seus remanescentes. O paciente apresenta uma redução da hidrólise da lipoproteína VLDL que leva a uma diminuição da produção da lipoproteína LDL no plasma e elevada quantidade de partículas grandes e ricas em triglicérides (Q e VLDL), quando comparado no mesmo volume de amostra do indivíduo normal. Nesse caso, não importa a metodologia, o LDL-C calculado ou dosado pelo método direto, os valores sempre se apresentam inferiores à sensibilidade analítica do método. Recomendamos para os laboratórios liberarem os valores de LDL-C muito baixo ou negativos como sendo <10mg/dL.^73 , 78^

***8.2.4.2. Plaquetas***

A contagem das plaquetas em automações de hematologia é realizada com o efeito de impedância e, no caso da lipemia, a interferência possivelmente acarretará redução de sua contagem. Essa mesma associação ocorre com a determinação do hematócrito – nesse caso, com uma importante informação, e seus resultados são calculados a partir da associação entre determinação da hemoglobina e a contagem de eritrócitos.^75^

***8.2.4.3. Analitos com Avaliação Colorimétrica***

Os métodos com leituras colorimétricas de “ponto final” geralmente apresentam maiores restrições frente à lipemia. Isso também pode ocorrer, mesmo em menor intensidade, nos sistemas de leitura em UV. Tal interferência é diretamente proporcional à turbidez do soro, mas nem sempre proporcional à concentração dos triglicérides. Deve-se considerar que as lipoproteínas apresentam diferentes tamanhos e percentuais de triglicérides em sua constituição.^75^

***8.2.4.4. Enzimas***

As reações enzimáticas cinéticas, colorimétricas e/ou UV podem sofrer interferência da lipemia, dependendo da sua intensidade. Assim, temos fosfatase alcalina e a gama GT que se apresentam com maiores limitações, pois empregam em seus ensaios o p-nitrofenilfosfato (método colorimétrico). Contudo, o uso de métodos exclusivamente UV também pode ter restrições com a lipemia.^75^

***8.2.4.5. Eletrólitos***

Na determinação do sódio, no soro e/ou plasma, com valores elevados de triglicérides, o resultado será falsamente baixo. Nesse caso, pode-se usar um cálculo para correção do valor do sódio: triglicérides (g/dL) x 4 – 0,60 = fator percentual.^75^

Exemplo: Na+ 122 mmol/L e triglicérides 2.100mg/dL, teríamos: 2,10×4−0,60=7,8%


Na+122×7,8%=131,5mmol/L(Na+corrigido)

### 8.2.5. Análises Laboratoriais para Diagnóstico Diferencial

***8.2.5.1. Atividade da LPL com Heparina***

A atividade da LPL não é realizada em rotina laboratorial, mas pode ser útil em triagem para a realização do diagnóstico genético da SQF. Quando o laboratório permite que o ensaio de atividade de LPL seja realizado antes e 10 minutos após a injeção de heparina (heparina IV [50UI/kg]), o sangue total deve ser obtido do outro braço, em tubo heparinizado, e transportado em gelo úmido para o laboratório. O tubo da coleta deverá ser centrifugado durante 10 minutos, 3.000 rpm a 4^o^C, e o plasma deverá ser separado imediatamente. Armazenar o tubo com o plasma a -80^o^C até o dia da realização da análise, seguindo o protocolo adotado ou enviar para um laboratório de referência em dislipidemias.

A atividade de LPL é encontrada drasticamente diminuída em SQF pela alteração genética da LPL em homozigose, e frequentemente reduzida quando as alterações ocorrem em cofatores da LPL (APOC2, APOA5, GPIHBP1 e LMF1), em casos de homozigose ou em heterozigose composta. No entanto, foi demonstrado por pesquisadores que a capacidade discriminativa desse teste na identificação de portadores de variantes comuns nos genes LPL é limitada, o que justifica não ser recomendado neste documento.^79^

***8.2.5.2. Dosagem de Apolipoproteína C3 Plasmática***

Níveis plasmáticos elevados de apolipoproteína C3 (APOC3) são um importante fator de risco para HTG. Estudos recentes concluíram que APOC3 também inibe uma via independente de LPL de lipoproteína rica em triglicérides. A dosagem de APOC3 é viável nos laboratórios clínicos de grande porte ou nos laboratórios de apoio aos demais laboratórios.^71^

## 8.3. Fase Pós-analítica

### 8.3.1. Recomendações para as NOTAS nos Laudos Laboratoriais21,23

- Em adultos, valores de triglicérides >1.000mg/dL, avaliados após jejum de 12 horas, em três coletas diferentes e descartadas causas secundárias de HTG, o diagnóstico de hiperquilomicronemia deve ser considerado.
- Em crianças e adolescentes, valores de triglicérides >880mg/dL, independentemente do tempo de jejum, em três coletas diferentes e descartadas causas secundárias de HTG, o diagnóstico de hiperquilomicronemia deve ser considerado.
- Em crianças ou adultos, a presença de uma dosagem de triglicérides <170mg/dL EXCLUI a investigação de hiperquilomicronemia.

**Recomendação:** O adulto deve ficar em jejum de 12h, alimentação habitual, sem álcool (72 horas) e sem exercícios físicos (24 horas). Para crianças, o tempo varia de acordo com a faixa etária. Para lactentes, até 1 ano, o jejum é de 3 horas ou imediatamente antes da próxima mamada; não lactentes, de 2 a 5 anos, o jejum é de 6 horas. E para crianças acima de 5 anos e adolescentes, o jejum é de 12 horas. O excesso de glicerol livre no sangue superestima os níveis de triglicérides. O soro leitoso deve ficar na geladeira por 12 horas para verificar a presença de quilomícrons. Na dosagem dos triglicérides, observar o intervalo analítico, a relação da diluição, o material diluente e o uso de branco da amostra ou a alteração na automação. Na HTG severa, SQF ou SQM, os valores do LDL-C calculado ou dosado pelo método direto, muito baixo ou negativos, devem ser liberados como sendo <10mg/dL. A lipemia, dependendo da sua intensidade, interfere na contagem das plaquetas, nos métodos colorimétricos, nas reações enzimáticas (cinéticas, colorimétricas e/ou UV) e na determinação do sódio. A atividade da LPL com heparina não é recomendada neste documento. A dosagem de APOC3 é viável nos laboratórios clínicos. Recomendamos constar nos laudos laboratoriais que o diagnóstico de SQF, após descartadas as causas secundárias de HTG, deve ser considerado nas situações: 1) adultos em jejum de 12 horas com triglicérides >1.000mg/dL, em três coletas diferentes; 2) crianças e adolescentes, com valores de triglicérides >880mg/dL, independentemente do tempo de jejum, em três coletas diferentes; 3) em crianças e adultos, a presença de uma dosagem de triglicérides <170mg/dL EXCLUI a investigação de hiperquilomicronemia. (Grau de Recomendação I, Nível de Evidência C).

## 9. Aconselhamento Genético e as Etapas no Diagnóstico e Acompanhamento das Hipertrigliceridemias Graves

A Sociedade Americana de Genética Humana define o aconselhamento genético como um processo de comunicação que lida com problemas humanos associados com a ocorrência, o risco de ocorrência ou de recorrência de uma determinada doença genética em uma família.^80^

O termo aconselhamento genético surgiu pela primeira vez em 1947, utilizado por Sheldon Reed,^81^ como uma forma de, em um mundo pós-segunda guerra mundial, enfrentar os conceitos eugenistas que permeavam muito a sociedade científica e médica quanto às doenças genéticas e às deficiências de modo geral. A partir de então, passou a munir-se dos princípios do modelo psicossocial de abordagem ao paciente, utilizando como base a empatia e as habilidades da comunicação humana, de reconhecer o processo do luto e dos procedimentos de autodefesa. O profissional utiliza a neutralidade moral e a não diretividade – dois princípios fundamentais do Aconselhamento Genético – para orientar o paciente e a família, fornecendo respostas e informações mais completas possíveis para que o próprio consulente possa tomar suas decisões, consciente dos riscos e das alternativas.

O termo Aconselhamento, na realidade, não expõe o verdadeiro objetivo da orientação, pois a etimologia do verbo aconselhar indica “dar conselhos”, quando, na realidade, não é esse o objetivo do procedimento. O mais próximo da tradução original, *genetic counseling* ,^82^ seria *consultoria genética* : o objetivo é, como dito, orientar para que o paciente sinta segurança na tomada de decisões, entendendo que não existe certo ou errado, tão qual não deve existir uma sugestão de conduta. Dito isso, é importante entender que ao realizar o aconselhamento genético, o profissional deve respeitar os valores éticos e religiosos da família, seguindo sempre os três princípios que regem a ética médica: autonomia, beneficência e não maleficência.^83^

Vale observar que, o que muitos chamam de aconselhamento genético, é uma etapa do processo como um todo.^84 , 85^ O Aconselhamento Genético envolve, no total, cinco fases:

1. Estabelecimento e/ou confirmação do diagnóstico, que envolve a realização de anamnese, exame físico, elaboração de hipótese diagnóstica, solicitação e interpretação de exames complementares, podendo levar semanas, meses ou anos até o diagnóstico;
2. Cálculo do risco genético, uma fase mais teórica e muitas vezes fora do contato familiar, cujo objetivo é calcular o risco de ocorrência ou de recorrência de uma determinada condição de origem genética. Essa condição pode ter etiologia monogênica, cromossômica, multifatorial ou, ainda, desconhecida. Para cada situação, um risco diferente pode ser calculado, e a necessidade de conhecer a etiologia é fundamental para estabelecer o risco mais preciso possível;
3. Comunicação, a fase no qual se orienta sobre os riscos, muitas vezes envolvendo também conversas sobre opções terapêuticas e prognósticos. A combinação entre a fase 2 e a fase 3 é aquela que comumente as pessoas se referem quando usam o termo Aconselhamento Genético;
4. Decisões e Ação, fase que envolve auxiliar a família e o paciente frente às decisões tomadas na fase de comunicação, tanto em relação ao tratamento como possíveis decisões quanto a métodos contraceptivos;
5. Seguimento, representando uma fase contínua no qual o paciente ou a família são acompanhados, observando as necessidades individuais e a história natural da condição genética diagnosticada.

Dito isso, vale a pena observar que algumas etapas do aconselhamento genético envolvem condutas médicas, enquanto outras podem ser realizadas por diversos profissionais da saúde, desde que devidamente treinados nas habilidades de comunicação citadas anteriormente e nos conhecimentos de genética humana e médica.^84 , 85^

As duas fases que mais representam o aconselhamento genético, sem dúvida, são as fases de cálculo de risco genético e a de comunicação. Apesar de parecerem simples, concluir o risco de ocorrência ou de recorrência de determinada condição genética envolve um amplo conhecimento sobre as bases da genética e da hereditariedade. Falar de um risco de recorrência compatível com uma herança autossômica dominante ou autossômica recessiva parece simples quando pensamos nas Leis da Hereditariedade de Mendel, mas basta lembrarmos de alguns fatores confundidores para as leis da hereditariedade, como os conceitos de penetrância incompleta, expressividade variável, mosaicismo ou a heterogeneidade gênica. Cada um desses fatores pode dificultar o diagnóstico clínico entre formas diferentes da doença, tornando a orientação sobre o risco um desafio.

A confirmação de uma variante patogênica que explique o fenótipo pode ser fundamental para considerar o risco correto nesses casos. Também é importante observar que diferentes modos de herança, fora ao mendelismo, podem apresentar riscos mais complexos de serem calculados. Por exemplo, no risco multifatorial, devemos levar em consideração o número de afetados na família, a proximidade com o probando, além de fatores que podem variar caso a caso, como idade de início dos sintomas, gravidade e fatores ambientais envolvidos. Em um contexto de herança multifatorial, identificar esses riscos e considerar o quanto eles influenciam no risco total pode ser totalmente impossível, e, por isso, consideramos um risco de recorrência sempre aproximado ou empírico, levando em conta o conhecimento empírico e os riscos de recorrência calculados com base em estudos populacionais.^84 , 85^

Assim, fica mais nítida a compreensão de que, para falarmos sobre risco de ocorrência ou de recorrência, a definição clara entre SQF^20^ ou de SQM^36^ deve ser conhecida.

A SQF é herdada de uma maneira autossômica recessiva, ou seja, é necessário que o indivíduo tenha uma variante em homozigose ou duas variantes em heterozigose composta, ambas patogênicas ou provavelmente patogênicas, para que o indivíduo apresente o fenótipo.

Essa forma de herança autossômica recessiva, por mutação bialélica em homozigose (mesma mutação nas duas cópias) ou em heterozigose composta (uma mutação em cada cópia) está presente tanto nos casos de LPL como dos demais genes envolvidos com as formas monogênicas: *APOC2* , *APOA5* , *GPIHBP1* e LMF.^86^

Dessa forma, sabemos que os progenitores de um indivíduo com SQF terão uma variante em uma das cópias do gene afetado cada. Assim sendo, a irmandade de uma pessoa que apresenta SQF tem 25% de risco de também herdar a síndrome. Por fim, um indivíduo com SQF irá sempre passar uma das variantes para seus filhos. Caso a(o) companheira(o) também tenha uma variante no mesmo gene em questão, o risco para os filhos é de 50% a partir dessa combinação.^18^

Uma vez que foi reconhecido que o fenótipo de HTG pode também ser mais causado pela presença de variantes comuns raras ou funcionais em genes que aumentam triglicérides, configurando um modo de herança poligênico, faz-se necessário diagnóstico molecular para adequado aconselhamento genético.^86^

As chances de que outras pessoas na família apresentem também SQF vai depender da história familiar; portanto, a realização de um heredograma deve ser sempre considerada para auxiliar no cálculo do risco. Cabe aqui citar que, embora indivíduos com variante patogênica em heterozigose possam apresentar níveis elevados de triglicérides, a dosagem individualmente não deve ser usada para considerar o estado de portador, visto que indivíduos com a variante em heterozigose podem apresentar triglicérides em níveis normais, ao passo que indivíduos que não tenham a variante podem apresentar variação nos níveis de triglicérides por fatores ambientais.^86^

Apenas 1% dos casos de HTG apresenta mutações bialélicas. Por outro lado, estima-se que 14% dos pacientes com HTG sejam portadores de mutações raras em heterozigose, o que é 3 a 5 vezes maior que o da população geral. O uso de calculadoras de risco poligênico pode ser útil para identificar esses indivíduos.^20 , 38 , 87^

A indicação de testagem genética avalia pontos (p. ex., história familiar positiva, presença de um padrão de herança reconhecido na família, fatores secundários ausentes, paciente relativamente jovem e alterações bioquímicas importantes) que auxiliam na interpretação dos exames e, por conseguinte, no aconselhamento genético acerca do risco de recorrência familiar.^20 , 38 , 87^

## 10. Orientação Nutricional na Quilomicronemia em Adultos, Crianças e Adolescentes

O tratamento da SQF baseia-se no seguimento de dieta com restrição severa de gorduras,^88^ com a finalidade de prevenir a síntese de quilomícrons, partículas formadas exclusivamente no enterócito e que são responsáveis pelo transporte da gordura e colesterol de origem alimentar.^89 , 90^ Pelo fato de os portadores de SQF apresentarem mutações associadas à enzima lipoproteína lipase ou a seus cofatores, a hidrólise dos triglicérides alimentares se encontra comprometida.^21 , 91^ Por esse motivo, a dieta recomendada é bastante restrita e deve fornecer, no máximo, 10% das calorias ou 15 a 20g na forma de gorduras.^92^

Em função da gravidade da doença, os pacientes devem ser muito bem orientados quanto à importância do seguimento rigoroso das orientações, e o nutricionista deve indicar opções alimentares que contribuam com maior aderência ao tratamento. Alguns autores apontam a SQF como condição devastadora para os pacientes e frustrante para médicos e nutricionistas, no tocante ao principal alvo da terapia – o controle da HTG severa.^25^ A SQF impacta de forma adversa a qualidade de vida dos pacientes pela dificuldade em seguir dieta com restrição rigorosa, o que compromete de maneira significante o convívio social.^24^ O desconhecimento da SQF impede a compreensão sobre a seriedade da doença por amigos e familiares. O estudo IN-FOCUS,^93^ conduzido em 166 portadores de SQF de 10 países, mostrou que mais de 90% dos participantes apresentaram elevada dificuldade na aderência ao seguimento da dieta. A avaliação do banco de dados de um subgrupo (n=60) de participantes do mesmo estudo^58^ mostrou que 22% dos participantes relataram ansiedade, medo e preocupação em relação à quantidade e qualidade dos alimentos que devem ser consumidos, especialmente em situações sociais e de trabalho. Esses sintomas foram experimentados pelo menos uma vez ao mês, ou várias vezes durante a semana.

A equipe de saúde pode utilizar entrevistas motivacionais para auxiliar os conflitos internos dos portadores de SQF e promover maior aderência ao seguimento de dieta restrita em gorduras.^92^ Isso auxiliará o paciente a elaborar seu plano alimentar com auxílio do nutricionista e compreender sua responsabilidade no autogerenciamento da doença.

Embora a restrição de gorduras seja o ponto mais importante no tratamento da SQF, a dieta também deve ser isenta de açúcares de adição, como sacarose e xarope de milho,^92^ por induzirem vias lipogênicas hepáticas. A dieta também deve ser isenta de álcool, cujo consumo se associa de forma linear à concentração plasmática de triglicérides.^91^ Além disso, o monitoramento da ingestão de vitaminas lipossolúveis, minerais e ácidos graxos essenciais é recomendado, e a sua suplementação pode ser necessária.^94 , 95^ Especificamente em relação às gorduras da dieta, é fundamental considerar o tipo e o comprimento de cadeia dos ácidos graxos, uma vez que apresentam formas distintas de absorção e influenciam a concentração plasmática de triglicérides e a produção de quilomícrons.

### 10.1. Classificação e Absorção dos Ácidos Graxos

Os ácidos graxos saturados (SAT) são classificados em função do tamanho da cadeia carboxílica em curta, média ou longa, características que influenciam seu processo de absorção. Os ácidos graxos de cadeia curta são acetato (C2:0), propionato (C3:0) e butirato (C4:0), e os de cadeia média classificam-se em caproico (C6:0), caprílico (C8:0) e cáprico (C10:0). Já os ácidos graxos com mais de 12 carbonos são classificados como de cadeia longa: láurico (C12:0), mirístico (C14:0), palmítico (C16:0) e esteárico (C18:0).^96^ Os ácidos graxos de cadeia curta são provenientes da fermentação bacteriana no intestino, enquanto os de cadeia média são encontrados nos óleos de coco e de palma.^96 , 97^ A principal fonte do ácido láurico na dieta é a gordura de coco, e é encontrada em mínimas quantidades em outros alimentos. O ácido graxo mais abundante na dieta é o palmítico, cujas principais fontes são as carnes vermelhas e o óleo de palma. Por ser uma gordura estruturalmente estável, tornou-se muito utilizada nos alimentos industrializados.^98^ As principais fontes de ácido mirístico são gordura de coco, leite e derivados, enquanto o ácido esteárico tem o cacau como fonte referência.^99^ Todos os ácidos graxos insaturados apresentam cadeia longa e são classificados em monoinsaturados (MONO) ou poli-insaturados (POLI). Os principais ácidos graxos monoinsaturados são o palmitoleico (C16:1 ω7) e oleico (18:1, ω9), com apenas uma dupla ligação em sua estrutura.^100 , 101^ A principal fonte alimentar de palmitoleico é a macadâmia, enquanto o oleico é encontrado principalmente nos óleos de oliva e canola, e também em oleaginosas como amendoim, avelã, macadâmia, amêndoas e castanha-de-caju.^102^ Encontram-se presentes também nas gorduras da carne de boi, frango e porco, podendo representar entre 40% e 50% das gorduras totais desses alimentos.^103 , 104^

Os ácidos graxos poli-insaturados contêm duas ou mais duplas ligações e fazem parte das séries ômega-6 (ω6) ou ômega-3 (ω3), em função da localização da primeira dupla ligação na cadeia carbônica a partir do terminal metila. Ácidos graxos da série ω6 são representados pelo linoleico (C18:2), cujas principais fontes são óleos vegetais (girassol, milho e soja), nozes e castanhas. O ácido araquidônico (C20:4), outro representante ω6, é sintetizado endogenamente por meio de ação enzimática a partir do ácido linoleico. O ácido alfalinolênico (ALA [C18:3]), da série ω3, tem origem nos óleos vegetais, principalmente nos óleos de canola e soja, e também na linhaça e chia.^105^ Os ácidos graxos ω3 de origem animal são o eicosapentaenoico (EPA [C20:5]) e docosaexaenoico (DHA [C22:6]), encontrados nos óleos de peixes e crustáceos, principalmente nos *habitats* de águas frias e profundas.^106 - 108^ Os ácidos graxos linoleico e linolênico são considerados essenciais, pois não são sintetizados no organismo de humanos, razão pela qual devem ser providos pela dieta, e, em condições especiais de carência, recomenda-se sua suplementação.^88^

Os ácidos graxos *trans* também apresentam cadeia longa, categoria representada principalmente pelo ácido elaídico (18:1, n-9 *t* ), encontrado em gorduras vegetais, provenientes da hidrogenação parcial de óleos vegetais durante sua confecção.^109 , 110^ Os ácidos graxos *trans* são encontrados em mínimas quantidades nas carnes e leite sob a forma de ácido vacênico (18:1, n-11 *t* ), que é produzido por meio da bio-hidrogenação de gorduras sob ação da microbiota do rúmen de animais ruminantes.^109^

### 10.2. Absorção das Gorduras

As gorduras presentes nos alimentos são compostas por triglicérides (90 a 95%), fosfolípides, colesterol e vitaminas lipossolúveis. Embora o principal local para digestão das gorduras seja o intestino, esse processo inicia-se minimamente na boca, por meio da lipase lingual, seguindo ao estômago, no qual ocorre a liberação de 10 a 30% de ácidos graxos, com início do processo de emulsificação das gorduras.^89 , 111^ O processo tem continuidade no intestino, que, sob ação da lipase pancreática, induz hidrólise dos triglicérides remanescentes, liberando ácidos graxos e monoacilglicerol.^112 , 113^

O mecanismo de absorção dos ácidos graxos é complexo, pois conta com múltiplos sistemas absortivos.^114^ Os ácidos graxos de cadeia curta (acetato, propionato e butirato) são primariamente absorvidos por transporte ativo dependente ou não de sódio, por meio de transportadores monocarboxílicos. Contudo, receptores acoplados à proteína G (GPCRs), como os GPR41 e GPR43, também podem participar do processo absortivo desses ácidos graxos. Os ácidos graxos de cadeia média (cáprico, caprílico e caproico) são absorvidos predominantemente por transporte passivo, mas o GPR84 também pode participar de sua incorporação na superfície do enterócito.^115^ Após absorção, são conjugados à albumina, direcionados ao fígado, via sistema porta.^114^ Por outro lado, os ácidos graxos de cadeia longa, saturados, insaturados ou *trans* , necessitam de mecanismos mais complexos envolvidos no processo de absorção, e seu transporte no plasma depende da formação de quilomícrons.^96 , 97^ Podem ser absorvidos por difusão passiva, quando a concentração do lúmen é superior que a intracelular, ou por meio de receptores/transportadores de membrana. Por exemplo, o transportador CD36 ( *cluster of differentiation* 36) possibilita captação de ácidos graxos de cadeia longa, mesmo quando suas concentrações luminais são menores que as intracelulares.^116^ A proteína FATP4 (proteína-4 transportadora de ácidos graxos) é amplamente distribuída no intestino, sendo um dos principais transportadores de ácidos graxos de cadeia longa.^117^ No interior dos enterócitos, os AGs são transportados pelas proteínas FABP1 e 2 (proteína ligadora de ácido graxo 1 e 2)^116^ e reesterificados, retornando ao formato de triglicérides por meio da enzima diacilglicerol aciltransferase.^89^ A seguir, os triglicérides são incorporados às apolipoproteínas B48 (ApoB48) por meio da proteína microssomal de transferência de triglicérides (MTP), que dá início à formação dos quilomícrons.^118^ Os quilomícrons são processados no complexo de Golgi e, posteriormente, secretados na linfa e direcionados à circulação sistêmica, via ducto torácico.^89 , 90^

Na circulação sanguínea, os triglicérides dos quilomícrons são hidrolisados pela enzima LPL, que se encontra aderida ao endotélio dos tecidos extra-hepáticos, liberando ácidos graxos livres, posteriormente ligados à albumina, que seguem para armazenamento no tecido adiposo e minimamente no tecido muscular.^119^ Com a hidrólise dos quilomícrons, são formados os remanescentes de quilomícrons (remQM), que são removidos da circulação por meio de sua interação com receptores hepáticos do tipo B/E e LRP (proteína relacionada ao receptor de LDL).^120^

### 10.3. Tratamento Nutricional

#### 10.3.1. Gorduras

Como na SQF ocorre comprometimento da lipólise dos triglicérides pela presença de mutações na LPL ou de seus cofatores ( *GPIHBP1* , *LMF1* , *APOA5* ou *APOC2* ), os ácidos graxos de cadeia longa devem ser minimamente consumidos, com finalidade de prevenir a elevação da concentração plasmática de quilomícron.^27^ Recomenda-se, assim, o consumo de 10% do valor calórico total (VCT), na forma de gorduras da dieta.^92^ No entanto, dependendo da gravidade da doença, a restrição pode ser ainda mais severa, chegando à recomendação máxima de 5% na forma de gorduras.^91^

Além de manter limite severo com relação à quantidade total de gorduras, alimentos ricos em ácidos graxos saturados devem ser consumidos em menor quantidade. Os ácidos graxos saturados estão envolvidos em importantes vias lipogênicas hepáticas ao ativarem a proteína ligadora ao elemento responsivo ao esterol-1c (SREBP1c), que atua como fator de transcrição codificador dos genes da acetil-CoA carboxilase (ACC), ácido graxo sintase (FAS) e estearoil-CoA-dessaturase (SCD1),^121 , 122^ enzimas envolvidas na síntese de ácidos graxos, precursores para a síntese dos triglicérides, por meio da enzima diacilglicerol aciltransferase (DGAT).^123^

Importante salientar que, embora os ácidos graxos insaturados do tipo ω3 regulem a síntese de triglicérides ao bloquearem a SREBP1c, não são recomendados para o tratamento da SQF, mesmo em doses elevadas, pois os indivíduos não apresentam defeito na síntese hepática de triglicérides, mas, sim, em sua hidrólise.^27^

Por outro lado, por serem considerados essenciais, pode ser necessária a suplementação tanto do ácido alfalinolênico (ω3) como do ácido linoleico (ω6) para portadores de SQF, com a finalidade de evitar sua deficiência. O *Global Burden of Disease Study,* ^124^ estudo conduzido em 197 países, sugere consumo ideal de ω6 de 11% do VCT, embora o consumo médio global seja de 4,5% do VCT. Quanto ao ω3, o consumo ótimo indicado é de 0,25g/d, sendo o consumo médio global de 0,1g/d.^124^ Já o *Recommended Dietary Allowences* (RDA) preconiza o consumo diário de w3 entre 0,5g e 1,4g, dependendo da faixa etária.^125^

#### 10.3.2. Triglicérides de Cadeia Média

Os triglicérides de cadeia média são constituídos por ácidos graxos saturados caproico (C6:0), caprílico (C8:0) ou cáprico (C10:0), obtidos pelo fracionamento do óleo de coco ou palma e são encontrados comercialmente, juntos ou isolados. Podem apresentar pequena quantidade de ácido láurico (no máximo, 1 a 2%).^126 , 127^ O uso de TCM é permitido para indivíduos portadores de SQF, uma vez que esses ácidos graxos são absorvidos quase em sua totalidade via sistema portal, sendo minimamente incorporados aos quilomícrons.^128 , 129^ É importante reforçar a ideia de que o ácido láurico (C12:0) é considerado de cadeia longa, e seu transporte ocorre principalmente via quilomícron. Somente ocorre via porta quando excede a sua capacidade de armazenamento nessa lipoproteína.^129 , 130^ Assim, é fundamental observar com atenção a composição de ácidos graxos do produto, devendo este, preferencialmente, ser isento ou conter concentrações mínimas de láurico, a fim de evitar o incremento nas concentrações de quilomícrons.

O uso de TCM para portadores de SQF é indicado com a finalidade de contribuir com aporte calórico tanto para lactentes, crianças e adultos, sendo coadjuvante ao tratamento, mas sua tolerabilidade deve ser testada, visto que diversas pessoas relatam desconforto gastrintestinal com seu uso.^92^

#### 10.3.3. Carboidratos

Recomenda-se consumo de alimentos fontes de carboidratos complexos, ricos em fibras, como (arroz integral, feijão, ervilha, lentilha, grão de bico). A ingestão de frutas é recomendada em quantidades adequadas, sendo, no máximo, 3 a 4 porções ao dia, para não extrapolar o consumo de açúcar. Alguns autores preconizam o limite de 60% das calorias na forma de carboidratos totais.^92^ Quanto aos açucares de adição (sacarose e xarope de milho), devem ser excluídos totalmente, por induzirem aumento da síntese hepática de ácidos graxos, contribuindo à elevação da concentração plasmática de triglicérides. Os açucares são constituídos por glicose e frutose, sendo que a frutose promove intensa lipogênese hepática, não somente por servir como substrato à síntese de ácidos graxos, mas por estimular expressão de enzimas envolvidas na *de novo* lipogênese via ativação da proteína ligadora ao elemento responsivo a carboidratos (ChREBP) e esterol (SREBP1c).^131 - 133^ Além disso, o excesso de frutose diminui a betaoxidação de ácidos graxos, por induzir modificações pós-traducionais nas proteínas mitocondriais, diminuindo o número e o tamanho dessas organelas.^134^

Em virtude da intensa atividade lipogênica induzida pela frutose, sucos de frutas concentrados devem ser excluídos para portadores de SQF.

#### 10.3.4. Álcool

As bebidas alcoólicas devem ser totalmente excluídas por elevarem a concentração plasmática de triglicérides. O processo de metabolização do álcool se inicia minimamente no estômago pela ação da enzima álcool desidrogenase (ADH), mas é principalmente metabolizado no fígado, por meio de três vias: citocromo P450 2E1, catalase e ADH. Durante o processo de metabolização do álcool, ocorre formação de acetaldeído, que é convertido em acetado, pela enzima aldeído desidrogenase (ALDH), com participação principal da ADH.^135^ O acetato pode ser convertido a ácidos graxos, precursor da síntese de triglicérides.^136^

#### 10.3.5. Lactentes e Primeira Infância

A alimentação nos 2 primeiros anos de vida com quantidade e qualidade adequados de nutrientes é imprescindível para promover crescimento e desenvolvimento cognitivo adequados, além de consolidar hábitos alimentares saudáveis. No entanto, a elaboração do plano alimentar para lactentes e crianças portadoras de SQF é desafiadora para o nutricionista e para a família, a fim de garantir quantidades recomendadas de macro e micronutrientes, em função da rigorosa restrição de gorduras. A capacitação do nutricionista nessa área é de fundamental importância para harmonizar a dieta, oferecer sugestões de cardápios para a família e acompanhar de forma intensiva a implementação dos novos hábitos alimentares. A família deve estar ciente de que o consumo de gorduras além do permitido, mesmo em quantidades mínimas, pode provocar elevação indesejável da concentração plasmática de quilomícrons.

Para bebês portadores de SQF em aleitamento materno, a amamentação deve ser interrompida assim que o diagnóstico for confirmado, o que traz frustação e tristeza para o lactente e a mãe. O leite materno tem aproximadamente 3,2% de gorduras, com fração lipídica composta em aproximadamente 98% de triglicérides. A composição exata em ácidos graxos depende da alimentação materna e varia significativamente durante o período de amamentação.^137^ Os triglicérides do leite são formados predominantemente por ácidos graxos de cadeia longa saturados (35 a 40%), monoinsaturados (45 a 50%) e poli-insaturados (15%), com predomínio do palmítico, oleico e linoleico, respectivamente.^137 , 138^ Ácidos graxos insaturados com mais de 20 carbonos na cadeia, contendo duas ou mais duplas ligações, representam apenas 2% do total de ácidos graxos do leite.^138^ Ácidos graxos da série ω3 encontram-se em pequenas quantidades no leite materno: alfalinolênico (0,019g/100mL), EPA (0,003g/100mL) e DHA (0,008g/100mL).^139 , 140^

Importante estudo conduzido na Europa ( *European Childhood Obesity Project)* acompanhou 174 crianças desde o nascimento até completarem 1 ano, e contribuiu para melhor compreender o consumo calórico de lípides, carboidratos e proteínas nesse estágio de vida, cujos resultados podem ser extrapolados para orientação alimentar de crianças impossibilitadas de receber leite materno.^139^ O consumo calórico diário médio foi de 419kcal no primeiro mês, 589kcal no sexto mês e 860kcal em 12 meses. O consumo de gorduras foi de 21g/dia até os 6 primeiros meses, com aumento gradativo até 34,2g no final de 12 meses. Com relação aos ácidos graxos essenciais, até o terceiro mês com aleitamento materno praticamente exclusivo, o consumo médio diário de alfalinolênico (ω3) foi de 0,118g, e linoleico (ω6) de 2,40g. Quanto aos ácidos graxos marinhos, o consumo de EPA foi de 0,022g e de DHA foi de 0,048g.^139^ De acordo com a *Academy of Nutrition and Dietetics* , a recomendação de ácidos graxos ω3 para lactentes e crianças de 0 a 12 meses é de 0,5g e, para crianças de 1 a 3 anos, é de 0,7g/dia, enquanto para os ácidos graxos ω6, a recomendação é de 4,6g para crianças de 0 a 6 meses, e 7g/dia para a faixa de 7 a 12 anos.^125^

Em virtude da intensa restrição de gorduras na dieta, recomenda-se o monitoramento do consumo de vitaminas lipossolúveis (A, E, D e K),^92^ cuja recomendação para lactentes e crianças encontra-se disponível na tabela da Dietary Reference Intake.^95^

Em substituição ao leite materno, recomendam-se fórmulas lácteas especiais, que se assemelham parcialmente à composição nutricional do leite materno.^92^ Com relação ao teor de gorduras, as fórmulas devem ser exclusivamente preparadas com ácidos graxos de cadeia média (cáprico, caprílico e caproico), além de fornecer vitaminas lipossolúveis e quantidades permitidas de ácidos graxos essenciais. Além disso, o TCM pode ser recomendado para alcance do aporte calórico ideal, de acordo com a tolerância, uma vez que são minimamente transportados pelos quilomícrons.^128 , 129^

A inclusão de alimentos sólidos como hortaliças, frutas, carnes magras (peixe, frango sem pele, cortes magros de carne de vaca) grãos etc. deve seguir as recomendações das sociedades nacionais e internacionais de Pediatria, mantendo, no máximo, 10% das calorias na forma de gordura.

Recomenda-se consumo de líquidos em quantidades adequadas e suficientes para manutenção do balanço hídrico, que irá contribuir com a função pancreática. É sabido que desidratação prolongada induzida por vômitos e diarreia pode aumentar o risco para pancreatite associada à SQF.^92^

A orientação alimentar deve ser individualizada e agradável, respeitar hábitos culturais e ser sustentável a longo prazo. Crianças devem ser aconselhadas quanto à importância da leitura dos rótulos dos alimentos, e a família deve ser orientada quanto a preparações contendo quantidades mínimas de gordura, além de informar sobre a importância de refeições/alimentos preparados em casa.

#### 10.3.6. Gestantes

Durante a gestação, sobretudo ao final do 2º e 3º trimestre, o aumento das concentrações plasmáticas de lípides é esperado, com concentrações de triglicérides aumentadas de 2 a 4 vezes, mas bem toleradas. Nessa fase, o aumento da resistência à insulina e a ação de hormônios placentários contribuem para maior lipólise do adiposo. Além disso, há maior produção hepática de VLDL e diminuição da atividade da lipase hepática. A atividade da LPL também se encontra diminuída, o que prejudica a hidrólise de triglicérides das lipoproteínas. Em virtude dessas alterações, as lipoproteínas ricas em triglicérides têm seu *clearance* reduzido, consecutivamente, permitindo elevação nas concentrações plasmáticas de triglicérides.^141 , 142^

O aumento de triglicérides durante a gestação implica maior risco de complicações para mãe e bebê, pois eleva o risco de pancreatite aguda, podendo induzir antecipação do parto, aborto e mortalidade. Na gestação, apesar de rara, a pancreatite aguda é normalmente causada por litíase biliar. A elevação das concentrações de colesterol e a hipomotilidade da vesícula biliar ocasionada pelo perfil hormonal característico da gestação predispõem à formação de cálculos que podem vir a obstruir o ducto pancreático. Por outro lado, mulheres portadoras de SQF apresentam elevação pronunciada das concentrações de triglicérides, que pode desencadear pancreatite aguda. Gestantes portadoras de SQF apresentam elevação de 4% no risco para pancreatite aguda, com concentrações de triglicérides >1.000mg/dL, bem como aumento de 14% quando >2.600mg/dL.^94^

O tratamento dietoterápico de gestantes portadoras de SQF tem como objetivo manter as concentrações plasmáticas de triglicérides inferiores a 500mg/dL ao longo da gestação. Para isso, é necessário o seguimento de dieta, com restrição de gorduras (inferior a 20g/dia), adequação do consumo de vitaminas, minerais e ácidos graxos essenciais, de acordo com as recomendações de ingestão para o estágio de vida, incluindo monitoramento do ganho de peso. Dietas com restrição severa do consumo de gorduras devem ser constantemente monitoradas, para garantir adequação da ingestão de calorias, macro e micronutrientes, em especial os ácidos graxos essenciais^143^ e vitaminas lipossolúveis.^92^

O uso de TCM (livre de ácidos graxos de cadeia longa) pode ser indicado a fim de alcançar aporte de calorias, quando necessário. Além disso, recomenda-se salientar a importância do consumo adequado de líquidos a fim de se manter o balanço hidroeletrolítico adequado. Gestantes com SQF associada ao diabetes *mellitus* tipo 2 ou ao diabetes gestacional necessitam maior atenção na adequação da dieta, requerendo acompanhamento multidisciplinar, a fim de controlar as concentrações lipídicas e glicêmicas, bem como desenvolvimento fetal.^92^

Até o momento, tratamento dietoterápico da SQF é a única ferramenta disponível para controle das concentrações plasmáticas de triglicérides nesta condição, conforme demonstrado em recente publicação do acompanhamento de gestante que apresentava concentração plasmática de triglicérides de 8.683mg/dL e que referia episódios de pancreatites anteriores.^144^

O nutricionista deve auxiliar a paciente a elaborar o plano alimentar, fornecendo auxílio com receitas e estratégias que facilitem o seguimento da dieta. Preferências alimentares, hábitos culturais e estilo de vida devem ser considerados, bem como adequação nutricional e de calorias. A severa restrição de alimentos dificulta o seguimento da dieta; assim, é de extrema importância que haja acompanhamento por equipe multidisciplinar para que se mantenha o controle das concentrações de lípides e minimize o risco de complicações.^92^

#### 10.3.7. Recomendações Gerais

1. Restringir a ingestão de gordura na dieta (10% a 15% do VCT);
2. Exclusão de açúcares de adição (sacarose e xarope de milho);
3. Exclusão de sucos de frutas concentrados;
4. Exclusão de bebidas alcoólicas;
5. Consumo de carboidratos complexos em quantidades adequadas;
6. Garantia da adequação de ácidos graxos essenciais;
7. Monitoramento do consumo de vitaminas lipossolúveis, com suplementação quando necessário;
8. Inclusão de TCM com a finalidade de aporte calórico, de acordo com tolerância.

## 10.4. Exemplos de Cardápios

**Table:** 

Cardápio 1.500kcal	Porção	Lípides	kcal
**Café da manhã**			
Leite desnatado	200mL	0	61
Pão integral	2 fatias	1,6	120
Queijo *cottage*	1 colher de sopa	0,8	28
Papaia	½ unidade	0	46
Aveia	1 colher de sopa	1,7	40
**Lanche da manhã**			
Figo	3 unidades	0	80
**Almoço**			
Salada de folhas verdes, tomate, palmito, pepino	1 prato raso	0	10
Molho	1 colher de sopa	0	5
Arroz cozido sem óleo	3 colheres sopa	0,2	100
Feijão (50% caldo) sem óleo	1 concha	0,4	65,3
Cação em posta com molho de tomate e manjericão	200g	1,6	232
Brócolis refogados com alho e cebola	1 colher de servir	0	30
Abacaxi	1 fatia	0,1	70
**Lanche da tarde**			
Iogurte desnatado	200mL	0,6	88
Pão integral	1 fatia	1,9	60
Geleia sem açúcar	1 colher de sobremesa		30
Creme de ricota *light*	1,5 colher de sopa	2,1	44
**Jantar**			
Salada de folhas verdes, tomate, palmito, pepino		0	10
Molho	1 colher de sopa	0	5
Omelete de clara recheado com espinafre tomate e abobrinha	2 claras	0	80
Batata assada com alho, sal grosso e alecrim	1 unidade média	0	100
Banana assada com canela	1 unidade	0	70
**Ceia**			
Iogurte desnatado	200mL	0,6	88
**Total em gramas**		11,6	1462,3
**% kcal**		7,1	

**Table:** 

Cardápio 1.800kcal	Porção	Lípides	kcal
**Café da manhã**			
Leite desnatado	200mL	0	61
Pão integral	2 fatias	1,6	120
Queijo minas frescal *light*	1 fatia	2,3	50
Banana	1 unidade	0	70
Aveia em flocos	1 colher de sopa	1,7	40
**Lanche da manhã**			
Caqui	1 unidade	0,2	70
Tapioca	3 colheres de sopa	0	100
Creme de ricota *light*	1,5 colher de sopa	2,1	44
**Almoço**			
Salada de folhas verdes, tomate, palmito, pepino	1 prato raso	0	10
Molho		0	5
Arroz com legumes	4 colheres de sopa	0,3	128
Lentilha (50% caldo)	1 concha	0	93
Pescadinha mediterrânea	120g	1,3	91
Legumes assados com alecrim	1 colher de servir	0	80
Goiaba	1 unidade	0,5	60
**Lanche da tarde**			
Leite desnatado batido com papaia e maçã	200mL	0	150
Pão integral	1 fatia	1,9	60
Ricota com tomate e orégano no forno	30g	2,4	53
**Jantar**			
Salada de folhas verdes, tomate, palmito, pepino	1 prato raso	0	10
Molho	1 colher de sopa	0	5
Macarrão com molho de tomate cozido sem óleo	150g	0,7	185
Frango grelhado com alecrim	80g	2	127
Salada de frutas	150mL	0	120
**Ceia**			
Iogurte desnatado	200mL	0,6	88
**Total em gramas**		17,6	1820
**% kcal**		8,7	

**Table:** 

Cardápio gestante 1.800kcal	Porção	Lípides	kcal
**Café da manhã**			
1 copo de leite desnatado	250mL	0	75
Pão integral *light*	2 fatias	0,5	96
Queijo minas frescal *ligjt*	1 fatia	2,3	50
Banana	1 unidade	0	70
Farelo de aveia	1 colher de sopa	1,1	49
**Lanche da manhã**			
Iogurte desnatado	200mL	0	88
Morango	10 unidades	0,3	30
**Almoço**			
Salada de folhas verdes, tomate, palmito, pepino	1 prato raso	0	10
Molho	2 colheres de sopa	0	5
Arroz integral cozido sem óleo	4 colheres de sopa	0,6	120
Feijão (50% caldo) sem óleo	1 concha	0	50
Pescadinha ao molho de maracujá	150g	1,7	114
Legumes assados com alecrim	1 colheres de servir	1	120
Melão	2 fatias	0	60
**Lanche da tarde**			
Leite desnatado batido com	250mL	0	75
Frutas vermelhas	50g		28
Ricota	30g	2,4	53
Tapioca	2 colheres d sopa	0	74
Geleia sem açúcar	1 colher de sobremesa	0	30
**Jantar**			
Salada de folhas verdes, tomate, palmito, pepino	1 prato raso	0	10
Molho	2 colheres de sopa	0	5
Macarrão com molho de tomate cozido sem óleo	150g	0,7	185
Atum no forno com ervas	150g	1,1	180
Papaia	½ unidade	0	46
**Ceia**			
Iogurte desnatado	200mL	0,6	88
Aveia em flocos	1 colher de sopa	1,7	40
**Total em gramas**		14	1.751
**% kcal**		7,2	

**Table:** 

Cardápio infantil 1.400kcal	Porção	Lípides	kcal
**Café da manhã**			
Mingau de aveia com banana			
1 copo de leite desnatado + vitaminas A+D+E	200mL	0	67
Banana	1 unidade	0	70
Farelo de aveia	1 colher de sopa	1,1	49
Pão integral	1 fatia	0,8	60
Creme de ricota *light*	15g	1	22
**Lanche da manhã**			
Iogurte desnatado batido com	200mL	0	99
papaia e com	1/2 unidade	0	46
Leite desnatado em pó enriquecido com vitaminas A+D+E	1 colher de sopa	0	50
**Almoço**			
Salada de folhas verdes, tomate, palmito, milho e ervilha	1 prato de sobremesa	0	28
Molho	1 colher de sopa	0	5
Arroz integral	3 colheres de sopa	0,6	75
Feijão (50% caldo)	3 colheres de sopa	0	50
Peito de frango desfiado com molho de tomate	4 colheres de sopa	2	127
Abobrinha refogada	3 colheres de sopa	0	30
Melão	1 fatia	0	28
**Lanche da tarde**			
Tapioca com	30	0	108
Queijo minas frescal *light*	30	2,3	50
Tomate e orégano	0	0	0
Suco de maracujá sem açúcar	0	0	0
**Jantar**			
Salada de folhas verdes, tomate, palmito, pepino		0	0
Molho		0	5
Arroz com legumes	2 colheres de sopa	0,15	75
Lentilha	3 colheres de sopa		45
Peito de peru com alecrim	70	2,5	130
Tâmara	2 unidades	0	70
**Ceia**			
Leite desnatado morno (enriquecido com vitaminas A+D+E) com cravo e canela	200mL	0	67
Cacau em pó	1 colher de sopa	1,4	26
**Total em gramas**		11,85	1382
**% kcal**		7,7	

**Alimentos com baixo teor de gordura (< 5g por porção)**

**Table:** 

	**EM 100g**			**Por porção**		
**qtd de gordura**	**gordura**	**ω6 g**	**ω3 g**	**retinol (ug)**	**g alimento**	**g de gordura**
**PEIXES**						
filé de abadejo cru	0,4	tr	nd	tr	120	0,5
cação em posta cru	0,67	tr	nd	6	120	0,8
atum cru	0,9	0,01	0,01	20	120	1,1
pescadinha crua	1,1	0,01	0,01	tr	120	1,3
pintado cru, sem pele	1,3	0,02	0,02	tr	120	1,6
corvina crua	1,6	0,01	0,02	65	120	1,9
merluza crua	2	0,03	0,05	tr	120	2,4
filé de linguado	3	nd	nd	nd	120	3,6
filé de pescada cru	4	0,03	0,04	48	120	4,8
	**EM 100g**			**Por porção**		
**CARNES**	**gordura**	**ω6 g**	**ω3 g**	**retinol (ug)**	**g alimento**	**g de gordura**
maminha grelhada	2,4	0,19	0,04	nd	80	1,9
peito de frango sem pele grelhado	2,5	0,31	0,01	tr	80	2,0
lagarto cru	5,2	0,06	0,01	3	80	4,2
patinho sem gordura grelhado	7,3	0,17	0,03	tr	80	5,8
lagarto cozido	9,1	0,12	0,02	2	80	7,3
paleta sem gordura cozida	7,4	0,16	0,01	tr	80	5,9
coxa de frango sem pele cozida	5,8	1,1	0,05	tr	80	4,6
músculo sem gordura cozido	6,7	0,09	0,03	2	80	5,4
	**EM 100g**			**Por porção**		
**LÁCTEOS**	**gordura**	**ω6 g**	**ω3 g**	**retinol (ug)**	**g alimento**	**g de gordura**
Iogurte desnatado	0,3	tr	nd	nd	200	0,6
leite desnatado	0	nd	nd	tr	200	0,0
queijo cottage light	0	nd	nd	nd	30	0,0
queijo cottage	2,6	nd	nd	nd	30	0,8
creme de ricota light	7,1	nd	nd	nd	30	2,1
ricota	8,1	0,14	0,02	53	30	2,4
requeijão light	13	nd	nd	nd	20	2,6
queijo minas frescal light	7,7	nd	nd	nd	30	2,3
creme de queijo minas light	14,7	nd	nd	nd	15	2,2
queijo muçarela light	17	nd	nd	nd	30	5,1
queijo muçarela	25,2	0,31	0,08	109	30	7,6
queijo minas frescal	20,2	0,28	0,06	161	30	6,1
	**EM 100g**			**Por porção**		
**OVOS**	**gordura**	**ω6 g**	**ω3 g**	**retinol(ug)**	**g alimento**	**g de gordura**
clara de ovo cozida	0,1	0	0	0	3,2	0,0
ovo inteiro de galinha	9,5	0,94	0,02	32	50	4,8
	**EM 100g**			**Por porção**		
**PÃES E MASSAS**	**gordura**	**ω6 g**	**ω3 g**	**retinol(ug)**	**g alimento**	**g de gordura**
tapioca	0	0	0	0	45	0,0
espaguete cozido	0,9	nd	nd	nd	100	0,9
macarrão trigo cru	1,3	nd	nd	nd	50	0,7
pão integral com fibras	3,31	nd	nd	nd	25	0,8
pão integral light	1	nd	nd	nd	50	0,5
	**EM 100g**			**Por porção**		
**CEREAIS**	**gordura**	**ω6 g**	**ω3 g**	**retinol(ug)**	**g alimento**	**g de gordura**
quinoa flocos						0,0
arroz cozido sem óleo	0,41	0,31	0,01	nd	60	0,2
arroz integral cozido sem óleo	0,2	0,06	nd	nd	60	0,1
milho de pipoca	4	nd	nd	nd	16	0,6
aveia em farelo	7	nd	nd	nd	20	1,4
aveia em flocos crua	8,5	2,95	0,06	nd	20	1,7
linhaça	32,3	5,42	19,81	nd	10	3,2
chia	34,4	17,36	62,02	nd	10	3,4
	**EM 100g**			**Por porção**		
**LEGUMINOSAS**	**gordura**	**ω6 g**	**ω3 g**	**retinol(ug)**	**g alimento**	**g de gordura**
grão-de-bico cozido	2,6	2,71	0,13	nd	65	1,7
lentilha cozida	0,5	0,21	0,04	nd	50	0,3
feijão cozido com caldo	0,5	0,16	0,14	nd	86	0,4
cacau em pó sem açúcar	13,5	nd	nd	nd	10	1,4
**frutas proibidas**	**gordura por porção**					
pequi	18				60	10,8
abacate	8,4				100	8,4
coco	4				30	12,6
açaí	3,9				250	9,8

*nd: não disponível; tr: traços; lípides (g) por porção. 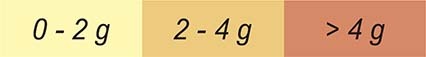
*

## 11. Aférese

Valores de triglicérides >1.000mg/dL aumentam o risco de pancreatite nos pacientes com SQF. Classe IIA, Nível C.

Nos pacientes com SQF, a pancreatite aguda (PA) á a complicação mais frequente, com prevalência de 60 a 88%.^145^ Valores de triglicérides plasmáticos >1.000mg/dL aumentam, em muito, o risco, ou podem ser indicativos da presença de pancreatite hipertriglicridêmica (PH).

A mortalidade da PA nesses pacientes é em torno de 6%, podendo chegar até 30%, conforme a presença de complicações.^146 , 147^ Estudos de coorte têm demonstrado evolução mais grave desses pacientes, com prevalência maior de complicações (choque, insuficiência renal, respiratória, sepse), quando comparados a outras etiologias de PA.^148 , 149^

### 11.1. Diagnóstico e Tratamento

Os procedimentos diagnósticos e terapêuticos iniciais da PH devem seguir as mesmas recomendações de práticas estabelecidas para quadros de PA em geral (incluindo hidratação intravenosa intensiva, tratamento analgésico e jejum). A determinação mais precoce possível dos níveis séricos de triglicérides é crucial, pois esses podem diminuir seus níveis séricos nas primeiras 48 horas, após o início da pancreatite, pelo jejum inicialmente estabelecido.^150^

Nos pacientes com SQF, a PH pode ocorrer espontaneamente, sem causa aparente, ou ser desencadeada por fatores secundários que incluem: diabetes *mellitus* não controlado, uso abusivo de álcool, gravidez e medicamentos (estrogênios orais, tamoxifeno, propofol, ácido valproico, isotretinoína, clomifeno, betabloqueadores, inibidores de protease e mirtazapina).^68^

### 11.2. Tratamento Não Medicamentoso

A base da terapia inicial da pancreatite aguda consiste em internação em unidade de terapia intensiva (UTI), bem como a restrição da ingestão oral, de fluidos intravenosos e analgesia.

A evolução clínica é dependente da redução dos triglicérides plasmáticos nas primeiras 24 a 48 horas.

A maioria dos pacientes com PH apresenta curso clínico não complicado, com bom prognóstico. Em geral, os níveis séricos de triglicérides diminuem nas 24 a 48 horas e alcançam valores <500mg/dL, no quarto ou quinto dia, apenas com medidas de suporte.^151^ Uma vez que a dor desapareça e o trânsito intestinal esteja estabelecido, uma dieta oral sem gordura pode ser reiniciada.

### 11.3. Tratamento Farmacológico

**A infusão de heparina endovenosa na PH nos pacientes com SQF não é recomendada. Classe de recomendação: III, Nível de evidência: C.**

As infusões de heparina e insulina têm sido usadas como terapia principal para PH. A maioria das evidências para ambas as drogas vem de casos isolados ou séries de casos.^152 - 156^

A infusão de heparina não fracionada é capaz de liberar a lipase lipoproteica (LLP) ligada às células endoteliais, com redução transitória dos triglicérides séricos. Nos quadros de PH grave, a infusão de heparina endovenosa a longo prazo pode esgotar a LLP da superfície das células endoteliais, permitindo que os níveis de triglicérides séricos se elevem novamente.^157 - 159^ Além disso, alguns autores relutam em usar heparina endovenosa em pacientes com necrose pancreática, pelo risco de transformação hemorrágica.^151^

**O uso de heparina de baixo peso molecular está indicado como profilaxia para trombose venosa profunda na PH nos pacientes com SQF. Classe de recomendação: IIa, Nível de evidência: C.**

Não há contraindicação para o uso de heparina de baixo peso molecular^160^ como profilaxia para trombose venosa profunda na PH.

**A insulina endovenosa deve ser utilizada apenas em pacientes com diabetes tipo 1 e 2 descompensado, para controle glicêmico, na PH nos pacientes com SQF** . **Classe de recomendação: IIa, Nível de evidência: C.**

A insulina aumenta a atividade da LLP e ajuda a reverter os efeitos hepáticos da resistência à insulina. A infusão de insulina é especialmente útil em pacientes com diabetes não controlado e hiperglicemia, além da HTG. Não há evidência clara do benefício de insulina nos pacientes com PH, que não sejam diabéticos.^19^

A terapêutica com insulina intravenosa é obrigatória para pacientes com diabetes tipo 1 descompensado e HTG grave com PH.^160 - 162^ A insulina endovenosa deve ser usada em pacientes com HTG grave e PH, com diabetes tipo 2 descompensado.^163 - 165^

### 11.4. Aférese

**A plasmaférese deve ser indicada para pacientes com PH nos portadores de SQF de forma individualizada. Os candidatos potenciais seriam os pacientes que apresentam PH grave, ou que persistem com valores de triglicérides >1.000mg/dL, após as primeiras 24 a 48 horas. Classe de recomendação: IIb, Nível de evidência: C.**

Relatos e séries de casos têm demonstrado eficácia da plasmaférese na remoção dos triglicérides da circulação dos pacientes com PH, com redução média dos valores de triglicérides entre 65 e 85% após uma ou duas sessões.^166 - 170^

Pelo fato de a PH ser uma condição com risco de vida, alguns centros utilizam a plasmaférese como procedimento de escolha na redução rápida dos quilomícrons circulantes, tão logo seja estabelecido o diagnóstico, removendo assim o agente causador do dano pancreático. A utilização desse procedimento precocemente na redução dos triglicérides plasmáticos preveniria a geração e o acúmulo de ácidos graxos livres, diminuindo seus efeitos locais e sistêmicos.^171 - 172^ O mecanismo da pancreatite aguda induzida por HTG é provavelmente causado pelo excesso de triglicérides, que, ao sofrerem hidrólise pela lipase pancreática, extravasam das células acinares para o leito vascular do pâncreas, resultando em acúmulo de ácidos graxos livres e lisolecitina. Os ácidos graxos livres são tóxicos e podem causar danos às células acinares e ao endotélio capilar.^173^ Além disso, as concentrações elevadas de quilomícrons aumentam a viscosidade sanguínea das veias com prejuízo do fluxo sanguíneo local, resultando em isquemia pancreática e piora da lesão tecidual.^174^ Os ácidos graxos livres ativam o tripsinogênio que inicia o edema local e a pancreatite necrotizante.^173^ Em série de casos publicada em hospital terciário da Turquia, com 33 pacientes admitidos com pancreatite aguda relacionada à HTG, foi demonstrada redução média de triglicérides de 54,4% após uma única sessão. Após uma segunda sessão, houve redução de triglicérides de 79,4%. Durante a evolução, 13 pacientes apresentaram coleção pancreática; 1 paciente com pancreatite necrotizante, não sendo observados casos de pseudocisto. A mortalidade nos pacientes com PH grave foi de 33,3%, e a mortalidade geral foi de 3%, sem casos relacionados ao procedimento de plasmaférese. Esse estudo demonstrou que a plasmaférese é um tratamento seguro e eficaz para pacientes com PH. São necessários mais estudos que comparem aférese + tratamento conservador e apenas tratamento conservador nos pacientes com PH.^175^

Chen et al.^167^ analisaram retrospectivamente os resultados clínicos em pacientes com PH antes (n = 34) e após (n = 60) a disponibilidade de aférese na instituição. Esses grupos apresentavam características clínicas semelhantes. Nos 20 pacientes do último grupo, foi optado por plasmaférese, com tempo médio de 3 dias para a realização do procedimento, após os sintomas iniciais. Não houve diferenças significativas quanto à mortalidade e complicações entre os pacientes submetidos ou não à plasmaférese. As limitações desse estudo se devem ao seu desenho retrospectivo, experiência de um único centro e pequeno tamanho da amostra.^167^

Alguns centros realizam plasmaférese na admissão, pouco antes de 24 horas, enquanto outros incluem pacientes entre 24 e 72 horas da admissão. Estudos têm enfatizado a importância do início precoce da plasmaférese na PH, enquanto outros não detectaram qualquer diferença na morbidade ou mortalidade relativa a um início precoce ou tardio do procedimento.^167^

Um benefício claro da plasmaférese na redução da gravidade de pacientes com PH ainda não foi demonstrado de forma conclusiva.

A plasmaférese não é isenta de riscos, além de ser um procedimento com custo elevado. Requer acesso intravenoso central e anticoagulação transitória com complicações associadas que incluem bacteremia, trombose venosa e sangramento. Os candidatos potenciais seriam os que apresentam PH grave, ou os que continuam com níveis persistentes de triglicérides maiores que 1.000mg/dL após as primeiras 24 a 48 horas.^175^

Pela falta de evidências, as recomendações para o procedimento de plasmaférese em adultos com PH nos portadores de SQF devem ser individualizadas.

Nas recentes diretrizes da American Society for Apheresis (ASFA), a recomendação de plasmaférese em pacientes com PH é de 2C (recomendação fraca) com nível de evidência III.^176^

### 11.5. Gestação e PH nos Pacientes com Síndrome da Quilomicronemia Familiar

**A indicação de plasmaférese na gestação, apesar de segura e eficaz, deve ser individualizada, pela escassez de evidências até o momento. Classe de recomendação: IIb, Nível de evidência: C.**

A gravidez normal é caracterizada por alterações adaptativas do metabolismo lipídico, destinadas a garantir as necessidades da placenta e as necessidades de glicose e lipídios para o crescimento fetal, incluindo aumento da produção de glicose, síntese de progesterona, lipogênese e redução da lipólise.^177 , 178^ Pacientes com alterações do metabolismo lipídico geneticamente determinadas, caracterizadas por redução da lipólise intravascular, podem evoluir durante a gestação com HTG grave e pancreatite.^179^

O quadro de PH aparece no terceiro trimestre da gestação ou no início do período pós-parto, com impacto elevado na morbidade e mortalidade materno-fetal.^180^

Já foram descritas taxas de mortalidade materna consequente a quadros de PH de 37% e de mortalidade fetal de 60%, mas, atualmente, esses números estão em declínio pelos avanços diagnósticos e terapêuticos.^181 - 183^

Quadros de pancreatite associada à gravidez podem ocorrer no contexto de doença do cálculo biliar, uso abusivo de álcool e HTG.^146^ Nos casos de PH, o escore de gravidade e o pior prognóstico são mais prevalentes que as outras etiologias de PA.^64 , 184^

Relatos de casos clínicos têm demonstrado que o uso de plasmaférese em gestantes tem se mostrado eficaz e seguro.^185 - 188^ Pela escassez de evidências, a indicação de plasmaféres na gestação complicada com PH, em pacientes com SQF, deve ser individualizada.

## 12. Novas Terapêuticas para o Tratamento da Síndrome da Quilomicronemia Familiar

Os tratamentos disponíveis para tratamento da SQF, visando à redução da trigliceridemia, não são efetivos para controle da quilomicronemia nesses pacientes.^24^ O uso de terapia gênica com AAV1-LPL(S447X) utilizando um vírus adenoassociado foi testado na SQF (Glybera, alipogene tiparvovec), visando expressar a LPL(S447X). No entanto, a despeito de resultados promissores, seu uso comercial não foi possível devido ao elevado custo.^189^ Assim, a única terapêutica que reduz os triglicérides <880mg/dL, ou 10mmol/L, nesses pacientes, e que parece reduzir o risco de pancreatite, é a dieta com grande restrição de gordura associada à restrição de álcool e certas medicações.^92^ A adesão ao longo da vida a essas restrições é difícil, e episódios de quilomicronemia, dores abdominais e pancreatites recorrentes são comuns. Assim, terapias adicionais são necessárias para manter os triglicérides <880mg/dL.

### 12.1. ApoC3

A APOC3 é uma glicoproteína que consiste em 79 aminoácidos, sintetizada principalmente no fígado e, em menor proporção, no intestino, e está associada às lipoproteínas contendo ApoB, incluindo os quilomícrons e as VLDLs ( *very-low density lipoprotein* ), bem como as HDLs ( *high-density lipoprotein* ).^190 - 192^ Em estudos genéticos, pré-clínicos e estudos de fase 1, a APOC3 surgiu como um regulador das concentrações plasmáticas de triglicérides.^192^ A APOC3 é um inibidor da atividade da LPL,^190^ sendo um potente inibidor da ativação da LPL, que é mediada pela APOC2, resultando em inibição da lipólise de lipoproteínas ricas em triglicérides.^190^ A APOC3 inibe a atividade da lipase hepática, para promover a montagem e a secreção da VLDL^193^ e inibir o clareamento das lipoproteínas remanescentes ricas em triglicérides.^194^ Contudo, a importância desses mecanismos independentes da LPL não é bem compreendida.

#### 12.1.1 Antissentido Anti-APOC3

A volanesorsena é uma droga antissentido de segunda geração, inibidora da síntese da APOC3 modificado. O ISIS 304801 contém uma terminação 2′-O-(2- metoxietil).^192^ A inibição da síntese da APOC3 no fígado ocorre por uma ligação sequência-específica do ISIS 304801 ao mRNA da *APOC3* , o qual, por sua vez, leva à degradação do mRNA da *APOC3* por uma RNase H1, uma ribonuclease endógena expressa em células de mamíferos.^191^ Em estudos de fase 1 com voluntários saudáveis, o ISIS 304801 promoveu redução dose-dependente e prolongada das concentrações plasmáticas de APOC3 com concomitante redução dos triglicérides.^192^ Em estudos de fase 2, o ISIS 304801 foi efetivo na redução de triglicérides em pacientes com VLDL elevado por uma série de condições.^195^

Como os pacientes com SQF apresentam atividade da LPL muito baixa e por ser um modo de ação da APOC3, a inibição da lipólise pela via LPL-dependente, seria predito que o ISIS 304801 seria inefetivo na redução dos triglicérides ou tivessem um efeito mínimo na redução dos triglicérides em portadores dessa síndrome. No entanto, deve existir um mecanismo de escape independente da via da LPL para a sobrevivência desses pacientes. Estudos pré-clínicos sugerem que a APOC3 modula os níveis de triglicérides por uma via independente da LPL. Para isso, foi feito um estudo com o ISIS 304801 em pacientes com SQF e triglicérides de 1.406 a 2.083mg/dL. Após 13 semanas de tratamento com 300mg de volanesorsena, as concentrações plasmáticas de APOC3 foram reduzidas de 71 a 90% e os triglicérides, de 56 a 86%. Durante o tratamento, todos os pacientes apresentaram triglicérides <500mg/dL. Os dados iniciais mostraram o papel da APOC3 como um regulador na via LPL-independente no metabolismo dos triglicérides.^191^

Esses dados foram replicados no estudo clínico Approach,^57^ um estudo de fase 3 duplo-cego, randomizado, com duração de 52 semanas, que avaliou a eficácia e a segurança de volanesorsena em 66 pacientes com SQF. Os pacientes foram randomizados em uma proporção de 1:1 para receber volanesorsena ou placebo. O desfecho primário foi a variação percentual dos triglicérides em jejum do período basal aos 3 meses (na semana 12 ou na semana 13). Nove desfechos secundários foram priorizados e analisados em ordem hierárquica. Se a análise do primeiro desfecho fosse significante, o segundo desfecho seria analisado quanto à significância em ordem hierárquica, e assim por diante. Se, na sequência hierárquica, um desfecho não fosse significante, os seguintes teriam análise exploratória. As variações percentuais entre o período basal e os 6 e os 12 meses foram comparadas entre os tratamentos por ANCOVA.

Foram selecionados 130 pacientes e, destes, 67 foram randomizados, e 1 no grupo placebo retirou o consentimento. Dos 66 pacientes randomizados, 41 eram homozigotos ou heterozigotos compostos para uma das 25 mutações inativadoras do gene *LPL* , e 11 pacientes apresentavam mutações bialélicas em proteínas acessórias ou eram heterozigotos duplos para mutações nos genes *LPL* e *APOA5* ou *LMF1* ; 14 pacientes não apresentavam mutações definidas, mas foram incluídos com base no seu fenótipo e na baixa atividade da LPL.^23^

Os pacientes incluídos tinham entre 20 e 75 anos, 80% brancos, 55% mulheres, e o índice de massa corporal foi de 25,0 ± 5,7, O diagnóstico de SQF foi feito com idades entre 1 e 75 anos. Lipemia *retinalis* estava presente em 21%, e xantomas eruptivos em 23; 76% tinham história de pancreatite documentada e, destes, 23 pacientes tiveram 53 episódios adjudicados nos 5 anos anteriores. Sete pacientes tinham pancreatite crônica. No período basal, 53% usavam fibratos, ácidos graxos ômega-3 ou ambos, e 20% recebiam estatinas. Sete pacientes tinham sido tratados previamente com alipogene tiparvovec (Glybera) mais de 2 anos antes da inclusão no estudo. Os valores basais de triglicérides eram elevados e não diferiram entre o grupo recebendo a medicação e o placebo (2.209±1.199mg/dL), bem como os quilomícrons de VLDL (1.849±1.176mg/dL) e APOB48 (10,2±6,6mg/dL). A APOC3 era elevada (30,2 ± 14,2mg/dL). O tratamento com volanesorsena reduziu os níveis médios de APOC3 em 84% do basal aos 3 meses, e em 83% ao sexto mês (p<0,001 para ambas comparações), com correspondente redução de 25,7 e 25,6mg/dL, respectivamente. Houve aumento de APOC3 de 6,1% (1,9mg/dL) após 3 meses e redução de 5,2% (1,7mg/dL) após 6 meses entre os pacientes que receberam placebo. O desfecho primário de eficácia, ou seja a variação percentual dos triglicérides entre o período basal e 3 meses, foi a redução de 77% no grupo volanesorsena *versus* aumento de 18% no grupo placebo (p<0,001), o que correspondeu à redução de 1.712mg/dL, intervalo de confiança – IC 95% 1.330 a 2.094mg/dL no grupo recebendo a volanesorsena, comparado a um aumento de 92,0mg/dL (IC 95%: 301 a 486mg/dL) no grupo placebo (p<0,001). Foram significantes os resultados da análise do primeiro desfecho secundário, ou seja, a taxa de resposta, definidos como alcance de triglicérides em jejum <750mg/dL aos 3 meses. No grupo volanesorsena, 77% dos pacientes, em comparação a 10% dos pacientes que receberam placebo, alcançaram níveis de triglicérides <750mg/dL ( *odds ratio* , OR 186,16; IC 95%, 12,86 a um valor que não pode ser estimado; p<0,001). O desfecho secundário subsequente, variação percentual dos triglicérides em jejum do basal aos 6 meses, também foi significante, com redução de 53% nos triglicérides correspondendo a 1.380mg/dL, comparado ao placebo com aumento de 25% ou 224mg/dL. A diferença entre os grupos foi de −77,8% (IC 95%, −106,4 a −49,1; p<0,001). A análise do terceiro desfecho secundário, variação percentual dos triglicérides em jejum do basal aos 12 meses, foi significante, e a volanesorsena reduziu triglicérides em 40% (986mg/dL) e o placebo teve aumento de 9%, ou 39mg/dL, com uma diferença entre os grupos de −49,1% (IC 95%, −94,7 a −3,5; p=0,03). O desfecho subsequente, a média da máxima intensidade das dores abdominais autorreferidas pelos pacientes durante o tratamento na análise hierárquica, não foi significante.^23^

Entre os pacientes do grupo recebendo volanesorsena, 19 completaram as 52 semanas de tratamento. Seis receberam 300mg por semana por todo o período de tratamento; entre os 13 restantes, a frequência das doses foi reduzida para 300mg a cada 2 semanas, foi pausada, ou ambas. Entre os pacientes que não tiveram redução de dose, a redução de triglicérides do basal ao terceiro mês foi de 79%, de 80% aos 6 meses e 72% aos 12 meses (reduções absolutas com relação ao basal de 1.670mg/dL, 1.656mg/dL e 1.454mg/dL, respectivamente, enquanto as reduções absolutas dos triglicérides entre os 13 pacientes que tiveram redução de dose foram de 71% aos 3 meses, 52% aos 6 meses, e 54% aos 12 meses (redução média do basal de 1.933mg/dL, 1.564mg/dL e 1.400mg/dL, respectivamente). Entre os seis pacientes cujas doses não foram reduzidas, 5 alcançaram triglicérides <750mg/dL aos 6 meses, e 4 alcançaram níveis de triglicérides <750mg/dL aos 12 meses. Dos 13 pacientes que tiveram redução de doses, 6 alcançaram valores de triglicérides <750mg/dL aos 6 meses e 6 alcançaram triglicérides <750mg/dL aos 12 meses; 3 pacientes alcançaram triglicérides <750mg/dL aos 6 e aos 12 meses.^23^

Em análise exploratória, os pacientes que receberam volanesorsena reduziram os triglicérides dos quilomícrons em 83%, APOB48 em 76%, não HDL-c em 46%, e VLDL-c em 58%, e aumento de HDL-c em 46%, APOA1 em 14%, LDL-c em 136%, e APOB em 20%.^23^

A volanesorsena reduziu os triglicérides independentemente do diagnóstico genético ou do tipo de mutação. Aos 3 meses, os valores médios de triglicérides foram reduzidos em 65% em 17 pacientes com mutações bialélicas no gene *LPL* , e em 75% nos 9 pacientes com defeitos genéticos não relacionados à *LPL* . Pacientes com mutações nos genes *APOC2, GPIHBP1, APOA5* e *LMF1* , todos, mostraram redução de triglicérides de 69 a 88%. O tratamento foi efetivo independentemente dos valores basais de triglicérides, e foi igualmente efetivo nos pacientes recebendo terapia concomitante com fibratos, ácidos graxos ômega-3 ou ambos, e os pacientes não recebendo essas terapias (redução média do basal aos 3 meses de 76 e 73%, respectivamente).^23^

Devido ao tamanho do estudo, por tratar-se de doença rara, a mudança no número de episódios de pancreatite não foi um desfecho pré-especificado. No entanto, foi feita análise exploratória dos episódios de pancreatite adjudicados que ocorreram durante o estudo. No período de tratamento, três pacientes no grupo placebo tiveram quatro episódios de pancreatite, enquanto um paciente no grupo volanesorsena teve um episódio 9 dias após ter recebido a última dose.^23^

Os efeitos adversos mais comuns durante o período de tratamento foram as reações no local da injeção e plaquetopenia.

No grupo volanesorsena, 20 (61%) pacientes tiveram pelo menos uma reação no local da injeção, em grau leve a moderado e, em média, 12% das aplicações de volanesorsena versus zero% das injeções de placebo foram associadas com essas reações. Um paciente foi retirado do estudo devido à reação no local da injeção. Plaquetopenia confirmada <140.000 por microlitro foi observada em 25 (76%) pacientes no grupo recebendo a volanesorsena e em 8 (24%) pacientes no grupo placebo; plaquetopenia confirmada <100.000 por microlitro foi observada em 16 pacientes (48%) que receberam volanesorsena, mas em nenhum dos que receberam placebo. Como não havia história documentada de plaquetopenia importante em humanos tratados com essa classe de drogas antissentido,^20^ o protocolo inicial requeria monitoramento da contagem de plaquetas a intervalos de 4 a 6 semanas. Entretanto, durante o estudo, trombocitopenia grau 4 (<25.000 plaquetas por microlitro) foi observada em dois pacientes no grupo volanesorsena, e o tratamento foi descontinuado. Não houve sangramentos maiores em nenhum desses pacientes, e ambos alcançaram valores normais de plaquetas em 23 e 33 dias após descontinuação do fármaco. Um paciente recebeu prednisona oral na dose de 60mg por 23 dias. O outro paciente recebeu metilprednisolona na dose de 125mg por 11 dias, seguida de prednisona oral na dose de 70mg, reduzida para 50mg por 21 dias, bem como imunoglobulina na dose de 60g e 80g em dias sucessivos, seguida de 4 dias de imunoglobulina na dose 40g diariamente por mais 5 dias. Três outros pacientes com menor grau de plaquetopenia (grau 1 ou 2) foram retirados do estudo por parte dos investigadores. Após os dois casos de trombocitopenia, um protocolo de monitoramento de plaquetas com dosagens a cada 2 semanas foi estabelecido, com um limiar de <100.000 plaquetas para redução da frequência das doses para cada 2 semanas, e um novo limiar passando de 75.000 para 50.000 plaquetas por microlitro para interrupção da medicação. Após essas medidas, nenhum paciente apresentou declínio de plaquetas <50.000 por microlitro, e não ocorreu descontinuação das doses relacionada à plaquetopenia. Ocorreu redução da frequência das doses de volanesorsena em 13 pacientes, sendo que, em 9 pacientes, estas foram relacionadas à plaquetopenia. Houve 14 pacientes randomizados para volanesorsena *versus* 2 no grupo placebo que não completaram as 52 semanas de tratamento. Nove descontinuaram por eventos adversos, sendo 5 por plaquetopenia e 4 por outros efeitos adversos relacionados à volanesorsena. Outros 4 retiraram voluntariamente o consentimento. Não houve morte durante o estudo.^23^

O estudo Re-FOCUS^196^ foi uma pesquisa retrospectiva global, aberta, realizada com pacientes com SQF que receberam volanesorsena por 3 meses ou mais na fase de extensão aberta do estudo. A pesquisa incluiu questões sobre experiências dos pacientes antes e após o tratamento com volanesorsena. Vinte e dois participantes tinham recebido volanesorsena por uma mediana de 222 dias. Volanesorsena reduziu significantemente o número de sintomas por paciente nos domínios físico, emocional e cognitivo. Houve redução significante nos episódios de esteatorreia, dor pancreática, e constante preocupação sobre um ataque de dor ou de pancreatite. Os participantes reportaram, ainda, que volanesorsena melhorou o manejo dos sintomas e reduziu a interferência da SQF nas responsabilidades do trabalho, ou na vida escolar. Houve reduções no impacto negativo da SQF na vida pessoal, profissional ou social. O tratamento com volanesorsena teve o potencial de reduzir a carga da doença em pacientes com SQF pela modulação nos múltiplos domínios de sintomas.

A volanesorsena foi aprovada pela Anvisa em 23 de agosto de 2021com base nos dados do estudo Approach e Compass, e tem indicação para pacientes adultos (acima de 18 anos) com confirmação genética de SQF e alto risco de pancreatite.^197^ O fármaco tem aprovação da agência europeia (European Medicines Agency) para uso em adultos com SQF desde 2014.

A volanesorsena não foi aprovada pela Food and Drug Administration (FDA), embora tenha sido avaliada no estudo Approach em pacientes com SQF. A doença é considerada ultrarrara e debilitante. A SQF causa pancreatites de ocorrência imprevisível e potencialmente fatais, complicações crônicas decorrentes de dano permanente ao órgão e impacto severo na vida diária dos pacientes. A característica típica da SQF consiste nos níveis muito elevados de triglicérides. Os resultados do estudo de fase 3 Approach – o maior estudo conduzido com pacientes portadores de SQF – mostraram que, em comparação ao placebo, o tratamento com a volanesorsena reduziu os triglicérides em 77% (-94% quando comparado ao placebo). As recomendações das sociedades médicas são para a redução dos triglicérides como alvo do tratamento dos pacientes com SQF. Os eventos adversos mais comuns foram as reações no local de injeção e redução na contagem de plaquetas.

A alegação do FDA foram questões de segurança, especialmente risco de sangramento devido à plaquetopenia, apesar das recomendações para mitigar efeitos adversos. Durante o estudo, quando foi detectada a possibilidade de ocorrência de plaquetopenia, o manejo desse evento adverso foi feito com monitoramento de plaquetas a cada 15 dias, podendo ser mais frequente conforme os exames subsequentes. Da mesma maneira, foi recomendado o espaçamento de doses de acordo com a contagem de plaquetas.

## 13. Aspectos Sociais, Psicológicos e Impacto Econômico da Doença

A variabilidade na precocidade, as diferenças na severidade dos sintomas e as variações no grau de limitações funcionais decorrentes do estado físico dos pacientes são características da expressão da SQF que interagem com outros aspectos, tais como perfil sociodemográfico e econômico; características de personalidade; fatores psicossociais e sociocognitivos; habilidades pessoais de enfrentamento de situações adversas em saúde; capacidade de exercer a autorregulação e manter o sentido de eficácia no contexto do adoecimento.^198^ A articulação de todos esses aspectos citados e de outros aspectos contextuais agrega complexidade ao manejo da SQF, podendo, além de interferir na habilidade adaptativa dos pacientes e cuidadores, demandar diferentes desenhos de intervenção médica centrados na singularidade das pessoas afetadas.

A falta de reverberação da fala dos pacientes na comunicação social, causada pela ausência do tema no discurso comum e sua restrição no discurso científico,^199^ estabelece um padrão de silêncio acerca da SQF. A falta de familiaridade para com essa condição, na sociedade médica, é agravante do estresse biopsicossocial experimentado pelas pessoas afetadas, o que leva a empreender uma peregrinação entre especialidades em busca da realização do diagnóstico, que, via de regra, acontece tardiamente e não conduz a resposta terapêutica medicamentosa eficaz. As lacunas na comunicação e no conhecimento impõem aos pacientes e cuidadores o desafio de conviver com uma doença que provoca limitadas reações empáticas, uma vez que não apresenta sentido e significado socialmente construído e tampouco identidade clinicamente reconhecida.^199^ Nesse panorama, para além do grave efeito deletério da doença no estado de saúde e na capacidade funcional, é relevante o desdobramento do estado da arte na vida real.

Estudo^200^ sobre a qualidade de vida dos pacientes com SQF, ao demonstrar a validade dos instrumentos de autorrelato, no contexto da doença rara, coloca em evidência o forte impacto negativo do tratamento, realizado fundamentalmente por meio de restritivo controle alimentar. Ao dar a conhecer qual a experiência da doença no cotidiano,^201^ o quanto adoecer prejudica a percepção pessoal de satisfação com a qualidade de vida e o estado de saúde,^202^ e deixar entrever o impacto do tratamento na capacidade adaptativa das pessoas afetadas,^203^ amplia a conscientização acerca dos desdobramentos psicossociais da SQF. Na Quadro 2 , estão listados os aspectos abordados na autoavaliação da qualidade de vida por meio de instrumentos de autorrelato.

**Quadro 2:** – Aspectos elencados na avaliação da qualidade de vida relacionada à saúde (QVRS)

Avaliação pessoal do:
Grau das limitações no desempenho de atividades do dia a dia por interferência do estado clínico
Grau das limitações na capacidade funcional para realizar atividades cotidianas de esforço leve, moderado e intenso
Estado geral de saúde
Grau de interferência da dor no cotidiano
Grau de interferência da saúde geral (física e mental) nas relações interpessoais
Grau de vitalidade no cotidiano (grau de envolvimento e motivação com a vida)
Grau das limitações no desempenho de atividades do dia a dia por interferência do estado emocional ou cognitivo
Estado de humor predominante no tempo definido

Ao possibilitar a escuta das pessoas afetadas pela doença, de modo estruturado e padronizado, o emprego do instrumento psicométrico de autorrelato permite romper com o padrão de silenciamento, característico das doenças raras ou pouco conhecidas.^199^ Embora os estudos de doenças de baixa prevalência contemplem número reduzido de sujeitos, em comparação aos realizados no enquadre das doenças crônicas e frequentes, são capazes de retratar a realidade dos pacientes e cuidadores e apontar tendências, e podem colaborar na orientação de estratégias comportamentais de enfrentamento.

### 13.1. Aspecto Social na Síndrome da Quilomicronemia Familiar

O paciente diagnosticado com doença rara, também nomeada doença órfã, é uma pessoa que, em alguma medida, perde suas referências sociais e se afasta do modo de cuidado à saúde usual, de senso comum, passando a depender dos norteamentos técnico-científicos na condução da doença. A carência de informações sobre a história da doença na vida real e a falta de diretrizes, ou posicionamentos norteadores de condutas e orientações médicas seguras e eficazes, impactam a habilidade pessoal na construção das rotinas, de projetos e manutenção das relações interpessoais como idealizadas pelos pacientes. O desconhecimento sobre a doença interfere no sentido de pertencimento, sustenta sentimentos de desamparo e isolamento. A invisibilidade da doença no cotidiano reduz a chance de os pacientes e cuidadores contarem com suporte social.^198^ Estudo^204^ evidencia que o forte sentimento de incompreensão pode impulsionar os pacientes e cuidadores a criarem respostas adaptativas restauradoras de familiaridade e pertencimento em ambientes religiosos.

**Figura 1:**
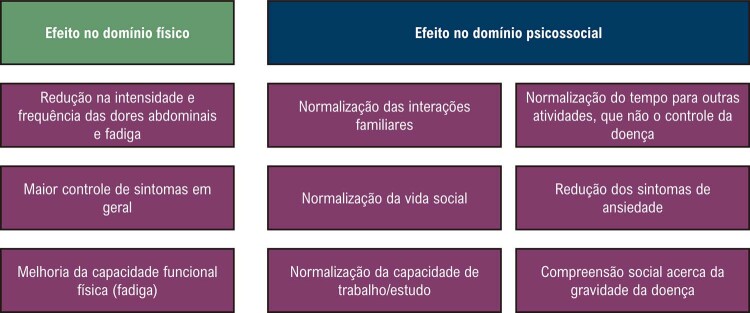
– Expectativas do paciente com SQF diante da aderência ao tratamento

Tem se evidenciado^204^ que as lacunas no conhecimento médico sobre a SQF dificultam a comunicação na práxis clínica. A falta de entendimento acerca dos objetivos da proposta terapêutica pode conduzir os pacientes a alimentarem expectativas pouco realistas quanto ao alcance do tratamento. A Figura 1 mostra quais as expectativas mais frequentes dos pacientes relacionadas à aderência ao tratamento na SQF.^202^

Evidenciados os aspectos físicos e psicossociais mais gravemente afetados pela SQF, na perspectiva do paciente, vale sublinhar que a crença de recuperação da condição de normalidade experimentada antes da experiência de adoecimento, a esperança depositada no tratamento, pode ser melhor manejada conforme os profissionais se encontrem mais atualizados e hábeis a realizar e comunicar o diagnóstico e as evidências que sustentam orientações terapêuticas, e conversar sobre os resultados esperados.^204^

### 13.2. Aspectos Psicológicos na Síndrome da Quilomicronemia Familiar

Sentimento de impotência diante da doença, sintomas de fadiga e confusão mental são aspectos de campo interdisciplinar que podem persistir durante toda a vida dos pacientes com SQF. A preocupação quanto ao efeito da doença na saúde e na vida, ao longo do tempo, o desejo de ser capaz de viver uma vida normal e a preocupação com o impacto financeiro da doença afetam sobremaneira a estabilidade emocional dos pacientes e cuidadores, podendo produzir sentimentos de baixa autoestima e ansiedade, interferir na habilidade de raciocínio e de elaboração de soluções, e reduzir a qualidade do sono.^206^ Depressão, sentimentos de constrangimento, vergonha e inadequação social, percepção de mudanças no funcionamento cognitivo, por influência de dificuldades de concentração e memória, são aspectos da doença que corroboram para o declínio na qualidade de vida pessoal e profissional dos afetados.^207^ Segundo os pacientes conviver com a SQF consome o tempo e exaure a energia física e mental, tornando-os incapazes de se projetarem na vida.^208^ Revisão sistemática^199^ indica que os adultos com diagnóstico de SQF podem expressar significativos prejuízos psicológicos relacionados à falta de autonomia e de liberdade na condução da vida, para além da doença. Tal aspecto precisa ser mais bem conhecido na SQF.

#### 13.2.1. Os Pais das Crianças com Diagnóstico de Síndrome da Quilomicronemia Familiar

A SQF é uma doença que se apresenta no final da infância e adolescência, embora alguns casos sejam encontrados nos primeiros anos de vida e entre neonatos.^209^ Doenças raras são desafiadoras não somente para os pacientes, mas também para aqueles familiares que lhes oferecem cuidado. Estudo^210^ identifica a elevada frequência de relatos parentais acerca da falta de suporte social e ausência de empatia por parte dos profissionais de saúde, o que se expressa em forma de queixas recorrentes de falta de informação e orientação, de modo geral, e de falta de aconselhamento quanto ao modo adequado de interagir/agir com a criança doente. Tal estudo mostra que os pais tendem a expressar mais preocupações com o futuro, e as mães, com o tempo presente. Além disso, evidencia que o relato sobre a avaliação pessoal de prejuízo na qualidade das relações sociais, familiares e profissionais é mais comum entre as mães, pois tende a ocupar mais de seu tempo com os cuidados básicos e a rotina diária. Tais diferenças, interpretadas com relação às questões de gênero, precisam ser melhor conhecidas.

## 13.3. Para Reduzir os Impactos da Doença: Modos de Enfrentamento

A aderência às recomendações gerais, usualmente dispostas como consensos médicos, é essencial para a promoção e a prevenção da saúde em âmbito primário, secundário e nos processos de reabilitação. Entre os comportamentos em saúde, a aderência terapêutica é um dos comportamentos de autorregulação mais estudados, e refere-se à participação ativa do paciente no manejo da doença para fim de preservação da saúde e qualidade de vida no contexto do adoecimento.^211^ Vale lembrar que a proposta de aderência terapêutica total e permanente pode gerar conflitos pessoais e sociais, e encontrar resistência por parte dos pacientes ou falta de colaboração social, na medida em que pode impactar projetos de vida pactuados/interpessoais ao interferir na decisão de ter filhos, na capacidade de trabalhar, no tempo livre para aproveitar momentos de lazer etc.^207^

### 13.3.1. Modelos Ativos e Passivos de Enfrentamento: Foco no Paciente

Visando a maior sucesso na aderência terapêutica na SQF, é indispensável o envolvimento do paciente nas decisões. Para tanto, preconiza-se lançar mão de estratégias passivas e ativas de enfrentamento. Dados da revisão sistemática^199^ mostram que são exemplos de abordagens passivas de enfrentamento: obtenção/busca por informação; aconselhamento clínico; aconselhamento genético; educação em saúde. São exemplares de abordagens ativas de enfrentamento na atuação/ação comportamental, no caso da SQF: autocontrole na restrição no consumo de gorduras, álcool e carboidratos; autorregulação na ingestão de remédios sem indicação médica, evitando a interação medicamentosa prejudicial; autoadministração dos fármacos indicados para redução das concentrações plasmáticas de triglicérides; realização de exames de acompanhamento na frequência indicada pelo médico de referência.

Estudo^211^ que investigou o efeito do uso da internet na capacidade adaptativa de pais de crianças com doenças raras mostrou que o ganho de conhecimento é essencial para a adaptação gradual ao contexto de saúde. O referido estudo ressalta que a livre busca por *expertise* pelo paciente tanto pode elevar seu sentido de eficácia como aumentar a expressão de sintomas de ansiedade. Enquanto realidade contemporânea, o impacto da busca por formação/informação *online* pelos pacientes e cuidadores, no processo adaptativo à SQF, precisa ser mais bem conhecido.

### 13.3.2. Modelo Social de Enfrentamento: Foco nos Pares

Sabe-se que o suporte social obtido em grupos de pares pode contribuir melhorando a percepção de bem-estar geral e gerar motivação para o exercício da autorregulação.^212^ O estudo CONNEC^213^ mostra que as pessoas afetadas pela SQF podem se beneficiar ao estabelecer contato com outros portadores da doença. Esse estudo sugere que a participação em grupos afins, seja por meio de leitura de textos, participação em *sites* e rodas de conversa presencial ou *online* , interagindo ou apenas observando as narrativas de outros pacientes, pode influenciar positivamente a autopercepção de qualidade de vida, a reavaliação da severidade dos sintomas físicos e a redução da sintomatologia psiquiátrica, além de mitigar o estresse psicossocial. Como parte da implantação de medidas amplas de enfrentamento e manejo da doença, preconiza-se o preenchimento das lacunas técnico-científicas, o incentivo dos pacientes à socialização terapêutica e a difusão do conhecimento sobre o efeito psicossocial adverso da SQF, o que pode colaborar acelerando processos de construção da identidade social da doença e estabelecimento de *expertise* na atenção à saúde.^93 , 94^

## 13.4. Custo-efetividade do Manejo de Riscos Psicossociais

Sabendo-se que a avaliação custo-eficácia das intervenções em saúde busca apontar soluções de menor gasto não relacionado à doença, por meio das quais, a alocação do investimento para que seja contabilizado o melhor resultado,^214^ é de se admitir que a aplicação em recursos terapêuticos colaborativos, que contemplem intervenções atentas aos aspectos clínicos e sintomas psicoemocionais,^215^ traga retorno custo-eficaz, visto que manifestações psiquiátricas, embora não específicas da SQF, prejudicam a aderência terapêutica e precipitam recorrentes urgências e internações hospitalares. ^219^ Nesse sentido, corrobora a revisão de literatura^216^ que mostra haver robusta evidência de que o investimento em ações combinadas, de intervenção nas doenças cardiovasculares junto à intervenções nos quadros de ansiedade e depressão, acarreta resultado custo-eficaz positivo. Finalmente, o estudo ReFOCUS^196^ mostra que o tratamento farmacológico adequado pode produzir o controle da evolução da doença, reduzir o estresse gerado pelo controle alimentar restritivo severo e, inclusive, modificar expectativas em relação ao futuro. Nessa perspectiva, não cabe dúvida acerca da estreita articulação da SQF com aspectos psicossociais, e do potencial custo-eficaz projetado nos estudos e intervenções que buscam desenvolver tratamento medicamentoso efetivo para pacientes diagnosticados para SQF.^217^

## 14. Resumo das Recomendações

**Table:** 

	Grau de recomendação	Nível de evidência
A coleta de sangue para as dosagens de triglicérides no adulto deve ser feita em jejum de 12 horas, com alimentação habitual, sem álcool (72 horas) e sem exercícios físicos (24 horas). Para crianças, o tempo varia de acordo com a faixa etária. Para lactentes, até 1 ano, o jejum é de 3 horas ou imediatamente antes da próxima mamada. Em não lactentes, de 2 a 5 anos, o jejum é de 6 horas; e, em crianças acima de 5 anos e adolescentes, o jejum é de 12 horas (Grau de Recomendação: I, Nível de Evidência: C).	I	C
Para a suspeita diagnóstica de SQF, após afastadas as causas secundárias de HTG, recomenda-se que os valores de triglicérides sejam: 1) adultos em jejum de 12 horas com triglicérides >1.000mg/dL, em três coletas diferentes; 2) crianças e adolescentes, com valores de triglicérides >880mg/dL, independentemente do tempo de jejum, em três coletas diferentes; 3) em crianças e adultos, a presença de uma dosagem de triglicérides <170mg/dL **exclui** a investigação de hiperquilomicronemia (Grau de Recomendação: I, Nível de Evidência: C).	I	C
Valores de triglicérides >1.000mg/dL aumentam o risco de pancreatite nos pacientes com SQF (Grau de Recomendação: IIa, Nível de Evidência: C).	IIa	C
Os valores de LDL-c na SQF são inacurados por qualquer método de dosagem. Se calculados, devem utilizar a fórmula de Martin ou, preferencialmente, ser dosados pelo método direto (Grau de Recomendação: I, Nível de Evidência: C).	I	C
O escore diagnóstico de SQF é ferramenta útil na suspeita diagnóstica de SQF, sendo recomendado como triagem para o teste genético (Grau de Recomendação: I, Nível de Evidência: C).	I	C
Este documento não recomenda a dosagem da atividade da lipoproteína lipase com heparina, pois esse teste pode ter capacidade discriminativa limitada em portadores de variantes comuns (Grau de Recomendação: III, Nível de Evidência: C).	III	C
O sequenciamento genético dos genes *LPL* , *APOC2* , *APOA5* , *GPIHBP1* e *LMF1* confere diagnóstico de certeza para SQF se houver homozigose, heterozigotos duplos ou compostos para variantes patogênicas ou provavelmente patogênicas (Grau de Recomendação: I, Nível de Evidência: C).	I	C
Diante de um caso confirmado de SQF, o aconselhamento genético deve ser realizado visando calcular o risco de ocorrência ou de recorrência da condição, tanto para tomada de decisões quanto para escolha do método contraceptivo, especialmente em uniões consanguíneas (Grau de Recomendação: I, Nível de Evidência: C).	I	C
A terapia nutricional deve ter como recomendações gerais: Restrição do consumo de gorduras (10 a 15% do VCT) Exclusão de açúcares de adição (sacarose e xarope de milho) Exclusão de sucos de frutas concentrados Exclusão de bebidas alcoólicas Consumo de carboidratos complexos em quantidades adequadas Garantia da adequação de ácidos graxos essenciais Monitoramento do consumo de vitaminas lipossolúveis, com suplementação quando necessário Inclusão de TCM com a finalidade de aporte calórico, de acordo com tolerância. (Grau de Recomendação: I, Nível de Evidência: C).	I	C
A infusão de heparina endovenosa na pancreatite hipertrigliceridêmica (PH) nos pacientes com SQF não é recomendada. (Grau de Recomendação: III, Nível de Evidência: C).	III	C
O uso de heparina de baixo peso molecular está indicado como profilaxia para trombose venosa profunda na PH nos pacientes com SQF. (Grau de Recomendação: IIa, Nível de Evidência: C).	IIa	C
A insulina endovenosa deve ser utilizada apenas em pacientes com diabetes tipo 1 e 2 descompensado, para controle glicêmico, na PH nos pacientes com SQF. (Grau de Recomendação: IIa, Nível de Evidência: C)	IIa	C
A plasmaférese deve ser indicada para pacientes com PH nos portadores de SQF de forma individualizada. Os candidatos potenciais seriam os pacientes que apresentam PH grave, ou que persistem com valores de triglicérides >1.000mg/dL, após as primeiras 24 a 48 horas. (Grau de Recomendação: IIb, Nível de Evidência: C).	IIb	C
A indicação de plasmaférese na gestação, apesar de segura e eficaz, deve ser individualizada pela escassez de evidências até o momento. (Grau de Recomendação: IIb, Nível de Evidência: C).	IIb	C
O uso de antissentido anti-APOC3 está indicado para adultos acima de 18 anos com diagnóstico genético confirmado de SQF que não sejam responsivos ao tratamento usual e sob alto risco de pancreatite (Grau de Recomendação: I, Nível de Evidência: B).	I	C
O monitoramento de plaquetas durante o tratamento com o antissentido anti-APOC3 deve ser feito inicialmente a cada 2 semanas, e ajustado conforme a contagem de plaquetas (Grau de Recomendação: I, Nível de Evidência: B).	I	B
O tratamento com o antissentido anti-APOC3 deve ter suas aplicações espaçadas caso as plaquetas estejam <100.000/uL, e o fármaco deve ser interrompido se a contagem de plaquetas for <75.000/uL (Grau de Recomendação: I, Nível de Evidência: B).	I	B
