# Supplementary material for: Posicionamento Brasileiro sobre Síndrome da Quilomicronemia Familiar – 2023
Source: Arq Bras Cardiol. 2023 Mar 23;120(4):e20230203. [Article in Portuguese] doi: 10.36660/abc.20230203 (PMC10348387; doi:10.36660/abc.20230203)
Supplement: Translation

# Translation: Brazilian Position Statement for Familial Chylomicronemia Syndrome – 2023

**Authors:** Maria Cristina de Oliveira Izar, Raul Dias dos Santos, Marcelo Heitor Vieira Assad, Antonio Carlos Palandri Chagas, Alceu de Oliveira Toledo, Ana Cláudia Cavalcante Nogueira, Ana Cristina Carneiro Fernandes Souto, Ana Maria Lottenberg, Ana Paula Marte Chacra, Carlos Eduardo dos Santos Ferreira, Charles Marques Lourenço, Cynthia Melissa Valerio, Dennys Esper Cintra, Francisco Antonio Helfenstein Fonseca, Gustavo Aguiar Campana, Henrique Tria Bianco, Josivan Gomes de Lima, Maria Helane Costa Gurgel Castelo, Marileia Scartezini, Miguel Antonio Moretti, Natasha Slhessarenko Fraife Barreto, Rayana Elias Maia, Renan Magalhães Montenegro, Renato Jorge Alves, Roberta Marcondes Machado Figueiredo, Rodrigo Ambrosio Fock, Tânia Leme da Rocha Martinez, Viviane Zorzanelli Rocha Giraldez

**Affiliations:** 1 Brazil Universidade Federal de São Paulo (UNIFESP), São Paulo, SP – Brazil; 2 Brazil Universidade de São Paulo (USP), São Paulo, SP – Brazil; 3 Brazil Instituto Nacional de cardiologia (INC), Rio de Janeiro, RJ – Brazil; 4 Brazil Faculdade de Medicina do ABC, São Caetano do Sul, SP – Brazil; 5 Brazil Universidade Estadual de Ponta Grossa (UEPG), Ponta Grossa, PR – Brazil; 6 Brazil Hospital de Base do Distrito Federal, Brasília, DF – Brazil; 7 Brazil Instituto do Coração (Incor) do Hospital das Clínicas da Faculdade de Medicina da Universidade de São Paulo (HCFMUSP), São Paulo, SP – Brazil; 8 Brazil Laboratório de Lípides (LIM 10) do Hospital das Clínicas da Faculdade de Medicina da Universidade de São Paulo (HCFMUSP), São Paulo, SP – Brazil; 9 Brazil Hospital Israelita Albert Einstein (HIAE), São Paulo, SP – Brazil; 10 Brazil Faculdade de Medicina de São José do Rio Preto (FAMERP), São José do Rio Preto, SP – Brazil; 11 Brazil Instituto Estadual de Diabetes e Endocrinologia Luiz Capriglione (IEDE-RJ), Rio de Janeiro, RJ – Brazil; 12 Brazil Universidade Estadual de Campinas (UNICAMP), Campinas, SP – Brazil; 13 Brazil Alliar Medicina Diagnóstica, São Paulo, SP – Brazil; 14 Brazil Hospital Universitário Onofre Lopes da Universidade Federal do Rio Grande do Norte (UFRN), Natal, RN – Brazil; 15 Brazil Diagnósticos da América S.A. (DASA), Fortaleza, CE – Brazil; 16 Brazil Universidade Federal do Paraná (UFPR), Curitiba, PR – Brazil; 17 Brazil Universidade Federal de Mato Grosso (UFMT), Cuiabá, MT – Brazil; 18 Brazil Universidade Federal da Paraíba (UFPB), João Pessoa, PB – Brazil; 19 Brazil Complexo Hospitalar da Universidade Federal do Ceará (UFCE), Empresa Brasileira de Serviços Hospitalares (EBSERH), Fortaleza, CE – Brazil; 20 Brazil Hospital Santa Casa de Misericórdia de São Paulo, São Paulo, SP – Brazil; 21 Brazil Faculdade Israelita de Ciências da Saúde Albert Einstein (FICSAE), São Paulo, SP – Brazil; 22 Brazil Hospital Beneficência Portuguesa de São Paulo, São Paulo, SP – Brazil

**Table:** 

**Brazilian Position Statement for Familial Chylomicronemia Syndrome – 2023**	
**The report below lists declarations of interest as reported to the SBC by the experts during the period of the development of these statement, 2022.**	
**Expert**	**Type of relationship with industry**
Alceu de Oliveira Toledo Júnior	Nothing to be declared
Ana Cláudia Cavalcante Nogueira	Financial declaration A - Economically relevant payments of any kind made to (i) you, (ii) your spouse/partner or any other person living with you, (iii) any legal person in which any of these is either a direct or indirect controlling owner, business partner, shareholder or participant; any payments received for lectures, lessons, training instruction, compensation, fees paid for participation in advisory boards, investigative boards or other committees, etc. From the brazilian or international pharmaceutical, orthosis, prosthesis, equipment and implants industry: - Servier: Vastarel/Angina. Other relationships Funding of continuing medical education activities, including travel, accommodation and registration in conferences and courses, from the brazilian or international pharmaceutical, orthosis, prosthesis, equipment and implants industry: - PTC: Waylivra/FCS. Any economically relevant equity interest in companies in the healthcare or education industry or in any companies competing with or supplying to SBC: - Teaching and clinical research area.
Ana Cristina Carneiro Fernandes Souto	Nothing to be declared
Ana Maria Pitta Lottenberg	Financial declaration A - Economically relevant payments of any kind made to (i) you, (ii) your spouse/partner or any other person living with you, (iii) any legal person in which any of these is either a direct or indirect controlling owner, business partner, shareholder or participant; any payments received for lectures, lessons, training instruction, compensation, fees paid for participation in advisory boards, investigative boards or other committees, etc. From the brazilian or international pharmaceutical, orthosis, prosthesis, equipment and implants industry: - PTC Therapeutics: Financial support for lectures.
Ana Paula Marte Chacra	Nothing to be declared
Antonio Carlos Palandri Chagas	Financial declaration A - Economically relevant payments of any kind made to (i) you, (ii) your spouse/partner or any other person living with you, (iii) any legal person in which any of these is either a direct or indirect controlling owner, business partner, shareholder or participant; any payments received for lectures, lessons, training instruction, compensation, fees paid for participation in advisory boards, investigative boards or other committees, etc. From the brazilian or international pharmaceutical, orthosis, prosthesis, equipment and implants industry: - Novo Nordisk: Semaglutida. Other relationships Funding of continuing medical education activities, including travel, accommodation and registration in conferences and courses, from the brazilian or international pharmaceutical, orthosis, prosthesis, equipment and implants industry: - Novo Nordisk: Semaglutida. Any other interest (financial or other) that should be declared considering the position taken in SBC that has not been expressly listed above: - Member of the Vita Nova Institute Scientific Committee.
Carlos Eduardo dos Santos Ferreira	Nothing to be declared
Charles Marques Lourenço	Other relationships Funding of continuing medical education activities, including travel, accommodation and registration in conferences and courses, from the brazilian or international pharmaceutical, orthosis, prosthesis, equipment and implants industry: - Sanofi: Biomarin/Achondroplasia; PTC: NiemannPick B/AADC deficiency.
Cynthia Melissa Valerio	Financial declaration A - Economically relevant payments of any kind made to (i) you, (ii) your spouse/partner or any other person living with you, (iii) any legal person in which any of these is either a direct or indirect controlling owner, business partner, shareholder or participant; any payments received for lectures, lessons, training instruction, compensation, fees paid for participation in advisory boards, investigative boards or other committees, etc. From the brazilian or international pharmaceutical, orthosis, prosthesis, equipment and implants industry: - Novo Nordisk: Saxenda, Rybelsus, Ozempic; PTC: Waylivra; Amryt: Myalept. B - Research funding under your direct/personal responsibility (directed to the department or institution) from the Brazilian or international pharmaceutical, orthosis, prosthesis, equipment and implants industry: - Amryt: Metreleptina.
Dennys Esper Cintra	Nothing to be declared
Francisco Antonio Helfenstein Fonseca	Nothing to be declared
Gustavo Aguiar Campana	Nothing to be declared
Henrique Tria Bianco	Nothing to be declared
Josivan Gomes de Lima	Financial declaration A - Economically relevant payments of any kind made to (i) you, (ii) your spouse/partner or any other person living with you, (iii) any legal person in which any of these is either a direct or indirect controlling owner, business partner, shareholder or participant; any payments received for lectures, lessons, training instruction, compensation, fees paid for participation in advisory boards, investigative boards or other committees, etc. From the brazilian or international pharmaceutical, orthosis, prosthesis, equipment and implants industry: - Novo Nordisk: Semaglutida. Other relationships Funding of continuing medical education activities, including travel, accommodation and registration in conferences and courses, from the brazilian or international pharmaceutical, orthosis, prosthesis, equipment and implants industry: - Novo Nordisk: Semaglutida.
Marcelo Heitor Vieira Assad	Financial declaration A - Economically relevant payments of any kind made to (i) you, (ii) your spouse/partner or any other person living with you, (iii) any legal person in which any of these is either a direct or indirect controlling owner, business partner, shareholder or participant; any payments received for lectures, lessons, training instruction, compensation, fees paid for participation in advisory boards, investigative boards or other committees, etc. From the brazilian or international pharmaceutical, orthosis, prosthesis, equipment and implants industry: - Novo Nordisk: Rybelsus. Other relationships Funding of continuing medical education activities, including travel, accommodation and registration in conferences and courses, from the brazilian or international pharmaceutical, orthosis, prosthesis, equipment and implants industry: - Novo Nordisk: Rybelsus.
Maria Cristina de Oliveira Izar	Financial declaration A - Economically relevant payments of any kind made to (i) you, (ii) your spouse/partner or any other person living with you, (iii) any legal person in which any of these is either a direct or indirect controlling owner, business partner, shareholder or participant; any payments received for lectures, lessons, training instruction, compensation, fees paid for participation in advisory boards, investigative boards or other committees, etc. From the brazilian or international pharmaceutical, orthosis, prosthesis, equipment and implants industry: - Amgen: Repatha; Amryt Pharma: Lojuxta; AstraZeneca: Dapagliflozina; Aché: Trezor, Trezete; Biolab: Livalo; Abbott: Lipidil; EMS: Rosuvastatina; Eurofarma: Rosuvastatina; Sanofi: Praluent, Zympass, Zympass Eze, Efluelda; Libbs: Plenance, Plenance Eze; Novo Nordisk: Ozempic, Victoza; Servier: Acertamlo, Alertalix; PTCBio: Waylivra. B - Research funding under your direct/personal responsibility (directed to the department or institution) from the Brazilian or international pharmaceutical, orthosis, prosthesis, equipment and implants industry: - PTCBio: Waylivra; Amgen: Repatha; Novartis: Inclisiran, Pelacarsen; NovoNordisk: Ziltivekimab. Other relationships Funding of continuing medical education activities, including travel, accommodation and registration in conferences and courses, from the brazilian or international pharmaceutical, orthosis, prosthesis, equipment and implants industry: - Novo Nordisk: Diabetes.
Maria Helane Costa Gurgel Castelo	Nothing to be declared
Marileia Scartezini	Nothing to be declared
Miguel Antonio Moretti	Nothing to be declared
Natasha Slhessarenko Fraife Barreto	Nothing to be declared
Raul Dias dos Santos Filho	Financial declaration A - Economically relevant payments of any kind made to (i) you, (ii) your spouse/partner or any other person living with you, (iii) any legal person in which any of these is either a direct or indirect controlling owner, business partner, shareholder or participant; any payments received for lectures, lessons, training instruction, compensation, fees paid for participation in advisory boards, investigative boards or other committees, etc. From the brazilian or international pharmaceutical, orthosis, prosthesis, equipment and implants industry: - Amgen: Evolocumabe; Astra Zenec:Dapagliflozina; Aché:/Rosuvastatina; PTC Therapeutics:Volanesorsen; Novartis: Inclisiran; Novo Nordisk: Semaglutide; Sanofi: Rosuvastatina; Libbs: Rosuvastatina. B - Research funding under your direct/personal responsibility (directed to the department or institution) from the Brazilian or international pharmaceutical, orthosis, prosthesis, equipment and implants industry: - Amgen: Evolocumabe; Sanofi: Alirocumabe; Novartis: Inclisiran; Esperion: Ácido Bempedóico; Novartis: Pelacarsen; Kowa: Pemafibrato. Other relationships Funding of continuing medical education activities, including travel, accommodation and registration in conferences and courses, from the brazilian or international pharmaceutical, orthosis, prosthesis, equipment and implants industry: - Amgen: Cholesterol; Ache: Cholesterol; AstraZeneca: Diabete; Libbs: Cholesterol; Novartis: Cholesterol; Novo Nordisk: Diabetes; Eli-Lilly: Diabetes; Merck: Diabetes; Biolab: Cholesterol.
Rayana Elias Maia	Financial declaration A - Economically relevant payments of any kind made to (i) you, (ii) your spouse/partner or any other person living with you, (iii) any legal person in which any of these is either a direct or indirect controlling owner, business partner, shareholder or participant; any payments received for lectures, lessons, training instruction, compensation, fees paid for participation in advisory boards, investigative boards or other committees, etc. From the brazilian or international pharmaceutical, orthosis, prosthesis, equipment and implants industry: - Amgen: PTC: Volanersorsen. Other relationships Funding of continuing medical education activities, including travel, accommodation and registration in conferences and courses, from the brazilian or international pharmaceutical, orthosis, prosthesis, equipment and implants industry: - Amgen: PTC: Volanersorsen.
Renan Magalhães Montenegro Junior	Financial declaration A - Economically relevant payments of any kind made to (i) you, (ii) your spouse/partner or any other person living with you, (iii) any legal person in which any of these is either a direct or indirect controlling owner, business partner, shareholder or participant; any payments received for lectures, lessons, training instruction, compensation, fees paid for participation in advisory boards, investigative boards or other committees, etc. From the brazilian or international pharmaceutical, orthosis, prosthesis, equipment and implants industry: - Boheringer, AstraZeneca, Novo Nordisk, PTC, Nestlé, Amryt, Jansen. Outros relacionamentos Financiamento de atividades de educação médica continuada, incluindo viagens, hospedagens e inscrições para congressos e cursos, provenientes da indústria farmacêutica, de órteses, próteses, equipamentos e implantes, brasileiras ou estrangeiras: - Novo Nordisk, Amryt, PTC, Boheringer, Abbott.
Renato Jorge Alves	Financial declaration A - Economically relevant payments of any kind made to (i) you, (ii) your spouse/partner or any other person living with you, (iii) any legal person in which any of these is either a direct or indirect controlling owner, business partner, shareholder or participant; any payments received for lectures, lessons, training instruction, compensation, fees paid for participation in advisory boards, investigative boards or other committees, etc. From the brazilian or international pharmaceutical, orthosis, prosthesis, equipment and implants industry: - Boheringer, AstraZeneca, Novo Nordisk, PTC, Nestlé, Amryt, Jansen. Other relationships Funding of continuing medical education activities, including travel, accommodation and registration in conferences and courses, from the brazilian or international pharmaceutical, orthosis, prosthesis, equipment and implants industry: - Novo Nordisk, Amryt, PTC, Boheringer, Abbott.
Roberta Marcondes Machado Figueiredo	Financial declaration A - Economically relevant payments of any kind made to (i) you, (ii) your spouse/partner or any other person living with you, (iii) any legal person in which any of these is either a direct or indirect controlling owner, business partner, shareholder or participant; any payments received for lectures, lessons, training instruction, compensation, fees paid for participation in advisory boards, investigative boards or other committees, etc. From the brazilian or international pharmaceutical, orthosis, prosthesis, equipment and implants industry: - PTC Therapeutics. Other relationships Funding of continuing medical education activities, including travel, accommodation and registration in conferences and courses, from the brazilian or international pharmaceutical, orthosis, prosthesis, equipment and implants industry: - PTC Therapeutics.
Rodrigo Ambrosio Fock	Other relationships Funding of continuing medical education activities, including travel, accommodation and registration in conferences and courses, from the brazilian or international pharmaceutical, orthosis, prosthesis, equipment and implants industry: - Biomarin.
Tânia Leme da Rocha Martinez	Nothing to be declared

Contents

1. Cover Letter 5

2. Document Objectives 6

3. Definition of Grades of Recommendation and Levels of Evidence 6

4. Definition of Hypertriglyceridemia (> 150 mg/dL), Severe Hypertriglyceridemia (> 500 mg/dL), and Chylomicronemia (> 1,000 mg/dL) 6

4.1. Introduction 6

4.2. Definition of Hypertriglyceridemia 7

5. Definition of Chylomicronemia – Familial Chylomicronemia Syndrome and Multifactorial Chylomicronemia Syndrome: Clinical and Laboratory Criteria and Patterns of Transmission 7

5.1. Introduction 7

5.2. Concepts 8

**5.2.1. Familial Chylomicronemia Syndrome** 8

**5.2.2. Multifactorial Chylomicronemia Syndrome** 8

6. Epidemiology of Familial Chylomicronemia Syndrome in the World and in Brazil 8

6.1. Definition of Familial Chylomicronemia Syndrome and Clinical Aspects 8

6.1.1. First Cases of Familial Chylomicronemia Syndrome 9

6.2. Epidemiology of Familial Chylomicronemia Syndrome in the World 9

6.3. Epidemiology of Familial Chylomicronemia Syndrome in Children 11

6.4. Epidemiology of Familial Chylomicronemia Syndrome in Brazil 11

7. Clinical Manifestations of Familial Chylomicronemia Syndrome, Differential Diagnosis, and Management of Complications 11

7.1. Clinical Manifestations in Familial Chylomicronemia Syndrome 11

**7.1.1. Hypertriglyceridemia** 12

**7.1.2. Abdominal Pain and Acute Pancreatitis** 12

**7.1.3. Neurological Manifestations** 12

**7.1.4. Hepatosplenomegaly** 12

**7.1.5. Eruptive Xanthomas** 12

**7.1.6. Lipemia Retinalis** 12

**7.1.7. Quality of Life** 12

**7.1.8. Diagnostic Score** 12

7.2. Differential Diagnosis 12

**7.2.1. Multifactorial Chylomicronemia Syndrome** 12

**7.2.2. Lipodystrophies** 13

7.3. Managing Complications of Familial Chylomicronemia Syndrome 13

**7.3.1. Acute Pancreatitis** 13

8. Laboratory Diagnosis of Familial Chylomicronemia Syndrome 14

8.1. Pre-analytical Phase (Patient Instructions) 14

**8.1.1. Collection Instructions** 14

**8.1.2. Pre-analytical Causes of Interference in Triglyceride Analyses** 14

**8.1.3. Pre-analytical Phase (Laboratory Instructions)** 14

8.2. Analytical Phase 15

**8.2.1. Methodologies Assessing Chylomicrons** 13

**8.2.1.1. Ultracentrifugation** 15

**8.2.1.2. Serum Appearance** 15

**8.2.1.3. Lipoprotein Electrophoresis** 15

**8.2.2. Methodologies for Assessing Triglycerides** 15

**8.2.3. Interferences to Triglyceride Results** 15

**8.2.4. Interferences of Triglycerides to Other Analytes** 15

**8.2.4.1. LDL-C** 15

**8.2.4.2. Platelets** 16

**8.2.4.3. Analytes with Colorimetric Analysis** 16

**8.2.4.4. Enzymes** 16

**8.2.4.5. Electrolytes** 16

**8.2.5. Laboratory Analyses for Differential Diagnosis** 16

**8.2.5.1. Post-heparin Lipoprotein Lipase Activity** 16

**8.2.5.2. Plasma ApoC3 Measurement** 16

8.3. Post-analytical Phase 16

**8.3.1. Recommendations for NOTES in Laboratory Reports** 16

9. Genetic Counseling and Stages of Diagnosis and Follow–up of Severe Hypertriglyceridemia 17

10. Nutritional Guidance for Chylomicronemia in Adults, Children, and Adolescents 18

10.1. Fatty Acid Classification and Absorption 18

10.2. Fat Absorption 19

10.3. Nutritional Treatment 19

**10.3.1. Fats** 19

**10.3.3. Carbohydrates** 20

**10.3.4. Alcohol** 20

**10.3.5. Infants and Early Childhood** 20

**10.3.6. Pregnant Women** 21

**10.3.7. General Recommendations** 21

10.4. Sample Menus 21

11. Apheresis 26

11.1. Diagnosis and Treatment 26

11.2. Nondrug Therapy 26

11.3. Pharmacological Treatment 26

11.4. Apheresis 26

12. New Therapies for the Treatment of Familial Chylomicronemia Syndrome 27

12.1. APOC3 27

**12.1.1 Antisense Inhibition of ApoC3** 27

13. Social and Psychological Aspects and Economic Impact of the Disease 27

13.1. Social Aspects in Familial Chylomicronemia Syndrome 29

13.2. Psychological Aspects in Familial Chylomicronemia Syndrome 30

**13.2.1. Parents of children diagnosed with Familial Chylomicronemia Syndrome** 30

13.3. Reducing the Impact of the Disease: Ways of Coping 30

**13.3.1. Active and Passive Models for Coping: Focus on the Patient** 31

**13.3.2. Social Model for Coping: Focus on Peers** 31

13.4. Cost-effectiveness in the Management of Psychosocial Risks 31

14. Summary of Recommendations 32

References 33

## 1. Cover Letter

Familial chylomicronemia syndrome (FCS) is a severe form of dyslipidemia characterized by multiple signs and symptoms associated with a deficiency in lipoprotein lipase or one of its cofactors, leading to compromised triglyceride metabolism. FCS has an autosomal recessive pattern of inheritance and affects approximately 1 to 2 people per million, but it may be more frequent in consanguineous relationships. Knowledge of this condition is still limited, often contributing to delayed diagnosis when complications have already set in. Patients with FCS may have recurrent abdominal pain, episodes of pancreatitis, eruptive xanthomas, *lipemia retinalis* , hepatosplenomegaly, and a milky appearance of serum.

In classic, severe forms, clinical symptoms are present at birth or even in childhood, but they may manifest at any age, especially in carriers of new mutations. Patients with FCS usually see several specialists before a diagnosis is made. The clinical presentation of FCS may also be indistinguishable from that of multifactorial chylomicronemia syndrome, which is more common and also has a genetic basis, although it is influenced by environmental and lifestyle factors. In addition, multifactorial chylomicronemia syndrome may result from conditions such as hypothyroidism, uncontrolled diabetes, kidney disease, alcohol abuse, and use of certain medications, which makes its diagnosis even more difficult.

A few centers in Brazil use a panel of causal genes to genetically confirm FCS. FCS diagnosis can be confirmed by the presence of a homozygous mutation in one of the causal genes or two different mutations in the same gene (compound heterozygote) or in different causal genes (double heterozygote), although in some cases, a causal mutation cannot be found. Validated algorithms may assist in the clinical suspicion of FCS and indicate which patients should undergo genetic testing.

FCS treatment requires a multidisciplinary approach, including a nutritionist and psychologist, among other health professionals, with the aim to maintain the individual’s well-being and nutritional status. Restriction of fats and simple carbohydrates and supplementation with fat-soluble vitamins and essential fatty acids should be recommended for life. Psychological support aims to help patients live with strict dietary restrictions.

Conventional pharmacological treatment is often less than 20% effective in reducing triglycerides, which is why patients’ hopes lie on the approval of new medications in Brazil that have proven beneficial in triglyceride reduction. Peculiar situations in the management of FCS are pregnancy and episodes of recurrent pancreatitis, for which mortality rates can be high and individualized treatment is required.

The purpose of this document is to make health professionals aware of the peculiar characteristics of FCS and to help them recognize early signs and symptoms and develop an adequate approach, mitigating patients’ suffering and the complications caused by a delayed diagnosis. Members of the Atherosclerosis Department of the Brazilian Society of Cardiology and renowned specialists from Brazil gathered together with the aim of describing in a clear and objective way the best scientific information available on FCS to improve clinical practice.

**Yours sincerely,**

Prof. Dra. Maria Cristina de Oliveira Izar

Prof. Dr. Raul Dias Santos

Prof. Dr. Antonio Carlos Palandri Chagas

Dr. Marcelo Heitor Vieira Assad

Coordinators

## 2. Document Objectives

This document aims to make health professionals, especially cardiologists, clinicians, and endocrinologists, aware of a very rare, underdiagnosed, and undertreated disease that causes intense suffering in those affected and which was not diagnosed until recently. Written by experts in the field, the Brazilian Position Statement for Familial Chylomicronemia Syndrome (FCS) fills a gap in the knowledge of epidemiological data in Brazil and the world about clinical manifestations, laboratory and genetic diagnoses, and differential diagnosis of other forms of severe hypertriglyceridemia (HTG). In addition, the peculiar nutritional management associated with the condition and the treatment of infants, children, and pregnant women and complications such as pancreatitis are highlighted in this document. Of note, a new antisense therapy against apolipoprotein C3 (ApoC3) has been recently approved in Brazil, with evidence of triglyceride reduction and prospects of preventing complications and improving the quality of life of patients.

## 3. Definition of Grades of Recommendation and Levels of Evidence

Classes (grades) of recommendation:

Class I – Conditions for which there is conclusive evidence or, if not, a consensus that the procedure is safe and useful/effective.

Class II – Conditions for which there is conflicting evidence and/or divergence of opinions on the safety and usefulness/efficacy of the procedure.

Class IIa – Evidence or opinion in favor of the procedure. The majority agrees.

Class IIb – Safety and usefulness/efficacy are less well established, and there is no predominance of opinions in favor of the procedure.

Class III – Conditions for which there is evidence and/or a consensus that the procedure is not useful/effective, and in some cases may be harmful.

Levels of evidence:

Level A – Data obtained from several large, randomized studies showing concurring results and/or a robust meta-analysis of randomized controlled trials.

Level B – Data obtained from a less robust meta-analysis, a single randomized study, or from nonrandomized (observational) studies.

Level C – Data obtained from consensual expert opinions.

## 4. Definition of Hypertriglyceridemia (> 150 mg/dL), Severe Hypertriglyceridemia (> 500 mg/dL), and Chylomicronemia (> 1,000 mg/dL)

### 4.1. Introduction

Some relevant factors should be considered before defining values and classifying HTG as mild, moderate, or severe. For lipid profile assessment, patients should maintain a stable metabolic state and their usual diet but should not consume alcohol 5 days before blood collection. Possible within-person biological variations and variations between laboratories should be considered when interpreting lipid measurements. Such variations can reach values of 10% for total cholesterol, high-density lipoprotein cholesterol (HDL-C), and low-density lipoprotein cholesterol (LDL-C) and up to 25% for triglycerides.^1^

The most recent Brazilian guidelines for dyslipidemia and diabetes state that fasting is not required for serum triglyceride assessment. However, if plasma triglyceride concentrations are > 400 mg/dL, plasma triglycerides should be measured again after a 12-hour fast due to the possibility of primary HTG, for which fasting is required.^2 , 3^ For HTG values > 400 mg/dL, the Friedewald formula, which is commonly used to calculate cholesterol fractions, is no longer used.^4^ Some publications suggest increased cardiovascular risk associated with postprandial HTG.^5 , 6^ In 2016, the European Atherosclerosis Society (EAS) and the European Federation of Clinical Chemistry and Laboratory Medicine guidelines stated that fasting was no longer required for lipid profile assessment.^7^

The Brazilian Guidelines for Dyslipidemia and Atherosclerosis classification of dyslipidemia is described in Table 1 .

**Table 1:** – Laboratory classification of dyslipidemia according to the Brazilian Guidelines for Dyslipidemia and Atherosclerosis ^2^

Isolated Hypercholesterolemia	Increased LDL-C (> 160 mg/dL)
Isolated Hypertriglyceridemia	Increased triglycerides (> 150 mg/dL with fasting or > 175 mg/dL without fasting)
Mixed hyperlipidemia	Increased LDL-C and triglycerides
Decreased HDL-C	HDL-C < 40 mg/dL in men or < 50 mg/dL in women, with or without increased LDL-C or triglycerides

*HDL-C: high-density lipoprotein cholesterol; LDL-C: low-density lipoprotein cholesterol. Class of recommendation: I; Level of evidence: C.*

Fredrickson’s classification of phenotypes ( Table 2 ) is based on lipoprotein fraction separation by electrophoresis and/or ultracentrifugation. Despite the known importance of this classification, it is currently only available in specialty centers and is no longer widely used in clinical practice. To demonstrate its relevance, we will describe patients with HTG with different phenotypic classifications according to primary lipoprotein abnormality: FCS (type I), familial combined hyperlipidemia (type IIb), dysbetalipoproteinemia (type III), simple primary HTG (type IV), and HTG with chylomicronemia (type V).^8 , 9^

**Table 2:** – Fredrickson’s classification of phenotypes ^8^

Classification	Increased lipoprotein
Type I	Chylomicrons
Type IIa	LDL
Type IIb	LDL and VLDL
Type III	IDL
Type IV	VLDL
Type V	Chylomicrons and VLDL

*IDL: intermediate-density lipoprotein; LDL: low-density lipoprotein; VLDL: very low-density lipoprotein.*

### 4.2. Definition of Hypertriglyceridemia

In laboratory tests, HTG is defined as plasma triglyceride concentrations > 150 mg/dL. However, if the lipid profile is measured without fasting, HTG is defined as triglycerides > 175 mg/dL.^1^

Thus, HTG may be classified into^10^ :

- Mild: plasma triglycerides > 150 mg/dL;
- Moderate: plasma triglycerides from 151 to 499 mg/dL;
- Severe: plasma triglycerides from 500 to 1,000 mg/dL;
- Very severe: plasma triglycerides > 1,000 mg/dL.

HTG results from the accumulation of lipoproteins rich in fatty acids and glycerol (such as very low-density lipoprotein [VLDL], intermediate-density lipoprotein, and remnants). Chylomicronemia is the main lipoprotein abnormality in severe and very severe forms, defined as the presence of circulating chylomicrons during fasting. The presence of chylomicrons can be detected in the blood if triglyceride concentrations are > 1,000 mg/dL; however, chylomicronemia is more likely to be detected when concentrations exceed 1,500 mg/dL. The severe and very severe forms of HTG are clinically relevant because of their association with a 2-fold increased risk of acute pancreatitis (AP), whose incidence increases by 3% for each 100 mg/dL > 1,000 mg/dL of triglyceridemia.^11^

## 5. Definition of Chylomicronemia – Familial Chylomicronemia Syndrome and Multifactorial Chylomicronemia Syndrome: Clinical and Laboratory Criteria and Patterns of Transmission

### 5.1. Introduction

Chylomicronemia is characterized by an accumulation of chylomicrons in the circulation and a significant increase in plasma triglyceride concentrations.

The higher the plasma triglyceride concentration, the greater the risk of pancreatitis. However, patients with concentrations > 1,000 mg/dL or very severe HTG are more likely to develop AP. To consider a diagnosis of FCS, laboratory abnormalities should be associated with the presence of clinical abnormalities since childhood or adolescence. Such abnormalities include *lipemia retinalis* , eruptive xanthomas, hepatosplenomegaly, and especially AP, which would help confirm the diagnosis of FCS.^12^

The severe and very severe forms of HTG are clinically relevant because of their association with a 2-fold increased risk of AP. AP is a potentially life-threatening condition that may also lead to a number of clinical complications, such as chronic pancreatitis, pancreatic insufficiency, and diabetes.^11 , 13^

Chylomicrons are formed by the incorporation of dietary lipids into Apos (A1, A2, A4, B48, C2, C3, and E) and then secreted into the mesenteric lymph.^14^ Lipoprotein lipase (LPL) is an enzyme located on the endothelial surface of adipose and muscle tissue capillaries; when activated, it initiates the process of hydrolysis of chylomicron triglycerides, generating chylomicron remnants. LPL activity is modulated by the action of apoC2 and apoA5, which act as cofactors in its activation, lipase maturation factor 1 (LMF1), which is necessary for the production of LPL in adipocytes and myocytes, and by the action of glycosylphosphatidylinositol anchored high-density lipoprotein binding protein 1 (GPIHBP1), which transports LPL from the interstitial space to the capillary lumen. Any alteration in the function and/or activation of LPL results in an increase in the half-life of chylomicrons in the bloodstream, consequently leading to chylomicronemia.^14^

There are two distinct forms of chylomicronemia: FCS and multifactorial chylomicronemia syndrome (MCS). These are, respectively, the prototypes of the monogenic and polygenic conditions underlying severe HTG of genetic origin. Chylomicronemia is estimated to affect 1:600 adults, but patients with FCS account for only 5%.^15^

The two forms of the disease can be differentiated by clinical and/or laboratory characteristics of patients. Patients with FCS usually present with pancreatitis, whereas those with MCS are more likely to have atherosclerotic cardiovascular disease. Early and accurate diagnosis of both conditions is essential for therapeutic success and mortality prevention.

The two forms are difficult to distinguish due to a considerable phenotypic overlap, and there are still many unanswered questions related to prevalence, clinical and genetic features, and clinical management.

### 5.2. Concepts

#### 5.2.1. Familial Chylomicronemia Syndrome

FCS is a serious and very rare metabolic disease characterized by chylomicronemia associated with recurrent episodes of abdominal pain and/or pancreatitis.

The worldwide estimate is that FCS affects 1 in every 500,000 to 1,000,000 people.^15 , 16^ The condition often manifests in childhood or adolescence and has been described in all ethnicities, with a higher prevalence in some geographic areas, such as Quebec, due to the founder effect.^17^

Also called Fredrickson type I hyperlipoproteinemia,^15^ FCS is a monogenic, autosomal recessive lipid disorder whose diagnosis is based on the detection of rare, biallelic mutations (homozygous or compound heterozygous) in LPL (> 80% of cases) or other genes that encode the proteins necessary for their activity (such as *APOC2* , *APOA5* , *GPIHBP1* , and *LMF1* ), leading to a dramatic reduction in chylomicron clearance.^15 , 18^ Typically, patients’ response to lipid-lowering drugs is limited, and thus treatment represents a clinical challenge. The cornerstone of FCS therapy consists of a dramatic reduction in fat intake (8% to 10% of total calories), which is difficult to maintain over time. Lifetime adherence to such an extremely restrictive treatment is difficult, negatively impacts quality of life, and does not completely eliminate the risk of pancreatitis in all patients. Recurrent AP occurs in 50% of patients with FCS; the overall associated mortality rate is 5% to 6% but increases to 30% in subgroups of patients who develop pancreatic necrosis or persistent multiple organ failure.^15 , 19^

#### 5.2.2. Multifactorial Chylomicronemia Syndrome

MCS, also called Fredrickson type V hyperlipoproteinemia, is an oligogenic or polygenic lipid disorder aggravated by the presence of comorbidities known to increase triglycerides (uncontrolled diabetes, hypothyroidism, pregnancy, obesity), environmental factors (alcohol abuse and diet rich in fats and simple sugars), and certain drugs, such as glucocorticoids, ethinylestradiol, and neuroleptics.^20^ MCS incidence tends to increase linearly with the increase in the prevalence of obesity, metabolic syndrome, and type 2 diabetes in the world population. In patients with this syndrome, chylomicronemia is intermittent and, in most cases, manifests later.^15^ It responds well to changes in lifestyle and treatment of secondary factors, with good response to triglyceride-lowering agents. MCS is characterized by an increased risk of AP, but the estimated odds ratio (OR) of 50 is clearly lower than the OR of 360 reported in patients with FCS.^16 , 21^

The two forms of chylomicronemia can be differentiated on the basis of lipoprotein electrophoresis or ultracentrifugation (presence of VLDL and chylomicrons in MCS; only chylomicrons in FCS). The current gold standard procedure for identifying patients with FCS is genetic testing or post-heparin LPL activity.^22^ Considering that the treatment for the two forms of the disease is very different, a correct diagnosis must be made. New therapies, such as apoC3 inhibitors, are under development to lower triglycerides in people with FCS.^23^

## 6. Epidemiology of Familial Chylomicronemia Syndrome in the World and in Brazil

### 6.1. Definition of Familial Chylomicronemia Syndrome and Clinical Aspects

FCS is a very rare inherited disease that affects approximately 1-2:1,000,000 people. It has an autosomal recessive mode of transmission and is characterized by very high concentrations of triglycerides (usually well above 1,000 mg/dL), turbid lipemic serum, with a milky aspect, *lipemia retinalis* , recurrent abdominal pain, eruptive xanthomas, episodes of recurrent pancreatitis, cognitive and neurological disorders, and impaired quality of life and sociability.^24^

However, the frequency of clinical manifestations in patients with FCS is variable. Eruptive xanthomas have been described in 17% to 23% of patients with FCS, *lipemia retinalis* in 4% to 36%, hepatosplenomegaly or splenomegaly alone in 12% to 25%, abdominal pain in 26% to 63%, pancreatitis in 60% to 88%, and multiple pancreatitis in 17% to 48%.^23 , 25 , 26^ Serum appearance is important to differentiate between the situations that increase free glycerol in the blood, leading to an overestimation of triglyceride levels, with no serum turbidity after 12 hours of refrigeration and excluding causes of hyperglycerolemia (recent physical activity, alcohol intake, acute liver disease, decompensated diabetes, parenteral nutrition, or intravenous medication containing glycerol).^27 , 28^

In FCS, severe HTG results from the inability to metabolize triglycerides and other fats. Fats are absorbed by the small intestine, where chylomicrons are formed. When LPL activity is normal, LPL participates in the hydrolysis of chylomicron triglycerides into free fatty acids via the LPL-dependent pathway.^20^ In FCS, chylomicrons, chylomicron remnants, and triglyceride-rich lipoproteins cannot be metabolized and accumulate in the plasma. Thus, the accumulation of triglycerides can impair pancreatic blood flow and activate inflammatory processes, resulting in AP.^19 , 29 - 30^

The role of LPL and its cofactors is crucial for understanding the metabolism of triglyceride-rich lipoproteins.^24^ LPL synthesis occurs intracellularly in adipocytes and smooth muscle cells. It is produced as a monomer, and adequate LPL dimerization is dependent on LMF-1. After this step, GPIHBP1, a glycoprotein involved in the transport of LPL to the capillary lumen, facilitates anchorage of LPL to the endothelial capillary, where it hydrolyzes the triglyceride content of chylomicrons and VLDL. ApoC2 and apoA5 participate as cofactors in LPL activation. Hydrolysis of triglycerides from these lipoproteins releases free fatty acids and monoglycerides, which are transported into myocytes or adipocytes, where they are used for energy production or lipid storage.^24^

Mutations in five different genes have been implicated in the development of FCS, all of which have an effect on LPL activity, which is responsible for removing triglycerides from chylomicrons and other triglyceride-rich lipoproteins in the circulation, breaking them down into free fatty acids. Patients with FCS have loss-of-function mutations in the *LPL* gene, leading to extremely high levels of chylomicrons in the circulation and, therefore, severe HTG. Other genes have also been described as cofactors in LPL activation, namely: *APOC2* , *APOA5* , *LMF1* , and *GPIHBP1* .^15^

#### 6.1.1. First Cases of Familial Chylomicronemia Syndrome

FCS was first described by Gaskins et al.^28^ in 1953, when they followed up three cases of familial idiopathic hyperlipoproteinemia in a family of eight people. The patients had a milky appearance of serum and markedly increased triglycerides. A low-fat diet followed by the administration of intravenous heparin greatly reduced triglycerides, suggesting that the defect was related to triglyceride removal from the circulation.^28^

This family was studied in 1960 and LPL, an enzyme anchored to the vascular endothelial surface and released from the wall by heparin, was suspected to be responsible for the lipid defect.^31^ When studying three brothers affected by the condition, the authors also suggested that another defect in addition to LPL could cause the so-called familial idiopathic hyperlipoproteinemia syndrome.

## 6.2. Epidemiology of Familial Chylomicronemia Syndrome in the World

Because FCS is a very rare disease, expert reports contribute greatly to prevalence estimates. Hegele et al.^32^ reported that, in a series of biological samples from 381 patients with triglycerides > 1,000 mg/dL, four patients (or 1%) had two large-effect loss-of-function mutations on both alleles of the *LPL* gene, which characterizes the classic autosomal recessive LPL deficiency. When considering patients with mutations in both alleles of the four so-called minor genes that modulate LPL activity – namely, *APOC2* , *APOA5* , *LMF1* , and *GPIHBP1* –, another four patients were identified, that is, another 1%.^33 , 34^

Patients with two mutations in the *LPL* gene or in its regulatory genes (compound heterozygotes) have two different loss-of-function mutations, and those with two heterozygous mutations in two different causal genes (double heterozygotes) added up to 1% more.^33 , 34^

Thus, it was estimated that approximately 3% of patients with severe HTG (triglycerides ≥ 1,000 mg/dL) in this sample had mutations in both alleles of genes that encode LPL or one of the proteins that modulate its activity. These patients may be homozygous, compound heterozygous, or double heterozygous. These conditions have been described among French Canadians from the province of Quebec, where the percentage of patients with two mutant alleles is higher due to a founder effect. Such prevalence may seem small compared to the vast majority of patients with severe HTG; however, in the absence of genetic testing, one cannot separate FCS (type I) from MCS (type V) in patients with triglycerides ≥ 1,000 mg/dL. In fact, most patients with severe HTG (97%) have a genetic basis for a heterozygous loss-of-function mutation in the *LPL* gene or its cofactors and other minor variants, or a strong component of environmental factor. There is, therefore, a polygenic basis with several possible variants in different combinations that are overrepresented among patients with severe HTG, which correspond to the multifactorial form (MCS).^32 - 37^

Surendran et al.^33^ reported that among five causal genes, 34% of identified mutations were in the *LPL* gene.^33^ Comparing the clinical and laboratory data of patients with FCS of various genetic etiologies, FCS resulting from a defect in the *LPL* gene is phenotypically very similar to that resulting from defects unrelated to the *LPL* gene. However, patients with a defect in the *LPL* gene have lower post-heparin lipase activity and tend to have higher triglycerides. Conversely, LDL-C concentrations are generally higher among people with defects in genes other than *LPL* .^38^

Using data from the National Health and Nutrition Examination Survey (NHANES) from 2001 to 2006, severe HTG was estimated to affect 5,680 adults over 20 years of age whose fasting triglyceride results were available. In these patients, the prevalence of triglycerides between 500 and 2,000 mg/dL was 1.7% (87 individuals), and levels > 2,000 mg/dL were found in only three patients.^20^ If these data were extrapolated to the North American population, they would indicate an estimated 3,357,214 adults with severe HTG (triglycerides between 500 and 2,000 mg/dL) and 81,877 with triglycerides ≥ 2,000 mg/dL.^39^

A retrospective cross-sectional study evaluated patients from Oregon Health & Science University from July 2012 to July 2017.^40^ The electronic records of patients seen during that period were reviewed based on 4 criteria: triglycerides ≥ 880 mg/dL, history of AP, absence of secondary HTG, and lack of response to triglyceride-lowering pharmacotherapy (< 20%). When 3 of 4 criteria were met, patients were considered to have likely FCS. When all 4 criteria were met, or if there was confirmation of culprit mutations by genetic testing, patients were considered to have definite FCS. Of 2,342,136 electronic records evaluated, 578 patients showed triglyceride measurements ≥ 880 mg/dL (0.025%), of whom 86 also had a history of pancreatitis. Five patients who met FCS criteria were identified and 3 of them had genetically confirmed FCS, resulting in an estimated prevalence of 1-2 per 1,000,000 people. MCS was identified in 186 patients, suggesting an estimated prevalence of 1 in 12,000 people. There were 5,181 cases of pancreatitis (0.22% of the entire cohort), 86 of which occurred in those with triglycerides ≥ 880 mg/dL (1.7% of pancreatitis cases). The rates of pancreatitis in this subsample increased to 6.5%, 100%, and 17.8% in patients with MCS, FCS, and secondary causes of HTG, respectively.^40^

In a retrospective study with data from 70,201 patients treated at the Cleveland Clinic Lipid Center from January to December 2006, 369 met the criteria of triglycerides ≥ 750 mg/dL and previous pancreatitis. Of these, 333 cases were due to secondary causes or had missing data and were excluded. Of the remaining 36 patients, 14 met criteria for FCS.^41^ In this cohort of FCS, the authors reported the prevalence to be at least 1:5,000 based on established diagnostic criteria.^22 , 42^ The prevalence reported in this study is > 20-200 times higher than the prevalence reported in previous reports. Using electronic data from the North Texas Division of the Baylor Scott & White Health System from September 2015 to September 2016, a screening of patients with triglycerides ≥ 1,000 mg/dL and a history of pancreatitis showed that, of 297,891 adult patients with available triglyceride levels, 334 (0.11%) had triglyceride levels ≥ 1,000 mg/dL and 30 (9%) of them had pancreatitis. Of these, six cases were excluded due to secondary causes. Of the 24 remaining cases, the average maximum triglyceride level was 3.085 ± 1.211 mg/dL. Thus, electronic screening of triglycerides ≥ 1,000 mg/dL and a history of pancreatitis allowed ruling out 99.99% of severe HTG cases, resulting in 24 cases in which FCS could not be excluded, suggesting a prevalence of 1 in 12,413 people. An important data limitation in both studies is the lack of genetic confirmation.^43^

Another study in Quebec evaluated plasma appearance and classified patients according to triglyceride values, probable etiology, and biochemical characteristics. A total of 354 people with lactescent plasma were compared with 482 patients with clear plasma but triglycerides > 5 mmol/L (approximately 440 mg/dL) and with 364 normolipidemic controls (triglycerides < 2 mmol/L, or < 176 mg/dL). The authors observed that lactescent plasma represented a heterogeneous group of high-risk patients among whom 28 had FCS, 62 had dysbetalipoproteinemia (due to defects in the *APOE* gene, E2E2), 182 had type IV HTG, and 82 had type V HTG. From a clinical point of view, the higher the triglyceride concentrations and the more lactescent the plasma, the greater the risk of pancreatitis. Visual examination of plasma and clinical phenotype were useful to establish the cardiometabolic risk in these patients, and identification of lactescent plasma is a simple diagnostic tool that can help identify those at increased risk.^44^

Dron et al.^36^ suggested that only 1% to 2% of patients with triglycerides ≥ 1,000 mg/dL had FCS, with the remaining majority having MCS. The authors assessed rare and common variants in two independent cohorts of 251 and 312 Caucasian patients with severe HTG. Targeted next-generation sequencing of 73 genes and 185 single nucleotide polymorphisms associated with dyslipidemia, in addition to five causal genes for FCS ( *LPL* , *APOC2* , *GPIHBP1* , *APOA5* , and *LMF1* ), was conducted. The authors found that 1.1% had biallelic rare variants, 14.4% had heterozygous rare variants, 32% had an extreme accumulation of common variants (that is, a high polygenic score), and 52% remained genetically undefined. Patients with severe HTG were 5.77 times more likely to carry one of these variants of genetic susceptibility compared with controls.^36^

A family with three members affected by FCS who had severe HTG and episodes of pancreatitis underwent genetic panel analysis.^45^ The index case was a woman with multiple episodes of pancreatitis, one of them during pregnancy, requiring plasmapheresis. The prevalence of severe HTG was also evaluated based on population data obtained from a reference laboratory where secondary causes (207,926 participants, aged 58 years, 52% women) and diabetes were ruled out. The 28-year-old woman had recurrent HTG and pancreatitis with onset at 3 months of age. Triglyceride levels were reasonably well-controlled with a low-fat diet until her early 20s, when she experienced recurrent attacks of pancreatitis and fasting triglyceride levels > 2,000 mg/dL, requiring multiple hospitalizations despite treatment. In addition to a restricted diet, she was placed on fenofibrate, niacin, medium chain triglycerides (MCTs), and omega-3 (ω3), with poor response. She became pregnant at age 30 and required weekly or biweekly plasma exchanges until delivery. Her father and sister had HTG and a history of pancreatitis. The patient was a compound heterozygote for two *LPL* mutations: c.708delA (p.G237fs*15) deletion and c.644G.A (p.G215E), which are known to impair LPL function. Her father had the c.708delA (p.G237fs*15) deletion variant, whereas her mother and sister had the c.644G.A (p.G215E) variant. Of 207,926 participants, 25 had fasting triglycerides > 2,000 mg/dL with no evidence of secondary causes, suggesting an estimated prevalence of 120/1 million people.^45^

In another study, the prevalence of FCS was assessed in a largely rural area in central New York State with an estimated population of 870,000. A review of electronic medical records from 385,000 patients identified 998 patients with triglycerides > 750 mg/dL, of whom 994 were excluded for secondary causes of HTG, satisfactory response to therapy, or lack of complete information. Four patients met criteria for FCS. Thus, the probability of finding 4 out of 870,000 would be 0.01, suggesting that the 1/1,000,000 prevalence is an underestimation. The high prevalence was attributed to a probable founder effect.^46^

The prevalence of FCS was also retrospectively assessed by reviewing the electronic medical records from 7,699,288 patients from the University of Southern California who had triglycerides > 880 mg/dL, at least one episode of pancreatitis, response to lipid-lowering therapy < 20%, and no documented secondary causes. The analysis showed an FCS prevalence of 0.26 to 0.65 per million individuals.^47^

Finally, the prevalence of FCS was determined in a quaternary care center.^48^ Data from 1,627,763 patients seen at Johns Hopkins Hospital from 2013 to 2017 were reviewed. FCS criteria included patients with a) at least one fasting triglyceride value > 750 mg/dL, b) history of AP, unexplained recurrent abdominal pain, and/or family history of HTG, and c) absence of secondary causes of HTG. Twenty-one patients with FCS and 89 with secondary HTG were identified, and FCS prevalence in this study was 13:1.000.000 (95%CI, 8-20).^48^

## 6.3. Epidemiology of Familial Chylomicronemia Syndrome in Children

There are no data regarding the prevalence of severe HTG and FCS among children. A retrospective analysis of electronic medical records from a tertiary pediatric hospital (Children’s Medical Center, Dallas) and NHANES data from 2000-2015 showed that, of 30,623 children at the Children’s Medical Center, 36 (1 in 1,000) had extremely elevated triglyceride levels (≥ 2,000 mg/dL), and one-third of them developed AP. Most of these cases corresponded to secondary causes of HTG, with an estimated prevalence of FCS of 1:6,000 in children in a tertiary care center and 1:300,000 in children in the general population. According to the 2000-2015 NHANES data, none of the 2,362 children met the criteria for severe HTG, whereas the estimated prevalence among adults was 0.02%.^49^

## 6.4. Epidemiology of Familial Chylomicronemia Syndrome in Brazil

In Brazil, case reports of FCS are very scarce. Although cases of FCS have been described in several regions of the country, with a higher concentration in regions with a founder effect (especially in the Northeast region), no publications on the subject have been found except for conference publications published in annals.

The first case report consisted of a 3-year-old boy who presented with lipemic serum and plasma triglyceride concentrations of 25,000 mg/dL at 3 months of age with exclusive breastfeeding. At 3 years of age, he developed hepatosplenomegaly and, after a diet restricted in fat and skim milk, triglycerides reached 990 mg/dL. He had zero LPL activity, and a G188E mutation was detected in exon 5 of LPL in homozygosis for him and in heterozygosis for the parents.^50^

Another report consisted of two children, one aged 21 days and the other aged 4 months and 15 days. In both cases, HTG was casually diagnosed by the xanthochromic aspect of blood during sample collection. Triglyceride levels at diagnosis were 18,019 mg/dL and 5,333 mg/dL, respectively. After in-hospital and outpatient dietary intervention, the lowest triglyceride levels achieved were 602 mg/dL and 615 mg/dL. One of the patients developed recurrent episodes of AP related to high triglyceride levels.^51^

There was a report of a 15-month-old infant from the state of Rio Grande do Norte with chylothorax and a lipid profile suggestive of FCS, with triglycerides > 1,000 mg/dL and no documented pancreatitis.^52^ Another publication reported the case of a 45-year-old woman with severe HTG, diabetes, and profuse eruptive xanthomas.^53^

Two other cases of siblings with FCS with a genetically confirmed mutation in the *LPL* gene were identified in the rural area of Paraíba.^54^ Another report consisted of a 45-day-old infant experiencing vomiting and irritability, with triglycerides of 6,541 mg/dL and altered molecular analysis in 3 variants: Chr8:19,811,733 G>A, promoting the replacement of the amino acid glycine at codon 215 by glutamate (p.Gly215Glu); Chr8:19,813,385 G>A, promoting the replacement of the amino acid arginine in codon 270 by histidine (p.Arg270His); and Chr8:19,811,823 T>C, promoting the replacement of the amino acid isoleucine at codon 245 by threonine (p.Ile245Thr). Dietary behavior consisted of skim milk, MCTs, and vitamins A, D, E, and K. After the patient was discharged, the diet was changed to include infant formula, which led to an increase in triglycerides (11,760 mg/dL). The patient underwent fasting and the previous dietary behavior was subsequently restored, which allowed reasonable control of triglyceridemia, adequate growth, and weight gain.^55^

Lima et al.^56^ recently reported 12 cases of FCS in patients with a homozygous mutation in the intronic region of the *GPIHBP1* gene, all with severe HTG (2,351 mg/dL [885-20,600 mg/dL]) and low HDL-C (18 mg/dL [5-41 mg/dL]) and 33% with episodes of AP. All patients were from cities in the Northeast of the country, suggesting a founder effect.^56^

Data on the prevalence of FCS varies greatly between studies due to the lack of standardized clinical criteria, the similarity with MCS, the scarcity of tests for genetic confirmation, the lack of national and international registries, and the founder effect of causal genes.

## 7. Clinical manifestations of familial chylomicronemia syndrome, differential diagnosis, and management of complications

### 7.1. Clinical Manifestations in Familial Chylomicronemia Syndrome

Clinical manifestations of monogenic forms of chylomicronemia usually happen during childhood or in the beginning of adult life. However, as this is a relatively rare disease, diagnostic delays are common and lead to diagnosis in adult life, when complications are already established.^2^

A review of the APPROACH study database demonstrated that the mean age at diagnosis was 24 years; more than half of the 66 patients were diagnosed after 20 years old. At diagnosis, 75% of patients had already presented an episode of pancreatitis.^57^ Other series describe a mean assessment by five different medical professionals before reaching a diagnosis.^58^

These data reinforce the importance of early and timely diagnosis. The main clinical manifestations of FCS are described in the following sections.

#### 7.1.1. Hypertriglyceridemia

On laboratory assessment, the affected patients presented hyperchylomicronemia, with sharp increases in triglycerides – in general, between 1,500 and 5,000 mg/dL – at the expense of increased VLDL cholesterol (VLDL-C) and especially circulating chylomicrons. Since a small amount of cholesterol is also transported by and is present in chylomicrons, total cholesterol may be increased, usually in triglycerides: cholesterol ratio > 5:1. Many patients have a moderate increase in VLDL-C, with LDL-C and ApoB levels < 100 mg/dL.^21^

According to the Fredrickson Classification, although type V phenotype is more common, type I seems to be more specific for the diagnosis of FCS in adults. In children, type I phenotype is more frequently observed.^21^

Severe HTG in patients with FCS usually presents a poor response to fibrates and/or other lipid-lowering drugs. In these cases, which comprise a huge challenge to clinical practice, the main therapeutic alternative is a diet with a drastic reduction in fat intake (8% to 10% of the daily calory intake). The strictness of this diet not rarely makes adhesion to long-term treatment difficult and also significantly affects the patients’ quality of life.^5^

#### 7.1.2. Abdominal Pain and Acute Pancreatitis

Recurrent abdominal pain is present in up to 50% of patients, is not necessarily associated with AP, and can be debilitating.^27^

When triglyceride levels are > 1,000 mg/dL, the risk of pancreatitis increases in 3% for every 100 mg/dL.^59^

A Canadian study compared a group of 25 individuals with FCS to a group of 36 patients with MCS and demonstrated that, despite presenting similar mean triglyceride levels, the group with FCS presented a 10-fold higher risk (60% vs 6%) of pancreatitis.^22^ This is probably due to a longer duration of exposure to hyperchylomicronemia, which in FCS tends to happen in the first years of life.

Multiple episodes of AP and the severity of dietary restrictions negatively affect the patient’s quality of life and considerably increase morbidity and mortality by the disease. Recurrent pancreatitis occurs in 50% of patients with FCS; the overall associated mortality rate is of 5% to 6% and can reach 30% in subgroups of patients who progress to pancreatic necrosis or persistent multiple organ failure.^59^

#### 7.1.3. Neurological Manifestations

Fatigue, mental confusion, irritability, and cognitive deficit – described as “mental fog” – are the most described symptoms among patients with FCS.^57 , 58^

#### 7.1.4. Hepatosplenomegaly

Hepatosplenomegaly is one of the findings that are reversible with treatment and results from excess chylomicrons in macrophages of the reticuloendothelial system in FCS.^57^

#### 7.1.5. Eruptive Xanthomas

Xanthomas correspond to yellow, eruptive skin lesions with an erythematous halo measuring around 2 to 5 mm diameter. These are found on extensor surfaces (elbows and knees) and on the buttocks. Their prevalence is low (affecting 17% to 33% of the patients), and they are not always correlated with episodes of pancreatitis.^27^

#### 7.1.6. Lipemia Retinalis

Milky white appearance of the blood in retinal vessels on fundus examination, which can be seen in up to 30% of the patients and is correlated with higher levels of triglycerides.^57^

#### 7.1.7. Quality of Life

The IN-FOCUS study, with 166 patients with FCS, showed an important impact of the disease on quality of life. Hospitalization rates can interfere with social conditions and job possibilities, and more than 22% of patients reported depression or anxiety related to the pain or pancreatitis episodes.^58^

#### 7.1.8. Diagnostic Score

Some scales or scores that consider clinical manifestations have been proposed for diagnosing FCS; however, they need to be validated in larger samples of populations with severe HTG.^21^ Additionally, their applicability is questionable because they include previous pancreatitis episodes among their scoring criteria.^59^ Fundamentally, the aim of using diagnostic scores consists in screening asymptomatic patients and preventing complications such as AP. The assessment of databases with a larger number of patients with FCS and further detailing of clinical forms should contribute to the elaboration of criteria with higher sensitivity and specificity for diagnosing FCS.

The most widely used score is that by Moulin et al.,^21^ which uses as selection criteria the presence of severe HTG (> 1,000 mg/dL of fasting triglycerides and outside of the acute phase) and attributes points to increased triglyceride levels once secondary causes are ruled out, to a history of pancreatitis, recurrent abdominal pain, unresponsiveness to usual triglyceride-lowering treatment, and age of symptom onset ( Chart 1 ). This score was tested in cohorts of patients with genetic confirmation of FCS and MCS, being validated in other cohorts showing an area under the curve of 0.91. This position statement recommends its use when screening for genetic testing.

**Chart 1:** – Clinical score for suspecting FCS

Criterium	Score
Fasting triglycerides ≥ 1,000 mg/dL in 3 not necessarily consecutive analyses	+ 5
* Fasting triglycerides at least once ≥ 1,760 mg/dL	+ 2
Previous triglycerides < 200 mg/dL	- 5
No secondary factors, except for pregnancy and ethinylestradiol	+ 2
History of pancreatitis	+ 1
Unexplained recurrent abdominal pain	+ 1
No history of familial combined hyperlipidemia	+ 1
No response to lipid-lowering treatment (triglyceride decrease < 20%)	+ 1
Symptom onset at age:	
< 40 years	+ 1
< 20 years	+ 2
< 10 years	+ 3
FCS score	
Very likely	≥ 10
Unlikely	≤ 9
Very unlikely	≤ 8

FCS: familial chylomicronemia syndrome. * Only if the previous criterium (fasting triglycerides ≥ 1,000 mg/dL in 3 not necessarily consecutive analyses) is positive. Adapted from Moulin et al. Atherosclerosis 2018; 2018;275:265-272. ^*21*^

## 7.2. Differential Diagnosis

### 7.2.1. Multifactorial Chylomicronemia Syndrome

In adults, the main differential diagnosis for FCS is MCS. Previously named Fredrickson type V hyperlipoproteinemia or severe polygenic HTG, multifactorial HTG is a polygenic disorder that includes rare heterozygous variants in the five FCS genes or variants commonly associated with HTG, being worsened by the presence of comorbidities or secondary causes of increased triglycerides such as uncontrolled diabetes, hypothyroidism, obesity, and metabolic syndrome.^22^ Dietary factors, such as alcohol abuse and diets high in fats, simple sugars, and other carbohydrates with high glycemic index are common causes of HTG exacerbation. Among environmental causes, we highlight the use of certain medications (glucocorticoids, oral estrogens, thiazide diuretics, noncardioselective beta blockers, second-generation antipsychotics, protease inhibitors, cyclophosphamide, bile acid sequestrants, amiodarone, retinoic acid, isotretinoin, sirolimus, L-asparaginase, and immunosuppressants such as interferon and cyclosporine) and physiological conditions such as pregnancy, especially in the third trimester.^22^ The prevalence of MCS usually tends to grow linearly with the increase in prevalence of the most common secondary causes (obesity, metabolic syndrome, and type 2 diabetes). Among patients with MCS, chylomicronemia fluctuates and is manifested in later stages of life when compared to FCS. Additionally, MCS tends to have a better therapeutic response to lifestyle changes and treatment of secondary factors, as well as triglyceride-lowering pharmacotherapies. MCS is characterized by an increased risk of pancreatitis, albeit lower than that reported in patients with FCS.^22 , 28 , 60^

### 7.2.2. Lipodystrophies

Other relevant differential diagnoses for FCS are lipodystrophies, which are a heterogeneous group of diseases characterized by the selective loss of adipose tissue that can progress with severe HTG and pancreatitis. Lipodystrophies can be inherited or acquired and are classified as generalized or partial as to their extension; partial forms associated with HIV infection are the most common ones. Inherited lipodystrophies are rare disorders that can manifest at birth or display loss of fat in later stages of life. These conditions still represent a diagnostic challenge, especially considering the partial forms, whose diagnostic suspicion should be raised in the presence of moderate to severe HTG associated with a thigh skinfold measurement < 22 mm in women or < 10 mm in men, and/or cases of diabetes that require subcutaneous insulin in daily doses > 2 IU/kg.^61^

## 7.3. Managing Complications of Familial Chylomicronemia Syndrome

### 7.3.1. Acute Pancreatitis

AP is a relatively frequent event, with different causes that include HTG. Identifying the specific cause is fundamental for establishing treatment and preventing future episodes. In various case series, cholelithiasis is the main cause, followed by alcohol consumption and HTG (less than 10%).^63^ Despite being a less frequent cause, increased triglyceride levels in patients with pancreatitis are associated with higher mortality and a worse prognosis.^64^ During pregnancy, estrogen stimulates liver VLDL production and reduces the removal of triglycerides by LPL in the liver and fatty tissue, which makes HTG the most frequent cause of AP.^66^

Episodes of pancreatitis due to HTG usually occur with triglyceride levels > 1,000 mg/dL.^67^ The risk and disease severity increase even further in patients with levels > 2,000 mg/mL.^68^ This happens regardless of whether the basic cause of HTG is primary (genetic) or secondary. However, genetic causes usually come with higher triglyceride levels and, consequently, a higher risk of pancreatitis.

According to the Fredrickson classification, type I (chylomicron), IV (VLDL), and V (chylomicron and VLDL) present HTG, of which FCS (type I) has higher levels and can lead to pancreatitis regardless of triggering factors (decompensated diabetes, obesity, use of corticosteroids, estrogens, or other drugs that cause HTG).

The mechanism causing pancreatitis is not completely understood, but triglycerides themselves do not seem to act directly in the pancreas. The accumulation of free fatty acids in the pancreatic cells happens in the presence of pancreatic lipase and triggers cell injury and pancreatic inflammation.^70^ Another potential mechanism stems from the accumulation of glutamic acid decarboxylase (GAD). In the absence of LPL activity and consequent accumulation of chylomicrons, there is also an increase in GAD that triggers TNF-alpha- and IL-6-mediated inflammation. Chylomicrons themselves can also obstruct distal pancreatic blood flow and cause ischemia.

The clinical presentation of pancreatitis is similar regardless of its etiology. Patients with FCS not rarely present recurrent pancreatitis episodes, and some of them report an unknown number during anamnesis. This triggers psychological changes, compromising quality of life.^58^ Some patients even avoid going to parties and gatherings, because they fear eating could trigger pancreatitis. Children require constant vigilance, as they do not fully understand the disease and want to eat just as their colleagues who do not have the disease. After a first pancreatitis episode (many times during adolescence, after menarche), the acute pain and need for hospitalization are factors that motivate a more rigorous adherence to the restrictive diet required for controlling severe HTG.

Eruptive xanthomas are infrequent even in severe HTG. However, when present in a patient with AP, these lesions suggest HTG as etiology. Extensor surfaces of the arms and legs are the most frequent sites. Fatty infiltration in the liver and spleen can occur, leading to hepatosplenomegaly, but these are unspecific findings.

The diagnosis of AP should begin with a clinical suspicion (acute and persistent abdominal pain, irradiating to the dorsal region), confirmed by laboratory examinations (amylase or lipase values three times the upper normal limits) and imaging tests (ultrasonography, computed tomography, or magnetic resonance imaging). At least 2 of these 3 findings should be present for diagnostic confirmation, and this does not depend on the etiology of pancreatitis. Not rarely, patients present only abdominal pain, without laboratory or imaging alterations. In the absence of a suggestive clinical picture, lipase and amylase analyses may be more of a hindrance than a help. Triglyceride levels < 1,000 mg/dL during a clinical episode suggesting pancreatitis leave HTG as an unlikely cause of pancreatitis.^67^

Once diagnosis is confirmed, treatment should aim to reduce/relieve pain and maintain adequate hydration; even though oral nutrition is suspended, adequate nutrition should be provided to a patient in an acute setting as early as possible. Reducing triglyceride levels is fundamental for reversing the inflammatory process, and considering this happens through chylomicrons, greater reduction should be achieved when interrupting oral nutrition. In the most severe cases (body temperature > 38.5ºC or < 35.0; heart rate > 90 bpm; respiratory rate > 20/min or pCO_2_< 32 mmHg; leukocytes > 12,000 or < 4,000/mL), requiring a faster reduction of triglyceride levels, plasmapheresis may be used. In case an adequate diet is not instituted or triggering factors are not controlled, achieving remission becomes more difficult. Insulin stimulates LPL and can also be used in some cases (regular insulin, 0.1 to 0.3 U/kg/h). Similarly, heparin also acts by stimulating LPL, but its use should be carefully considered because it may not bring benefits in the medium term (increases risk of bleeding and release of toxic components of triglycerides).^63^

Once the patient is clear of AP, the factor that triggered the inflammatory process should be assessed and treated. Maintaining adequate weight, exercising regularly, and avoiding medications or other triggering factors for HTG help prevent new episodes of pancreatitis.

Differently from other HTG causes that respond well to fibrates, FCS does not present a significant reduction of triglyceride levels with these drugs, which are not used to prevent pancreatitis in these patients. ApoC3 is an inhibitor of LPL; its inhibition by volanesorsen (an antisense oligonucleotide against ApoC3) used once a week significantly reduced (77%) triglyceride levels and, consequently, the chances of pancreatitis.^23^ From a pathophysiological point of view and considering the benefits demonstrated in clinical studies, patients with FCS benefited from the use of volanesorsen. However, patients with frequent pancreatitis (usually one or more episodes a year) and difficulties controlling triglyceride levels with usual diet treatment would benefit from it the most.

## 8. Laboratory Diagnosis of Familial Chylomicronemia Syndrome

Clinical laboratories have a supporting role in the diagnosis of FCS. Lactescent (milky white) serum is the main indicator of the presence of chylomicrons and follows high triglyceride levels. Some aspects should be considered for the effective laboratory diagnosis of FCS. The phases responsible for the result of a laboratory analysis comprising an FCS investigation are: pre-analytical, analytical, and post-analytical.

### 8.1. Pre-analytical Phase (Patient Instructions)

#### 8.1.1. Collection Instructions

Fasting is no longer mandatory for lipid panels; however, in situations such as triglyceride metabolism disorders, it is required for diagnostic confirmation of FCS. In these cases, adults over 20 years old should fast for 12 hours.^2 , 71 - 73^ In children, this duration varies according to the age group. Infants (up to 1 year) should fast for 3 hours or collection should be done immediately before the next feeding; non-infants (2 to 5 years) should fast for 6 hours. Children over 5 years old and adolescents should fast for 12 hours.

#### 8.1.2. Pre-analytical Causes of Interference in Triglyceride Analyses

The preparation for sample collection considering triglyceride analyses in adults (> 20 years) consists in the patient’s usual diet, with 12 hours of fasting; alcohol consumption should be avoided at least 72 hours before the test; and no strenuous exercise should be performed 24 hours before the test.^74^

Some situations increase the free glycerol blood level, leading to an overestimation of triglyceride levels with no serum cloudiness. In these cases, the patient should be evaluated for one of the events described in the literature: recent physical exercise, alcohol consumption, acute liver disease, decompensated diabetes, parenteral nutrition, or intravenous glycerol-containing drugs.^74^

#### 8.1.3. Pre-analytical Phase (Laboratory Instructions)

In HTG, the serum varies from cloudy to lactescent. Grade 1 – slightly cloudy; Grade 2 – cloudy; Grade 3 – very cloudy; Grade 4 – lactescent. Since serum appearance is a subjective issue, only after measuring triglyceride levels and storing the serum for 12 hours under refrigeration that we can proceed with visual inspection.^74^

## 8.2. Analytical Phase

### 8.2.1. Methodologies Assessing Chylomicrons

Methodologies that can be used to indicate the presence of chylomicrons in serum are demonstrated in the following paragraphs.

***8.2.1.1. Ultracentrifugation***

The gold standard for separating fractions of lipoproteins according to their lipid content and density. However, this method has inherent limitations that include its lack of availability at clinical laboratories, high cost, and delays in executing the technique, becoming unfeasible for Brazilian laboratories.

***8.2.1.2. Serum Appearance***

For observing chylomicrons in the lactescent serum, we recommend the use of whole blood collection tubes with serum-separating devices for centrifugating and collecting the serum from the supernatant.^75^ When this is not possible, after centrifuging and removing samples for laboratory analyses, 1 mL of serum should be transferred to a transparent disposable hemolysis tube. The lactescent serum obtained in any situation should sit in a refrigerator for 12 hours so that a creamy layer is observed on its surface, indicating the presence of chylomicrons, which should be specified in the patient’s report.^74^

***8.2.1.3. Lipoprotein Electrophoresis***

Lipoprotein electrophoresis, also named lipidogram, can help confirm the presence of chylomicrons with a colorful band at the site of sample application.^74 - 76^ However, this method of separating serum lipid fractions is no longer used in routine clinical practice because it is semiquantitative and cholesterol fractions were adopted as risk markers for cardiovascular disease; therefore, we do not recommend the use of this methodology in this document.

Out of the three mentioned methodologies, the most widely accessible at various laboratories is serum appearance, which is the one recommended by this document.

### 8.2.2. Methodologies for Assessing Triglycerides

The methodology for measuring triglycerides can use an enzymatic colorimetric reaction and/or ultraviolet (UV) detection. These methods are precise and inexpensive. The reaction begins with the hydrolysis of triglycerides into three fatty acids and one glycerol molecule.^75^ Therefore, for each triglyceride molecule, one glycerol molecule will react and provide the triglyceride concentration in the sample. Any physiological situation that increases serum glycerol levels will overestimate triglyceride levels. The literature describes a rare genetic disease, glycerol kinase deficiency, also named pseudohypertriglyceridemia; this disorder causes hyperglycerolemia and HTG without the lipemic appearance of the serum.^77^

### 8.2.3. Interferences to Triglyceride Results

Lipemia, depending on its intensity, leads to falsely increased triglyceride levels due to the association between the method coloration and serum cloudiness. In this case, the sample should be diluted in buffered saline solution (pH 7.4) or in the automation diluent (platform-dependent) for obtaining a reliable result.^75^

Serum dilution may follow a scale according to triglyceride levels and the method’s analytical range. For example, if the analytical range is 8 to 885 mg/dL, the following dilutions may be suggested: 1:4 (triglycerides 400-600), 1:6 (triglycerides 601-1,000), 1:10 (triglycerides 1,001-2,000), or 1:20 (triglycerides ≥ 2,001).

FUNDAMENTAL: Even after diluting the sample, the obtained results should be kept in the dynamic range; this is essential for maintaining the method’s linearity and reproducibility.

IMPORTANT: Using a sample blank (diluted sample) for considering cloudiness even after the dilution. The difference (delta) between reads should be used = diluted sample - diluted sample blank, multiplying the delta value by the dilution rate; only then the value should be associated with control and/or platform calibrator samples.

EXAMPLE: If the result of a diluted (1:4) sample was 250 mg/dL, it should be multiplied by 4, and the result will be 1,000 mg/dL triglycerides. However, if the sample blank reads 50 mg/dL, this value should be subtracted from the diluted (1:4) sample (250 - 50 = 200); this value is then multiplied by 4 and the result will be 800 mg/dL triglycerides. Therefore, it is essential to account for the cloudiness of the diluted serum. The greater the dilution, the higher the overestimation of triglyceride levels in case the sample blank is not accounted for.

Therefore, the methodology’s technical description must be analyzed for obtaining information and instructions, such as its analytical range (dynamic range), possible dilutions that can be done, the diluent material, use of sample blank, or even changes to the automated program. These descriptions are method-platform-, and manufacturer-dependent, and should be followed according to their respective information.^75^

### 8.2.4. Interferences of Triglycerides to Other Analytes

***8.2.4.1. LDL-C***

The laboratory analysis of LDL-C is hindered by increased triglyceride levels in the lipemic serum. LDL-C calculation via the commonly used Friedewald formula not only is limited to patients with triglyceride levels up to 400 mg/dL but can also be underestimated, and the patient ends up not receiving treatment due to triglyceride interference. On the other hand, Martin’s equation applies correction factors to the Friedewald formula, allowing a more reliable estimation of LDL-C, and can be applied with triglyceride levels up to 13,975 mg/dL. In addition, the direct method can be used to measure LDL-C but will present limitations depending on the degree of lipemia.^2 , 71 - 73^

In FCS or in MCS, HTG is severe due to the presence of chylomicrons, VLDL, and their remaining components. The patient presents a reduction in VLDL lipoprotein lipolysis, which leads to a decrease in LDL lipoprotein production in the plasma and a high amount of large and triglyceride-rich particles (chylomicrons and VLDL) when compared to a normal individual. In this case, no matter the methodology (calculated or directly measured LDL-C), the values are always lower than the method’s analytical sensitivity. We recommend that laboratories release LDL-C results that are extremely low or negative as < 10 mg/dL.^73^

***8.2.4.2. Platelets***

Platelet counts in automated hematology are performed by using impedance and, in case of lipemia, interferences will possibly decrease their count. The same association happens when determining hematocrit – in this case, with an important piece of information, and the results are calculated from the association between hemoglobin and erythrocyte count.^75^

***8.2.4.3. Analytes with Colorimetric Analysis***

Methods with colorimetric endpoint readings usually present more restrictions regarding lipemia. This can also happen less intensely in systems with UV detection. This interference is directly proportional to serum cloudiness but is not always proportional to triglyceride concentration. It should be noted that lipoproteins have different sizes and are constituted by different percentages of triglycerides.^75^

***8.2.4.4. Enzymes***

Kinetic, colorimetric, and/or UV enzymatic reactions can suffer interferences by lipemia, depending on its intensity. Therefore, alkaline phosphatase and gamma-glutamyl transferase have greater limitations, because they employ p-nitrophenyl phosphate in their assays (colorimetric methods). However, the use of exclusively UV-based methods can also face restrictions with lipemia.^75^

***8.2.4.5. Electrolytes***

When determining sodium in the serum and/or plasma with increased triglyceride levels, results will be falsely low. In this case, the sodium value can be corrected by calculating: triglycerides (g/dL) x 4 - 0.60 = percentage factor.^75^

Example: Na^+^122 mmol/L and triglycerides 2,100 mg/dL; 2.10×4−0.60=7.8%


$$\left.{Na}^{+}122\times 7.8\%=131.5mmol/L\text{(corrected}{Na}^{+}\right)$$

### 8.2.5. Laboratory Analyses for Differential Diagnosis

***8.2.5.1. Post-heparin LPL Activity***

LPL activity is not measured in laboratory routine analysis, but it may be useful when screening for genetic testing for FCS. When the laboratory allows the LPL activity assay to be performed before and 10 minutes after heparin injection (IV heparin [50IU/kg]), whole blood should be collected from the other arm using a heparinized tube and transported on ice to the laboratory. The collection tube should be centrifuged for 10 minutes at 3,000 rpm and 4ºC, and plasma should be immediately separated. The tube with plasma should be stored at -80ºC until the day of analysis using the adopted protocol or sent to a specialized laboratory.

LPL activity is drastically decreased in FCS by homozygous genetic alterations to LPL, and it is frequently reduced when these alterations occur in LPL cofactors ( *APOC2* , *APOA5* , *GPIHBP1* , and *LMF1* genes) in cases of homozygosity or compound heterozygosity. However, researchers have demonstrated that the discriminating capacity of this test for identifying patients with common variants of LPL genes is limited, which justifies the absence of a recommendation in this document.^79^

***8.2.5.2. Plasma ApoC3 Measurement***

Increased plasma levels of ApoC3 are an important risk factor for HTG. Recent studies concluded that ApoC3 also inhibits an LPL-independent triglyceride-rich lipoprotein pathway. ApoC3 measurement is feasible at large clinical laboratories or support laboratories.^71^

## 8.3. Post-analytical Phase

### 8.3.1. Recommendations for NOTES in Laboratory Reports21,23

- In adults, in case of triglyceride levels > 1,000 mg/dL assessed after 12 hours of fasting, in 3 different blood collections and once secondary causes of HTG are ruled out, the diagnosis of hyperchylomicronemia should be considered.
- In children and adolescents, in case of triglyceride levels > 880 mg/dL regardless of fasting, in 3 different blood collections and once secondary causes of HTG are ruled out, the diagnosis of hyperchylomicronemia should be considered.
- In children or adults, a triglyceride level < 170 mg/dL EXCLUDES the investigation of hyperchylomicronemia.

Recommendation: Adults should maintain normal diets, fast for 12 hours, avoid alcohol (72 hours) and exercise (24 hours). For children, fasting periods vary according to the age group. Infants (up to 1 year) should fast for 3 hours or collection should be done immediately before the next feed; non-infants (2 to 5 years) should fast for 6 hours. Children over 5 years old and adolescents should fast for 12 hours. Excess free glycerol in the blood leads to an overestimation of triglyceride levels. Lactescent serum should sit in the fridge for 12 hours for verifying the presence of chylomicrons. When measuring triglycerides, the analytical range, dilution rate, diluent material, and use of sample blank or changes to automation should be kept in mind. In severe HTG, FCS, or MCS, LDL-C levels (calculated or directly measured) that are too low or negative should be reported as < 10 mg/dL. Lipemia, depending on its intensity, interferes with platelet counts, colorimetric methods, enzymatic reactions (kinetic, colorimetric, and/or UV), and sodium determination. The post-heparin LPL activity assay is not recommended in this document. ApoC3 measurement is viable at clinical laboratories. We recommend that laboratory reports mention that an FCS diagnosis, after ruling out the secondary causes of HTG, should be considered in the following situations: 1) Adults with 12 hours of fasting and triglyceride levels > 1,000 mg/dL, in 3 different collections; 2) children and adolescents with triglyceride levels > 880 mg/dL, regardless of fasting, in 3 different collections; 3) in children and adults, a triglyceride level < 170 mg/dL EXCLUDES the investigation of hyperchylomicronemia. (Grade 1 Recommendation, Level of Evidence C).

## 9. Genetic Counseling and Stages of Diagnosis and Follow-up of Severe Hypertriglyceridemia

The American Society of Human Genetics defines genetic counseling as a communication process that handles human problems associated with the occurrence and risk of occurrence or recurrence of a certain genetic disease in a family.^80^

The term genetic counseling was used for the first time in 1947 by Sheldon Reed,^81^ as a way of, in a world post-World War II, face the eugenic concepts that permeated the scientific and medical societies as to genetic diseases and disabilities in general. Since then, it incorporated the principles of the psychosocial model of patient care, using as foundation the empathy and human skills involving communication, recognizing the grieving process, and self-defense mechanisms. The professional should use ethical neutrality and nondirectivity – two fundamental principles of genetic counseling – for guiding the patient and the family, providing answers and information as completely as possible so that the individual seeking guidance can make his or her decisions, being aware of the risks and alternatives.

The term “aconselhamento,” used in the Portuguese translation, actually does not indicate the true objective of this process, because the etymology of the verb indicates “to give advice,” when in reality, this is not the goal of this procedure. The closest Portuguese translation for genetic counseling^82^ would be “consultoria genética”: its goal is to provide guidance so that the patient feels secure when making decisions, understanding that there is no right or wrong and no conduct should be suggested. That being said, it is important to understand that, when performing genetic counseling, the professional should respect the family’s ethical and religious values, following the three principles that govern medical ethics: autonomy, beneficence, and nonmaleficence.^83^

It is worth noting that what many call genetic counseling is just a stage of the whole process.^84^ Genetic counseling involves, in total, five phases:

1. Establishing and/or confirming diagnosis, which involves anamnesis, physical examination, elaboration of a diagnostic hypothesis, request and interpretation of complementary examinations; this could last for weeks, months, or years until diagnosis is achieved.
2. Calculating genetic risk: a more theoretical phase that is many times separated from the family; it aims to calculate the risk of occurrence or recurrence of a certain genetic condition. The condition’s etiology can be monogenic, chromosomal, multifactorial, or even unknown. For each situation, a different risk can be calculated, thus disease etiology is fundamental for establishing risk as precisely as possible.
3. Communication; in this phase, patients receive guidance regarding the risks, many times involving conversations about therapeutic and prognostic options. The combination between phases 2 and 3 represents what is commonly referred to as genetic counseling.
4. Decisions and Action: phase that involves helping the family and the patient with the decisions taken in the Communication phase, regarding both the treatment and possible contraceptive methods.
5. Follow-up, representing a continuous phase where the patient or the family are followed up, observing their individual needs and the natural history of the genetic condition.

It is important to note that some stages of genetic counseling involve medical conducts, whereas others can be performed by various health professionals, as long as they are properly trained on the previously mentioned communication abilities and human and medical genetics.^84^

The two phases that represent genetic counseling the most are undoubtedly those of genetic risk calculation and communication. Although apparently simple, defining the risk of occurrence or recurrence of a certain genetic condition involves broad knowledge of the basis of genetics and inheritance. Talking about a risk of recurrence compatible with autosomal dominant or recessive inheritance seems simple when considering Mendel’s laws of genetic inheritance, but some confounding factors should be kept in mind, such as incomplete penetrance, variable expression, mosaicism, or genetic heterogeneity. Each of these factors can interfere with the clinical diagnosis of different forms of the disease, making a challenge out of adequately guiding patients through the risks.

The confirmation of a pathogenic variant explaining a phenotype can be fundamental for considering the correct risk in these cases. It is also important to note that different inheritance patterns (apart from mendelian inheritance) can present risks that require a more complex estimation. For example, in multifactorial risk, one should consider the number of affected individuals in a family, relationship with the proband, and factors that may vary from case to case, such as the age of symptom onset, symptom severity, and environmental factors. In a context of multifactorial inheritance, identifying these risks and considering how much they affect the total risk may be completely impossible, thus we consider a risk of recurrence that is always approximate or empirical, considering the whole empirical knowledge and risks of recurrence calculated based on population-based studies.^84^

This way, in order to talk about risk of occurrence or recurrence, a clear definition between FCS^20^ or MCS^36^ should be established.

FCS is inherited in an autosomal recessive pattern, that is, an individual needs a homozygous variant or two variants in compound heterozygosity, both of which pathogenic or probably pathogenic, in order to present the phenotype.

This pattern of autosomal recessive inheritance, by biallelic homozygous mutation (same mutation in both copies) or compound heterozygosity (one mutation in each copy), is present both in cases of LPL and other genes involved with the monogenic forms: *APOC2* , *APOA5* , *GPIHBP1,* and *LMF1* .^86^

It is known that the progenitors of an individual with FCS will each have a copy of the affected variant. This way, the siblings of a person with FCS have 25% of risk of also inheriting the syndrome. Finally, an individual with FCS will always pass one of the variants to his or her children. In case the person’s partner also has a variant of the same gene, the risk to their children from this combination is 50%.^18^

Since the HTG phenotype can also be caused by the presence of common or rare functional variants of genes that increase triglyceride levels, making up a polygenic inheritance pattern, molecular diagnosis is required for proper genetic counseling.^86^

The chances of other family members also presenting FCS will depend on their family history; therefore, a pedigree should always be considered to help with risk calculation. It is important to mention that, although individuals with a heterozygous pathogenic variant may present increased triglyceride levels, individual triglyceride measurement should not be used for considering a carrier status, as individuals with a heterozygous variant may present normal levels of triglycerides while individuals without the variant may present variations in triglyceride levels due to environmental factors.^86^

Only 1% of HTG cases present biallelic mutations. On the other hand, 14% of patients with HTG are estimated to carry rare heterozygous mutations, a rate 3 to 5 times higher than that the general population. The use of polygenic risk scores may be useful for identifying these individuals.^20 , 38 , 87^

The indication of genetic testing evaluates aspects (eg, positive family history, presence of a recognized inheritance pattern in the family, absence of secondary factors, relatively young patient age, and important biochemical alterations) that help interpret test results and consequently help with genetic counseling regarding the risk of family recurrence.^20 , 38 , 87^

## 10. Nutritional Guidance for Chylomicronemia in Adults, Children, and Adolescents

The treatment of FCS is based on severe dietary fat restriction^88^ to prevent the synthesis of chylomicrons, particles formed exclusively in the enterocyte which are responsible for the transport of dietary fat and cholesterol.^89 , 90^ Patients with FCS have mutations associated with LPL or its cofactors, which compromise the hydrolysis of dietary triglycerides.^21 , 91^ For this reason, a fat-restricted diet is recommended, limiting fat to a maximum of 10% of daily total energy intake (TEI) (15 to 20 g of fat per day).^92^

Because of the severity of the disease, patients should be very well informed of the importance of strictly following the guidelines, and the nutritionist should indicate food options that contribute to greater adherence to treatment. Some authors consider FCS to be a devastating condition for patients and a frustrating one for physicians and nutritionists regarding the main treatment target – controlling severe HTG.^25^ FCS adversely impacts patients’ quality of life because of the difficulty in following a strictly restricted diet, which significantly compromises social interaction.^24^ Limited knowledge of FCS prevents friends and family from apprehending the seriousness of the disease. The IN-FOCUS study,^93^ involving 166 patients with FCS from 10 countries, showed that more than 90% of the participants found following a strict diet to be difficult. Database evaluation of a subgroup (n = 60) of participants from the same study^58^ showed that 22% of the participants reported anxiety, fear, and worry about the quantity and quality of food to be consumed, especially in social and work situations. These symptoms were experienced at least once a month, or several times a week.

The health care team can use motivational interviews to help patients with FCS resolve their internal conflicts and to promote greater adherence to a fat-restricted diet.^92^ This may help patients develop their own eating plan with the assistance of a nutritionist and accept their personal responsibility for self-management of the disease.

Although fat restriction is the mainstay of FCS treatment, patients should also avoid foods with added sugars, such as sucrose and corn syrup,^92^ because they induce hepatic lipogenic pathways. Patients should also abstain from consuming alcohol, because its consumption is linearly associated with plasma triglyceride levels.^91^ In addition, monitoring dietary intake of fat-soluble vitamins, minerals, and essential fatty acids is recommended, and their supplementation may be necessary.^94 , 95^ Specifically concerning dietary fat, it is essential to consider fatty acid type and chain length, because they are absorbed differently and can influence the plasma concentration of triglycerides and the production of chylomicrons.

### 10.1. Fatty Acid Classification and Absorption

Saturated fatty acids (SFAs) are classified according to chain length of the carboxylic acid into short-, medium-, or long-chain, and these characteristics influence their absorption process. Short-chain fatty acids include acetate (C2:0), propionate (C3:0), and butyrate (C4:0), whereas medium-chain fatty acids include caproic (C6:0), caprylic (C8:0), and capric (C10:0) acids. Long-chain fatty acids contain more than 12 carbons and include lauric (C12:0), myristic (C14:0), palmitic (C16:0), and stearic (C18:0) acids.^96^ Short-chain fatty acids are produced by colonic bacterial fermentation, whereas medium-chain fatty acids are found in coconut and palm oils.^96 , 97^ Coconut fat is the main dietary source of lauric acid, which is found in minute amounts in other foods. Palmitic acid is the most abundant fatty acid in the diet, and the main sources are red meat and palm oil. Because palm oil is a structurally stable fat, it has been widely used in the food industry.^98^ The main sources of myristic acid are coconut fat, milk, and dairy products, whereas the main source of stearic acid is cocoa.^99^ All unsaturated fatty acids have a long chain and are classified as monounsaturated (MUFAs) or polyunsaturated fatty acids (PUFAs). The main MUFAs are palmitoleic (C16:1 ω7) and oleic (18:1, ω9) acids, with a single double bond in their structure.^100 , 101^ The main dietary source of palmitoleic acid is macadamia, whereas oleic acid is found mainly in olive and canola oils, and also in nuts such as peanuts, hazelnuts, macadamia nuts, almonds, and cashew nuts.^102^ They are also present in beef, chicken, and pork fats, accounting for 40% to 50% of total fat content in these foods.^103 , 104^

PUFAs contain two or more double bonds and are part of the omega-6 (ω6) or ω3 series depending on the position of the first double bond counted from the methyl end of the carbon chain. Fatty acids of the ω6 series are represented by linoleic acid (C18:2), whose main sources are vegetable oils (sunflower, corn, and soybean oils), walnuts, and chestnuts. The ω6 series also includes arachidonic acid (C20:4), which is synthesized endogenously from linoleic acid by enzymatic activity. Alpha-linolenic acid (ALA [C18:3]), of the ω3 series, is obtained from vegetable oils, mainly canola and soybean oils, and also from flaxseeds and chia seeds.^105^ The ω3 fatty acids of animal origin are eicosapentaenoic acid (EPA [C20:5]) and docosahexaenoic acid (DHA [C22:6]), found in the oils of fish and crustaceans mainly from cold- and deep-water habitats.^106 - 108^ Linoleic and linolenic acids are considered essential fatty acids because they are not synthesized in the human body. They should therefore be provided by the diet, and their supplementation is recommended in special conditions of deficiency.^88^

Trans fatty acids also have a long chain and are mainly represented by elaidic acid (18:1, n-9t), found in vegetable fats resulting from the partial hydrogenation of vegetable oils during preparation.^109 , 110^ Trans fatty acids are found in minute amounts in meat and milk in the form of vaccenic acid (18:1, n-11t), which is produced through the biohydrogenation of fats under the action of the rumen microbiota of ruminants.^109^

### 10.2. Fat Absorption

Dietary fats include triglycerides (90% to 95%), phospholipids, cholesterol, and fat-soluble vitamins. Although the intestine is the major site for digestion of fats, this process begins minimally in the oral cavity, through exposure to lingual lipases, followed by the stomach, where 10% to 30% of fatty acids are released, starting the process of fat emulsification.^89 , 111^ Digestion continues in the intestine, where hydrolysis of the remaining triglycerides is induced by pancreatic lipase activity, releasing fatty acids and monoacylglycerol.^112 , 113^

The mechanism of fatty acid absorption is complex because of multiple absorption systems.^114^ Short-chain fatty acids (acetate, propionate, and butyrate) are absorbed primarily via sodium-dependent or non-sodium-dependent active transport mediated by monocarboxylate transporters. However, G protein-coupled receptors (GPCRs) may also participate in the absorption of these fatty acids, such as GPR41 and GPR43. Medium-chain fatty acids (capric, caprylic, and caproic acids) are absorbed mainly via passive transport, but GPR84 may also play a role in their incorporation into the enterocyte surface.^115^ After absorption, they bind to albumin and travel via the portal circulation to the liver.^114^ Conversely, more complex mechanisms are required to absorb long-chain fatty acids (saturated, unsaturated, or trans), and their transport in the plasma depends on the formation of chylomicrons.^96 , 97^ They can be absorbed by passive diffusion, when the luminal concentration is higher than the intracellular concentration, or through membrane receptors/transporters. For example, the transporter cluster of differentiation 36 (CD 36) allows the uptake of long-chain fatty acids even when their luminal concentrations are lower than those inside the cell.^116^ The fatty acid transporter protein 4 (FATP4) is widely distributed in the intestine and one of the main long-chain fatty acid transporters.^117^ Within enterocytes, fatty acids are transported by proteins such as fatty acid-binding protein 1 (FABP1) and FABP2^116^ and re-esterified, returning to the form of triglyceride by the action of the diacylglycerol acyltransferase (DGAT) enzyme.^89^ Triglycerides are then incorporated into ApoB48 via the microsomal triglyceride transfer protein, which initiates the formation of chylomicrons.^118^ Chylomicrons are processed in the Golgi complex and subsequently secreted into the lymph, entering the blood circulation via the thoracic duct.^89 , 90^

In the bloodstream, chylomicron triglycerides are hydrolyzed by LPL, which is adhered to the endothelium of extrahepatic tissues, releasing free fatty acids that subsequently bind to albumin, being stored mostly in adipose tissue and only minimally in muscle tissue.^119^ Chylomicron hydrolysis generates chylomicron remnants, which are removed from the circulation through their interaction with hepatic B/E receptors and LDL receptor-related protein.^120^

### 10.3. Nutritional Treatment

#### 10.3.1. Fats

Triglyceride lipolysis is defective in FCS because of mutations in the *LPL* gene or its cofactors ( *GPIHBP1* , *LMF1* , *APOA5* , or *APOC2* ), so long-chain fatty acids should be minimally consumed to prevent elevated plasma chylomicron concentrations.^27^ Restricting dietary fat intake to 10% of daily TEI is therefore recommended.^92^ However, depending on the severity of the disease, total fat intake can be further restricted to less than 5% daily calories.^91^

In addition to strict adherence to a very-low-fat diet, SFA-rich foods should be consumed in small amounts. SFAs are involved in important hepatic lipogenic pathways by activating the sterol regulatory element binding protein-1c (SREBP-1c), which acts as a transcription factor coding for the genes of acetyl-CoA carboxylase, fatty acid synthase, and stearoyl-CoA desaturase-1,^121 , 122^ enzymes involved in fatty acid synthesis, precursors of the synthesis of triglycerides, by the action of DGAT.^123^

Although ω3 unsaturated fatty acids regulate triglyceride synthesis by blocking SREBP-1c, they are not recommended for the treatment of FCS even at high doses, because individuals do not have a defect in the hepatic synthesis of triglycerides, but rather in triglyceride hydrolysis.^27^ However, because they are considered essential fatty acids, both ALA (ω3) and linoleic acid (ω6) supplementation may be necessary for patients with FCS to prevent deficiency. The Global Burden of Disease Study *,* ^124^ a study conducted in 197 countries, suggests an optimal intake of ω6 of 11% of daily TEI, although the global average consumption is 4.5% of TEI. Regarding ω3, the optimal intake is 0.25 g/d, with a global average consumption of 0.1 g/d.^124^ The Recommended Dietary Allowances (RDA) recommend a daily ω3 intake of 0.5 to 1.4 g, depending on the age group.^125^

#### 10.3.2. Medium-chain Triglycerides

MCTs contain caproic (C6:0), caprylic (C8:0), or capric (C10:0) SFAs, which are obtained by fractionating coconut or palm oils and are commercially available, together or separately. They may have a small amount of lauric acid (maximum of 1% to 2%).^126 , 127^ Individuals with FCS are allowed to consume MCTs because these fatty acids are absorbed almost entirely via the portal circulation, being minimally incorporated into chylomicrons.^128 , 129^ It is important to note that lauric acid (C12:0) is considered a long-chain fatty acid that is transported mainly via chylomicrons, being transported via the portal circulation only when its storage capacity in this lipoprotein is exceeded.^129 , 130^ Therefore, it is essential to carefully observe the fatty acid composition of the product, which should preferably contain no or minimal concentrations of lauric acid to prevent an increase in chylomicron concentrations.

MCTs are indicated to contribute to energy intake in infants, children, and adults with FCS, as an adjunct to treatment. However, tolerability must be tested because people have reported gastrointestinal discomfort after use of MCTs.^92^

#### 10.3.3. Carbohydrates

Food sources of complex carbohydrates, rich in fiber, should be consumed, such as brown rice, beans, peas, lentils, and chickpeas. Fruit intake is recommended in adequate amounts, with a maximum of 3 to 4 servings per day, so as not to exceed the recommended daily sugar intake. Some authors suggest limiting total carbohydrate intake to 60% of daily TEI.^92^ Added sugars (sucrose and corn syrup) should be avoided because they induce an increase in the hepatic synthesis of fatty acids, contributing to elevated plasma triglyceride concentrations. Sugars contain glucose and fructose, and the latter promotes intense hepatic lipogenesis not only by serving as a substrate for fatty acid synthesis but also by stimulating the expression of enzymes involved in *de novo* lipogenesis via activation of carbohydrate-responsive element binding protein and SREBP-1c.^131 - 133^ In addition, excessive fructose intake decreases fatty acid beta-oxidation by inducing post-translational modifications in mitochondrial proteins, reducing the number and size of these organelles.^134^

Therefore, patients with FCS should avoid the consumption of concentrated fruit juices because of intense fructose-induced lipogenic activity.

#### 10.3.4. Alcohol

Patients should abstain from consuming alcohol because it can elevate plasma triglyceride concentrations. Alcohol metabolization begins minimally in the stomach by the action of alcohol dehydrogenase (ADH), but it is metabolized mainly by the liver through three pathways: cytochrome P450 2E1, catalase, and ADH. Alcohol metabolization leads to form acetaldehyde, which is converted into acetate by aldehyde dehydrogenase, with the main participation of ADH.^135^ Acetate can be converted to fatty acids, precursors of triglyceride synthesis.^136^

#### 10.3.5. Infants and Early Childhood

Providing infants with adequate quantity and quality of nutrients during the first 2 years of life is essential to promote adequate growth and cognitive development, in addition to consolidating healthy eating habits. However, developing an eating plan for infants and children with FCS that ensures proper dietary intake of recommended amounts of macronutrients and micronutrients is a challenging task for the nutritionist and the family because of the severe dietary fat restriction. Nutritionist training in this area is of paramount importance to balance the diet, to develop sample menus for the family, and to closely monitor the implementation of new eating habits. The family should be aware that dietary fat intake above the recommended amount, even in minimal amounts, can cause an undesirable increase in plasma chylomicron concentrations.

For breastfed infants with FCS, breastfeeding must be discontinued as soon as the diagnosis is confirmed, which can cause frustration and sadness for both the mother and child. Breast milk has approximately 3.2% fat, with triglycerides accounting for approximately 98% of the lipid fraction. The exact fatty acid composition depends on the mother’s diet and varies significantly during the breastfeeding period.^137^ Milk triglycerides consist mainly of long-chain SFAs (35% to 40%), MUFAs (45% to 50%), and PUFAs (15%), with a predominance of palmitic, oleic, and linoleic acids, respectively.^137 , 138^ Unsaturated fatty acids with a chain length of more than 20 carbons, containing two or more double bonds, represent only 2% of the total fatty acids present in breast milk.^138^ Fatty acids of the ω3 series are found in small amounts in breast milk: ALA (0.019 g/100 mL), EPA (0.003 g/100 mL), and DHA (0.008 g/100 mL).^139 , 140^

An important study conducted in Europe (European Childhood Obesity Project) followed up 174 children from birth to age 1 year and contributed to a better understanding of the caloric intake of lipids, carbohydrates, and proteins through the first year of life, with results that can be extrapolated to provide dietary guidance for infants who cannot be breastfed.^139^ Average daily energy intake was 419 kcal at 1 month, 589 kcal at 6 months, and 860 kcal at 12 months. Fat intake was 21 g/day within the first 6 months, gradually increasing to 34.2 g at the end of 12 months. Regarding essential fatty acids, considering exclusive breastfeeding up to 3 months of age, the average daily intake of ALA (ω3) was 0.118 g and of linoleic acid (ω6) was 2.40 g. Regarding marine fatty acids, the average daily intake of EPA was 0.022 g and of DHA was 0.048 g.^139^ According to the Academy of Nutrition and Dietetics, the recommended intake of ω3 fatty acids is 0.5 g/day for infants aged 0 to 12 months and 0.7 g/day for children aged 1 to 3 years, whereas the recommended intake of ω6 fatty acids is 4.6 g/day for infants aged 0 to 6 months and 7 g/day for children aged 7 to 12 years.^125^

Given the severe dietary fat restriction, monitoring dietary intake of fat-soluble vitamins (A, E, D, and K) is recommended,^92^ and the RDA for infants and children are available in the table of the Dietary Reference Intakes.^95^

Special infant formulas that partially resemble the nutritional composition of breast milk are recommended as a substitute for breast milk.^92^ Regarding fat content, formulas should be prepared only with medium-chain fatty acids (capric, caprylic, and caproic acids), in addition to providing fat-soluble vitamins and allowed amounts of essential fatty acids. In addition, MCTs can be recommended to achieve optimal energy intake, according to tolerance, because they are minimally transported by chylomicrons.^128 , 129^

The introduction of solid foods such as vegetables, fruits, and lean meats (e.g., fish, skinless chicken breast, lean beef), and grains should follow the recommendations of national and international pediatric societies, limiting dietary fat intake to 10% of daily TEI.

Adequate fluid intake is recommended to maintain fluid balance, which will contribute to pancreatic function. Prolonged dehydration induced by vomiting and diarrhea is known to increase the risk of pancreatitis associated with FCS.^92^

Dietary guidelines should be individualized and enjoyable, respect cultural habits, and be sustainable in the long term. Children should be informed of the importance of reading food labels, and the family should be instructed to prepare meals containing minimal amounts of fat, in addition to highlighting the importance of preparing meals/foods at home.

#### 10.3.6. Pregnant Women

During pregnancy, a rise in plasma lipid concentrations is expected especially at the end of the second and third trimesters, with two- to four-fold increased triglyceride concentrations, which are well tolerated by the patient. In this phase, increased insulin resistance and the action of placental hormones contribute to greater adipose tissue lipolysis. In addition, there is increased hepatic output of VLDL and decreased hepatic lipase activity. LPL activity is also reduced, which impairs the hydrolysis of lipoprotein triglycerides. Because of these alterations, hepatic clearance of triglyceride-rich lipoproteins is consecutively reduced, leading to elevated plasma triglyceride concentrations.^141 , 142^

Increased triglyceride levels during pregnancy are associated with an increased risk of complications for the mother and child by increasing the risk of AP, which may lead to miscarriage, early delivery, and even death. During pregnancy, although rare, AP is often caused by biliary lithiasis. Elevated cholesterol concentrations and gallbladder hypomotility caused by the hormonal profile characteristic of pregnancy predisposes women to calculus formation, which may obstruct the pancreatic duct. Women with FCS show a marked increase in triglyceride concentrations, which may trigger AP. Pregnant women with FCS are at a 4% increased risk of developing AP with triglyceride levels > 1,000 mg/dL, and at a 14% increased risk with levels > 2,600 mg/dL.^94^

The dietary treatment of pregnant women with FCS aims to maintain plasma triglyceride levels less than 500 mg/dL throughout pregnancy. To this end, a fat-restricted diet (less than 20 g/day) is required, along with adequate intake of vitamins, minerals, and essential fatty acids, according to the recommended intake for the stage of pregnancy, including monitoring of maternal weight gain. Patients following a very-low-fat diet should be monitored regularly to ensure proper dietary intake of calories, macronutrients, and micronutrients, especially essential fatty acids^143^ and fat-soluble vitamins.^92^

MCTs (without long-chain fatty acids) may be indicated to achieve optimal energy intake if necessary. In addition, adequate fluid intake is recommended to maintain adequate fluid and electrolyte balance. Pregnant women with FCS associated with type 2 diabetes mellitus or gestational diabetes need greater attention for proper adherence to the diet, requiring multidisciplinary follow-up to manage lipid levels, glycemia, and fetal development.^92^

Dietary management of FCS is the only tool available to control plasma triglyceride levels in this condition, as demonstrated in a recent case report of a pregnant woman with plasma triglyceride levels of 8,683 mg/dL who experienced previous episodes of pancreatitis.^144^

The nutritionist should help patients develop their own eating plan, providing assistance with recipes and strategies that facilitate adherence to the diet. Dietary preferences, cultural habits and lifestyle should be considered, as well as nutritional adequacy of the diet and energy intake. An extreme very-low-fat diet is difficult to maintain. Therefore, monitoring by a multidisciplinary team is extremely important to manage lipid levels and minimize the risk of complications.^92^

#### 10.3.7. General Recommendations

1. Restricting dietary fat intake (10% to 15% of TEI);
2. Avoiding added sugars (sucrose and corn syrup);
3. Avoiding concentrated fruit juices;
4. Abstaining from consuming alcohol;
5. Consuming complex carbohydrates in adequate amounts;
6. Ensuring adequate intake of essential fatty acids;
7. Monitoring the intake of fat-soluble vitamins, with supplementation if necessary;
8. Introducing MCTs to achieve adequate energy intake, according to tolerance.

## 10.4. Sample Menus

**Table:** 

Menu 1,500 kcal	Servings	Lipids	kcal
**Breakfast**			
Skim milk	200 mL	0	61
Whole wheat bread	2 slices	1.6	120
Cottage cheese	1 tablespoon	0.8	28
Papaya	½ unit	0	46
Oat	1 tablespoon	1.7	40
**Morning snack**			
Fig	3 units	0	80
**Lunch**			
Green salad, tomato, heart of palm, cucumber	1 dinner plate	0	10
Dressing	1 tablespoon	0	5
Rice, cooked without oil	3 tablespoons	0.2	100
Black beans (50% broth) without oil	1 ladle	0.4	65.3
Shark meat with tomato sauce and basil	200 g	1.6	232
Sautéed broccoli with garlic and onion	2 tablespoons	0	30
Pineapple	1 slice	0.1	70
**Afternoon snack**			
Nonfat yogurt	200 mL	0.6	88
Whole wheat bread	1 slice	1.9	60
Sugar-free jam	½ tablespoon		30
Light ricotta cream	1.5 tablespoon	2.1	44
**Dinner**			
Green salad, tomato, heart of palm, cucumber	1 dinner plate	0	10
Dressing	1 tablespoon	0	5
Egg white omelet filled with spinach, tomato, and zucchini	2 egg whites	0	80
Baked potato with garlic, coarse salt, and rosemary	1 medium unit	0	100
Baked banana with cinnamon	1 unit	0	70
**Supper**			
Nonfat yogurt	200 mL	0.6	88
**Total in grams**		11.6	1462.3
**% kcal**		7.1	

**Table:** 

Menu 1,800 kcal	Servings	Lipids	kcal
**Breakfast**			
Skim milk	200 mL	0	61
Whole wheat bread	2 slices	1.6	120
Light Minas cheese	1 slice	2.3	50
Banana	1 unit	0	70
Rolled oats	1 tablespoon	1.7	40
**Morning snack**			
Persimmon	1 unit	0.2	70
Tapioca	3 tablespoons	0	100
Light ricotta cream	1.5 tablespoon	2.1	44
**Lunch**			
Green salad, tomato, heart of palm, cucumber	1 dinner plate	0	10
Dressing		0	5
Rice with vegetables	4 tablespoons	0.3	128
Lentils (50% broth)	1 ladle	0	93
Mediterranean acoupa weakfish	120 g	1.3	91
Roasted vegetables with rosemary	2 tablespoons	0	80
Guava	1 unit	0.5	60
**Afternoon snack**			
Skim milk blended with papaya and apple	200 mL	0	150
Whole wheat bread	1 slice	1.9	60
Oven-baked ricotta with tomato and oregano	30 g	2.4	53
**Dinner**			
Green salad, tomato, heart of palm, cucumber	1 dinner plate	0	10
Dressing	1 tablespoon	0	5
Pasta with tomato sauce, cooked without oil	150 g	0.7	185
Grilled chicken with rosemary	80 g	2	127
Fruit salad	150 mL	0	120
**Supper**			
Nonfat yogurt	200 mL	0.6	88
**Total in grams**		17.6	1820
**% kcal**		8.7	

**Table:** 

Menu for pregnant women 1,800 kcal	Servings	Lipids	kcal
**Breakfast**			
1 glass of skim milk	250 mL	0	75
Light whole wheat bread	2 slices	0.5	96
Light Minas cheese	1 slice	2.3	50
Banana	1 unit	0	70
Oat bran	1 tablespoon	1.1	49
**Morning snack**			
Nonfat yogurt	200 mL	0	88
Strawberry	10 units	0.3	30
**Lunch**			
Green salad, tomato, heart of palm, cucumber	1 dinner plate	0	10
Dressing	2 tablespoons	0	5
Brown rice, cooked without oil	4 tablespoons	0.6	120
Black beans (50% broth) without oil	1 ladle	0	50
Acoupa weakfish with passion fruit sauce	150 g	1.7	114
Roasted vegetables with rosemary	2 tablespoons	1	120
Melon	2 slices	0	60
**Afternoon snack**			
Skim milk blended with	250 mL	0	75
berries	50 g		28
Ricotta	30 g	2.4	53
Tapioca	2 tablespoons	0	74
Sugar-free jam	½ tablespoon	0	30
**Dinner**			
Green salad, tomato, heart of palm, cucumber	1 dinner plate	0	10
Dressing	2 tablespoons	0	5
Pasta with tomato sauce, cooked without oil	150 g	0.7	185
Oven-baked tuna with herbs	150 g	1.1	180
Papaya	½ unit	0	46
**Supper**			
Nonfat yogurt	200 mL	0.6	88
Rolled oats	1 tablespoon	1.7	40
**Total in grams**		14	1,751
**% kcal**		7.2	

**Table:** 

Menu for children 1,400 kcal	Servings	Lipids	kcal
**Breakfast**			
Oat porridge with banana			
1 glass of skim milk + vitamins A+D+E	200 mL	0	67
Banana	1 unit	0	70
Oat bran	1 tablespoon	1.1	49
Whole wheat bread	1 slice	0.8	60
Light ricotta cream	15 g	1	22
Morning snack			
Nonfat yogurt blended with	200 mL	0	99
papaya and	½ unit	0	46
Fortified skim milk powder (with vitamins A+D+E)	1 tablespoon	0	50
**Lunch**			
Green salad, tomato, heart of palm, corn, and beans	1 dessert plate	0	28
Dressing	1 tablespoon	0	5
Brown rice	3 tablespoons	0.6	75
Black beans (50% broth)	3 tablespoons	0	50
Shredded chicken breast with tomato sauce	4 tablespoons	2	127
Sautéed zucchini	3 tablespoons	0	30
Melon	1 slice	0	28
**Afternoon snack**			
Tapioca with	30	0	108
light Minas cheese	30	2.3	50
Tomato and oregano	0	0	0
Sugar-free passion fruit juice	0	0	0
**Dinner**			
Green salad, tomato, heart of palm, cucumber		0	0
Dressing		0	5
Rice with vegetables	2 tablespoons	0.15	75
Lentil	3 tablespoons		45
Turkey breast with rosemary	70	2.5	130
Dates	2 units	0	70
**Supper**			
Warm fortified skim milk (with vitamins A+D+E) with cloves and cinnamon	200 mL	0	67
Cocoa powder	1 tablespoon	1.4	26
**Total in grams**		11.85	1382
**% kcal**		7.7	

**LOW-FAT FOODS (< 5 g per serving)**

**Table:** 

	**IN 100 g**			**Per serving**		
**amount of fat**	**fat**	**ω6 g**	**ω3 g**	**retinol (ug)**	**g food**	**g of fat**
**FISH**						
raw pollock fillet	0.4	tr	nd	tr	120	0.5
raw sevengill shark in steak	0.67	tr	nd	6	120	0.8
raw tuna	0.9	0.01	0.01	20	120	1.1
raw acoupa weakfish	1.1	0.01	0.01	tr	120	1.3
raw spotted sorubim, skin removed	1.3	0.02	0.02	tr	120	1.6
raw corvina	1.6	0.01	0.02	65	120	1.9
raw hake	2	0.03	0.05	tr	120	2.4
fish sole fillet	3	nd	nd	nd	120	3.6
raw hake fillet	4	0.03	0.04	48	120	4.8
	**IN 100 g**			**Per serving**		
**MEATS**	**fat**	**ω6 g**	**ω3 g**	**retinol (ug)**	**g food**	**g of fat**
grilled beef top rump	2.4	0.19	0.04	nd	80	1.9
grilled chicken breast, skin removed	2.5	0.31	0.01	tr	80	2.0
raw beef eye of round	5.2	0.06	0.01	3	80	4.2
grilled beef bottom sirloin without fat	7.3	0.17	0.03	tr	80	5.8
cooked beef eye of round	9.1	0.12	0.02	2	80	7.3
cooked beef foreshank without fat	7.4	0.16	0.01	tr	80	5.9
cooked chicken drumstick, skin removed	5.8	1.1	0.05	tr	80	4.6
cooked beef shin without fat	6.7	0.09	0.03	2	80	5.4
	**IN 100 g**			**Per serving**		
**DAIRY**	**fat**	**ω6 g**	**ω3 g**	**retinol (ug)**	**g food**	**g of fat**
nonfat yogurt	0.3	tr	nd	nd	200	0.6
skim milk	0	nd	nd	tr	200	0.0
light cottage cheese	0	nd	nd	nd	30	0.0
cottage cheese	2.6	nd	nd	nd	30	0.8
light ricotta cream	7.1	nd	nd	nd	30	2.1
ricotta	8.1	0.14	0.02	53	30	2.4
light cream cheese	13	nd	nd	nd	20	2.6
light Minas cheese	7.7	nd	nd	nd	30	2.3
light Minas cream cheese	14,7	nd	nd	nd	15	2.2
light mozzarella cheese	17	nd	nd	nd	30	5.1
mozzarella cheese	25,2	0.31	0.08	109	30	7.6
Minas cheese	20,2	0.28	0.06	161	30	6.1
	**IN 100 g**			**Per serving**		
**EGGS**	**fat**	**ω6 g**	**ω3 g**	**retinol (ug)**	**g food**	**g of fat**
boiled egg white	0.1	0	0	0	3.2	0.0
whole chicken egg	9.5	0.94	0.02	32	50	4.8
	**IN 100 g**			**Per serving**		
**BREAD AND PASTA**	**fat**	**ω6 g**	**ω3 g**	**retinol (ug)**	**g food**	**g of fat**
tapioca	0	0	0	0	45	0.0
cooked spaghetti	0.9	nd	nd	nd	100	0.9
raw wheat pasta	1.3	nd	nd	nd	50	0.7
fiber-rich whole grain bread	3.31	nd	nd	nd	25	0.8
light whole wheat bread	1	nd	nd	nd	50	0.5
	**IN 100 g**			**Per serving**		
**CEREALS**	**fat**	**ω6 g**	**ω3 g**	**retinol (ug)**	**g food**	**g of fat**
quinoa flakes						0.0
rice, cooked without oil	0.41	0.31	0.01	nd	60	0.2
brown rice, cooked without oil	0.2	0.06	nd	nd	60	0.1
popcorn kernel	4	nd	nd	nd	16	0.6
oat bran	7	nd	nd	nd	20	1.4
raw rolled oats	8.5	2.95	0.06	nd	20	1.7
flaxseeds	32.3	5.42	19.81	nd	10	3.2
chia seeds	34.4	17.36	62.02	nd	10	3.4
	**IN 100 g**			**Per serving**		
**LEGUMES**	**fat**	**ω6 g**	**ω3 g**	**retinol (ug)**	**g food**	**g of fat**
cooked chickpeas	2.6	2.71	0.13	nd	65	1.7
cooked lentils	0.5	0.21	0.04	nd	50	0.3
cooked black beans with broth	0.5	0.16	0.14	nd	86	0.4
sugar-free cocoa powder	13.5	nd	nd	nd	10	1.4
**fruits to avoid**	**fat per serving**					
pequi (souari nut)	18				60	10.8
avocado	8.4				100	8.4
coconut	4				30	12.6
acai	3.9				250	9.8

*na: not available; tr: traces; lipids (g) per serving. *

## 11. Apheresis

Triglycerides > 1,000 mg/dL increase the risk of pancreatitis in patients with FCS. Class IIA, Level C.

AP is the most frequent complication in patients with FCS, with a prevalence of 60% to 88%.^145^ Plasma triglycerides > 1,000 mg/dL may be indicative or greatly increase the risk of hypertriglyceridemic pancreatitis (HP).

AP mortality in patients with FCS is approximately 6% but may reach up to 30%, depending on the presence of complications.^146 , 147^ Cohort studies have demonstrated a more severe evolution in these patients, with a higher prevalence of complications (shock, renal and respiratory failure, sepsis) compared with other etiologies of AP.^148 , 149^

### 11.1. Diagnosis and Treatment

The initial diagnostic and therapeutic procedures for HP should follow the same practices recommended for AP cases in general (including intravenous fluid therapy, analgesic treatment, and fasting). The earliest possible determination of serum triglyceride levels is crucial, as they may decrease in the first 48 hours after the onset of pancreatitis as a result of fasting.^150^

In patients with FCS, HP may occur spontaneously, with no apparent cause, or be triggered by secondary factors including uncontrolled diabetes, alcohol abuse, pregnancy, and medications (oral estrogens, tamoxifen, propofol, valproic acid, isotretinoin, clomiphene, beta blockers, protease inhibitors, and mirtazapine).^68^

### 11.2. Nondrug Therapy

The mainstay of initial AP therapy is admission to the intensive care unit, as well as oral intake restriction, intravenous fluids, and analgesia. Clinical evolution depends on the reduction of plasma triglycerides within the first 24 to 48 hours of admission. Most patients with HP have an uncomplicated clinical course, with good prognosis. In general, serum triglyceride levels decrease within 24 to 48 hours of admission and reach values < 500 mg/dL on the fourth or fifth day only with support measures.^151^ Once the pain subsides and gastrointestinal transit is established, an oral fat-free diet may be reinstated.

### 11.3. Pharmacological Treatment

**Intravenous heparin infusion for HP is not recommended in patients with FCS. Class of recommendation: III, Level of evidence: C.**

Heparin and insulin infusions have been used as the main therapy for HP, with most evidence coming from single cases or case series.^152 - 156^ The infusion of unfractionated heparin can release LPL bound to endothelial cells, leading to a temporary reduction in serum triglycerides. In severe cases of HP, long-term intravenous heparin infusion can deplete LPL from the surface of endothelial cells, allowing serum triglyceride levels to rise again.^157 - 159^ In addition, some authors are reluctant to recommend the use of intravenous heparin in patients with pancreatic necrosis due to the risk of hemorrhagic transformation.^151^

The use of low-molecular-weight heparin is indicated as prophylaxis for deep venous thrombosis in HP in patients with FCS. Class II A, level C.

There are no contraindications for the use of low-molecular-weight heparin^160^ as prophylaxis for deep vein thrombosis in HP.

Intravenous insulin should only be used for glycemic control in HP in patients with FCS and decompensated type 1 or 2 diabetes. Class of recommendation: IIa, Level of evidence: C.

Insulin increases LPL activity and helps to reverse the effects of insulin resistance on the liver. Insulin infusion is especially useful in patients with uncontrolled diabetes and hyperglycemia in addition to HTG. There is no clear evidence of the benefit of insulin in patients with HP who are not diabetic.^19^

Intravenous insulin therapy must be initiated in patients with severe HTG and HP who also have uncompensated type 1 diabetes.^160 - 162^ Intravenous insulin should be initiated in patients with severe HTG and HP who also have decompensated type 2 diabetes.^163 - 165^

### 11.4. Apheresis

Plasmapheresis should be indicated in patients with FCS and HP on an individual basis. Potential candidates are patients with severe HP or persistent triglycerides > 1,000 mg/dL after the first 24 to 48 hours. Class of recommendation: IIb, Level of evidence: C.

Case reports and series have demonstrated the efficacy of plasmapheresis in removing triglycerides from the circulation of patients with HP, with a mean reduction in triglyceride levels between 65% and 85% after 1 or 2 sessions.^166 - 170^

Because HP is a life-threatening condition, some centers use plasmapheresis as the procedure of choice to rapidly reduce circulating chylomicrons as soon as the diagnosis is established, thus removing the agent responsible for pancreatic damage. The early use of this procedure to reduce plasma triglycerides would prevent the production and accumulation of free fatty acids, reducing their local and systemic effects.^171 - 172^ The mechanism of HTG-induced AP is probably caused by excess triglycerides, which leak from acinar cells into the vascular bed of the pancreas when hydrolyzed by pancreatic lipase, resulting in accumulation of free fatty acids and lysolecithin. Free fatty acids are toxic and can cause damage to acinar cells and the capillary endothelium.^173^ In addition, elevated concentrations of chylomicrons increase the blood viscosity of veins with impaired local blood flow, resulting in pancreatic ischemia and worsening of tissue damage.^174^ Free fatty acids activate trypsinogen, which leads to local edema and necrotizing pancreatitis.^173^ A case series published in a tertiary hospital in Turkey included 33 patients with HTG-related AP and showed a mean triglyceride reduction of 54.4% after a single session of plasmapheresis. After a second session, there was a 79.4% reduction in triglycerides. During clinical course, 13 patients had pancreatic fluid collection, with 1 case of necrotizing pancreatitis and no cases of pseudocyst. Mortality in patients with severe HP was 33.3%, and overall mortality was 3%, with no cases related to plasmapheresis. The study demonstrated that plasmapheresis is a safe and effective treatment for patients with HP. More studies are needed to compare apheresis + conservative treatment with only conservative treatment in patients with HP.^175^

Chen et al.^167^ retrospectively analyzed clinical outcomes in patients with HP before (n = 34) and after (n = 60) the availability of apheresis at their institution. The groups had similar clinical features. In 20 patients from the second group, plasmapheresis was initiated with a mean time of 3 days after symptom onset. There were no significant differences in terms of mortality and complications between patients undergoing or not undergoing plasmapheresis. Study limitations include the retrospective design, single-center experience, and small sample size.^167^

Some centers perform plasmapheresis on admission, shortly before 24 hours, whereas others perform the procedure within 24 to 72 hours of admission. Studies have emphasized the importance of early initiation of plasmapheresis in HP, whereas others have not detected any difference in morbidity and mortality with early or late initiation of the procedure.^167^ A clear benefit of plasmapheresis in reducing HP severity has yet to be conclusively demonstrated.

Plasmapheresis is not risk-free and is a costly procedure. It requires central intravenous access and temporary anticoagulation, with associated complications that include bacteremia, venous thrombosis, and bleeding. Potential candidates are those with severe HP or persistent triglyceride levels > 1,000 mg/dL after the first 24 to 48 hours of admission.^175^

Due to the lack of evidence, recommendations for plasmapheresis in adults with HP and FCS should be individualized. In recent American Society for Apheresis (ASFA) guidelines, the recommendation for plasmapheresis in patients with HP is 2C (weak recommendation), with a level of evidence of III.^176^

### 11.5. Pregnancy and HP in Patients with FCS

The indication of plasmapheresis during pregnancy, although safe and effective, should be individualized due to the scarcity of evidence to date. Class of recommendation: IIb, Level of evidence: C.

Normal pregnancy is characterized by adaptive changes in lipid metabolism to meet the needs of the placenta and the glucose and lipid requirements for fetal growth, including increased glucose production, progesterone synthesis, lipogenesis, and reduced lipolysis.^177 , 178^ Patients with genetically determined alterations in lipid metabolism, characterized by reduced intravascular lipolysis, may evolve during pregnancy with severe HTG and pancreatitis.^179^

HP is developed in the third trimester of pregnancy or at the beginning of the postpartum period, with a major impact on maternal and fetal morbidity and mortality.^180^ Rates of maternal and fetal mortality due to HP of 37% and 60%, respectively, have already been described, but these numbers are currently declining due to diagnostic and therapeutic advances.^181 - 183^

Pregnancy-associated pancreatitis may occur in the setting of gallstone disease, alcohol abuse, and HTG.^146^ In cases of HP, the severity score and the worst prognosis are more prevalent than other etiologies of AP.^64 , 184^ Clinical case reports have shown that the use of plasmapheresis in pregnant women is effective and safe.^185 - 188^ However, due to the scarcity of evidence, the indication of plasmapheresis in pregnancy complicated by HP, in patients with FCS, should be individualized.

## 12. New Therapies for the Treatment of Familial Chylomicronemia Syndrome

Treatments available for patients with FCS aimed at reducing triglyceride levels are not effective in controlling chylomicronemia.^24^ Gene therapy with AAV1-LPL(S447X) using an adeno-associated virus was tested in the setting of FCS (Glybera, alipogene tiparvovec) with the aim of expressing LPL(S447X). However, despite promising results, the commercial use of AAV1-LPL (S447X) was not possible due to its high cost.^189^ Thus, the only therapy that reduces triglycerides to < 880 mg/dL, or 10 mmol/L, in patients with FCS and which seems to reduce the risk of pancreatitis is a fat-restricted diet associated with alcohol restriction and certain medications.^92^ Lifelong adherence to these restrictions is difficult, and episodes of chylomicronemia, abdominal pain, and recurrent pancreatitis are common. Thus, additional therapies are needed to maintain triglycerides levels < 880 mg/dL.

### 12.1. APOC3

ApoC3 is a glycoprotein consisting of 79 amino acids, synthesized primarily in the liver and to a lesser extent in the intestine, and is associated with ApoB-containing lipoproteins, including chylomicrons, VLDLs, and HDLs.^190 - 192^ In genetic, preclinical, and phase 1 studies, ApoC3 has emerged as a regulator of plasma triglyceride concentrations.^192^ ApoC3 is an inhibitor of LPL activity^190^ and a potent inhibitor of LPL activation that is mediated by ApoC2, resulting in the inhibition of lipolysis of triglyceride-rich lipoproteins.^190^ ApoC3 has also been shown to inhibit hepatic lipase activity, to promote VLDL assembly and secretion,^193^ and to inhibit clearance of triglyceride-rich lipoproteins remnants.^194^ However, the importance of these LPL-independent mechanisms is not well understood.

#### 12.1.1 Antisense Inhibition of ApoC3

Volanesorsen is a second-generation antisense drug that inhibits the synthesis of modified apoC3. ISIS 304801 has a 2’-O-(2-methoxyethyl) end.^192^ Inhibition of ApoC3 synthesis in the liver occurs through sequence-specific binding of ISIS 304801 to *APOC3* mRNA, which in turn leads to the degradation of *APOC3* mRNA by RNase H1, an endogenous ribonuclease expressed in mammalian cells.^191^ In phase 1 studies with healthy volunteers, ISIS 304801 promoted a dose-dependent and prolonged reduction of ApoC3 plasma concentrations with concomitant triglyceride lowering.^192^ In phase 2 studies, ISIS 304801 was effective in lowering triglycerides in patients with elevated VLDL due to different conditions.^195^

Because patients with FCS have very low LPL activity and because lipolysis inhibition by the LPL-dependent pathway is a mechanism of action of ApoC3, ISIS 304801 would be predicted to be ineffective or to have a minimal effect in lowering triglycerides in patients with this syndrome. However, there must be an LPL-independent escape mechanism for the survival of these patients. Preclinical studies suggest that ApoC3 modulates triglyceride levels through an LPL-independent pathway. A study was conducted with ISIS 304801 in patients with FCS and triglycerides levels from 1,406 to 2,083 mg/dL. After 13 weeks of treatment with 300 mg of volanesorsen, plasma concentrations of ApoC3 and triglycerides were reduced in 71% to 90% and from 56% to 86%, respectively. During treatment, all patients had triglycerides < 500 mg/dL. Initial data showed the role of ApoC3 as a regulator of triglyceride metabolism through LPL-independent pathways.^191^

These outcomes were replicated in the Approach clinical trial,^57^ a 52-week, randomized, double-blind, phase 3 study that evaluated the efficacy and safety of volanesorsen in 66 patients with FCS. Patients were randomly assigned in a 1:1 ratio to receive volanesorsen or placebo. The primary endpoint was the percentage change in fasting triglycerides from baseline to 3 months (at week 12 or week 13). Nine secondary endpoints were prioritized and analyzed in hierarchical order. If analysis of the first endpoint was significant, the second endpoint in the hierarchy would be analyzed for significance, and so on. If an endpoint was nonsignificant in the hierarchy, analysis of all subsequent endpoints would be exploratory. Percentage changes from baseline to 6 months and to 12 months were compared between treatments using analysis of covariance (ANCOVA).

A total of 130 patients were selected, of whom 67 underwent randomization; 1 patient from the placebo group withdrew consent. Of the 66 patients, 41 were homozygous or compound heterozygous for at least 1 of 25 inactivating mutations in *LPL* , and 11 patients had biallelic mutations in accessory proteins or were double heterozygous for *LPL* and *APOA5* or *LMF1* mutations. Fourteen patients had no defined mutations but were included on the basis of their clinical phenotype and low LPL activity.^23^

Included patients were aged 20 to 75 years, 80% were white, and 55% were women; the mean body mass index was 25.0 ± 5.7. Age at FCS diagnosis ranged from 1 to 75 years. *Lipemia retinalis* occurred in 21% and eruptive xanthomas in 23% of patients; 76% had a documented history of AP, of whom 23 had had 53 adjudicated AP episodes in the previous 5 years. Seven patients had chronic pancreatitis.

At baseline, 53% of patients were taking fibrates, ω3 fatty acids, or both, and 20% were receiving statins. Seven patients had been treated with alipogene tiparvovec (Glybera) more than 2 years before they were included in the study. Baseline triglyceride levels were elevated and did not differ between patients who were receiving medication and those who were receiving placebo (2,209 ± 1,199 mg/dL), likewise VLDL chylomicrons (1,849 ± 1,176 mg/dL) and ApoB48 (10.2 ± 6.6mg/dL). ApoC3 levels were elevated (30.2 ± 14.2 mg/dL).

Treatment with volanesorsen reduced mean ApoC3 levels from baseline by 84% after 3 months and by 83% after 6 months (P < 0.001 for both comparisons), corresponding to decreases of 25.7 mg/dL and 25.6 mg/dL, respectively. APOC3 levels increased by 6.1% (1.9 mg/dL) after 3 months and decreased by 5.2% (1.7 mg/dL) after 6 months among patients receiving placebo. The primary efficacy endpoint, ie, the percentage change in triglycerides between baseline and 3 months, was a 77% decrease in the volanesorsen group vs an 18% increase in the placebo group (P < 0.001), corresponding to a decrease of 1,712 mg/dL (95%CI, 1,330 to 2,094) in the volanesorsen group compared with an increase of 92.0 mg/dL (95%CI, -301 to 486 mg/dL) in the placebo group (p < 0.001).

The results of the analysis of the first-ranked secondary endpoint (ie, treatment response rate, defined as a fasting plasma triglyceride level of < 750 mg/dL at 3 months) were significant. Compared with 10% of patients in the placebo group, 77% of patients in the volanesorsen group achieved triglyceride levels < 750 mg/dL (OR, 186.16; 95%CI, 12.86 to could not be estimated; p < 0.001). The results of the second-ranked secondary endpoint (ie, percentage change in fasting triglyceride levels from baseline to 6 months) were also significant: there was a 53% reduction in triglyceride levels in the volanesorsen group (1,380 mg/dL) vs a 25% increase in the placebo group (224 mg/dL). The mean difference between groups was -77.8% (95%CI, -106.4 to -49.1; p < 0.001). The analysis of the third-ranked secondary endpoint (ie, percentage change in fasting triglyceride levels from baseline to 12 months) was significant; volanesorsen reduced triglyceride levels by 40% (986 mg/dL), whereas there was a 9% increase (39 mg/dL) in the placebo group. The between-group difference was -49.1% (95%CI, -94.7 to -3.5; p = 0.03). The subsequent endpoint (ie, the average of maximum intensity of patient-reported abdominal pain during the treatment period in the hierarchical analysis) was not significant.^23^

Among patients in the volanesorsen group, 19 completed the full 52-week treatment period. Six patients received 300 mg per week for the entire treatment period; among the remaining 13 patients, dose frequency was reduced to 300 mg every 2 weeks, treatment was paused, or both. Among patients who did not have a dose reduction, the decrease in triglyceride levels from baseline was 79% at 3 months, 80% at 6 months, and 72% at 12 months (absolute decreases from baseline of 1,670 mg/dL, 1,656 mg/dL, and 1,454 mg/dL, respectively). Absolute decreases in triglyceride levels among the 13 patients whose doses were reduced was 71% at 3 months, 52% at 6 months, and 54% at 12 months (mean decreases from baseline of 1,933 mg/dL, 1,564 mg/dL, and 1,400 mg/dL, respectively). Among the 6 patients whose doses were not reduced, 5 had triglyceride levels < 750 mg/dL at 6 months, and 4 had triglyceride levels < 750 mg/dL at 12 months. Of the 13 patients whose doses were reduced, 6 had triglyceride levels < 750 mg/dL at 6 months, and 6 had triglyceride levels < 750 mg/dL at 12 months; 3 patients achieved triglycerides < 750 mg/dL at 6 and 12 months.^23^ In exploratory analysis, the levels of chylomicron triglycerides, ApoB48, non-HDL-C, and VLDL-C in patients receiving volanesorsen were reduced by 83%, 76%, 46%, and 58%, respectively; in the same patients, the levels of HDL-C, ApoA1, LDL-C, and ApoB were increased by 46%, 14%, 136%, and 20%, respectively.^23^

Volanesorsen reduced triglyceride levels irrespective of patients’ genetic diagnoses or type of mutation. At 3 months, mean triglyceride levels were decreased by 65% in the 17 patients with biallelic mutations in the *LPL* gene and by 75% in the 9 patients with non- *LPL* genetic deficiencies. Patients with mutations in the *APOC2* , *GPIHBP1* , *APOA5* , and *LMF1* genes all showed triglyceride decreases from 69% to 88%. Treatment was also effective irrespective of baseline triglyceride levels and was equally effective in patients receiving concomitant fibrate therapy, ω3 fatty acids, or both and patients not receiving those therapies (mean decrease from baseline to 3 months of 76% and 73%, respectively).^23^

Because of the limited sample size due to the rarity of FCS, a change in the number of AP episodes was not a prespecified endpoint. However, exploratory analysis of adjudicated episodes of AP that occurred during the trial was conducted. During the treatment period, 3 patients in the placebo group had 4 episodes of AP, whereas 1 patient in the volanesorsen group had 1 episode 9 days after receiving the final dose.^23^

The most common adverse events during the treatment period were injection-site reactions and thrombocytopenia. In the volanesorsen group, 20 patients (61%) had at least one mild-to-moderate injection-site reaction and, on average, 12% of volanesorsen injections vs 0% of placebo injections were associated with these reactions. One patient was excluded from the trial due to an injection-site reaction. Confirmed thrombocytopenia < 140,000 per microliter was observed in 25 patients (76%) in the volanesorsen group and in 8 patients (24%) in the placebo group; confirmed thrombocytopenia < 100,000 per microliter was observed in 16 patients (48%) who received volanesorsen but in no patients who received placebo. Because there was no documented history of marked thrombocytopenia in humans treated with this class of antisense drugs,^20^ the initial protocol required platelet count monitoring at intervals of 4 to 6 weeks. However, during the trial, grade 4 thrombocytopenia (< 25,000 platelets per microliter) was observed in 2 patients in the volanesorsen group, and the treatment was discontinued. There were no major bleeding events in any of these patients, and both patients reached normal platelet counts 23 and 33 days after drug discontinuation. One patient received oral prednisone at a dose of 60 mg for 23 days. The other patient received methylprednisolone at a dose of 125 mg for 11 days, followed by oral prednisone at a dose of 70 mg tapered to 50 mg for 21 days, as well as immunoglobulin at a dose of 60 g and 80 g on successive days, followed 4 days later by immunoglobulin at a dose of 40 g daily for 5 more days. Three other patients with lower grades (1 or 2) of thrombocytopenia were withdrawn from the trial by the investigators. After the two cases of thrombocytopenia, a platelet monitoring program consisting of assessments every 2 weeks was established, with a threshold of < 100,000 platelets per microliter for reduction in dose frequency to every 2 weeks, and a new threshold of 75,000 platelets per microliter (changed from 50,000 per microliter) for medication interruption. After these measures were implemented, no patient presented platelet-count declines to < 50,000 per microliter, and no thrombocytopenia-related dose discontinuation occurred. There was a reduction in the frequency of volanesorsen doses in 13 patients, and in 9 patients this was due to thrombocytopenia. Fourteen patients randomly assigned to volanesorsen vs 2 patients randomly assigned to placebo did not complete the 52-week treatment period. Nine discontinued the trial because of adverse events, which included 5 cases of platelet decreases and 4 cases of other volanesorsen-related adverse effects. Four other patients voluntarily withdrew consent. There were no deaths during the study.^23^

The Re-FOCUS^196^ was a retrospective global web-based survey conducted with patients with FCS who received volanesorsen for ≥ 3 months in an open-label extension study. The survey included questions about patients’ experiences before and after treatment with volanesorsen. Twenty-two participants had received volanesorsen for a median of 222 days. Volanesorsen significantly reduced the number of symptoms per patient on the physical, emotional, and cognitive domains. There were significant reductions in episodes of steatorrhea, pancreatic pain, and constant worry about an attack of pain or AP. Respondents also reported that volanesorsen improved overall management of symptoms and reduced interference of FCS with work/school responsibilities. Reductions in the negative impact of FCS on personal, social, and professional life were also reported. Treatment with volanesorsen has the potential to reduce disease burden in patients with FCS through modulation of multiple symptom domains.

Volanesorsen was approved by Anvisa on August 23, 2021, based on data from the Approach and Compass studies. It is indicated for adult patients (> 18 years old) with genetic confirmation of FCS and high risk of pancreatitis.^197^ The drug has been approved by the European Medicines Agency for use in adults with FCS since 2014.

Volanesorsen is not approved by the FDA, although it was investigated in the Approach study in patients with FCS. The disease is considered ultra-rare and debilitating. FCS causes unpredictable and potentially fatal pancreatitis, chronic complications resulting from permanent organ damage, and severe impact on patients’ daily lives. The typical feature of FCS is very high levels of triglycerides. Results from the phase 3 Approach study – the largest study conducted in patients with FCS – showed that, compared with placebo, treatment with volanesorsen reduces triglycerides by 77% (-94% compared with placebo). Medical societies recommend triglyceride reduction as the treatment target for patients with FCS. The most common adverse events are injection-site reactions and reduction in platelet counts.

The FDA’s claim for not approving the drug was safety concerns, especially risk of bleeding due to thrombocytopenia, despite recommendations to mitigate adverse effects. If a possibility for thrombocytopenia was detected during the trial, management with platelet monitoring every 15 days was conducted, which may be more frequent depending on subsequent tests. Likewise, reduction of dose frequency according to platelet count was recommended.

## 13. Social and Psychological Aspects and Economic Impact of the Disease

Variability in early development, differences in symptom severity, and variations in the degree of functional limitations due to physical condition are characteristics of FCS manifestation that interact with other aspects, such as sociodemographic and economic profiles, personality traits, psychosocial and sociocognitive factors, personal skills for coping with adverse health situations, and ability for self-regulation and maintaining a sense of efficacy in the setting of illness.^198^ The link between all these aspects and other contextual aspects makes the management of FCS more complex, which may, in addition to interfering with the adaptive ability of patients and caregivers, demand different medical interventions that are centered on the uniqueness of each patient.

The lack of dissemination of patient statements in the media, caused by the absence of the theme on popular discourse and its limited presence in scientific discourse,^199^ has promoted a pattern of silence surrounding FCS. The lack of familiarity with this condition in the medical community aggravates the biopsychosocial stress experienced by patients, as they have to consult several different specialties in the search for a diagnosis, which usually happens late and does not lead to an effective response to drug therapy. Communication and knowledge gaps challenge patients and caregivers to live with a disease that is often associated with limited empathy, since it lacks socially constructed meaning and a clinically recognized identity.^199^ Therefore, further developing the state of the art of FCS in real life is relevant for reasons beyond the severe deleterious effects of the disease on health and functional capacity.

A study^200^ on the quality of life of patients with FCS demonstrated the validity of self-report instruments in the setting of a rare disease and highlighted the strong negative impact of FCS treatment, which fundamentally consists of restrictive dietary control. Reporting how the disease affects everyday life,^201^ how getting sick affects the perception of satisfaction with quality of life and health status,^202^ and how treatment affects the adaptive capacity of patients^203^ helps to promote awareness of the psychosocial burden of FCS. Topics covered by the self-evaluation of quality of life instrument are listed in Chart 2 .

**Chart 2:** – Topics covered by the self-evaluation of health-related quality of life instrument

Self-evaluation of:
Degree of limitation caused by the condition when performing activities of daily living
Degree of limitation in functional capacity to perform light, moderate, and intense activities of daily living
General health status
Degree of pain interference on daily life
Degree of general health interference (physical and mental) with interpersonal relationships
Degree of vitality in daily life (degree of involvement and motivation with life)
Degree of limitation caused by emotional or cognitive state when performing activities of daily living
Predominant mood at specified time

By providing a standardized and structured instrument to listen to patients with FCS, the psychometric self-report instrument allows to break the pattern of silence, which is characteristic of rare or uncommon diseases.^199^ Although studies of low prevalence diseases include a small number of participants compared with those of chronic and common diseases, they are able to portray the reality of patients and caregivers and point out trends, as well as collaborate in the recommendation of behavioral coping strategies.

### 13.1. Social Aspects in Familial Chylomicronemia Syndrome

Patients diagnosed with a rare disease (also known as an orphan disease) lose, to some extent, their social references and, as they begin to rely more on technical and scientific guidelines to manage their condition, move away from usual health care practices. The lack of information on the history of the disease in real life and the lack of guidelines or position statements for safe and effective medical conduct and guidance impact the personal ability to establish a routine and projects and to maintain interpersonal relationships, as idealized by patients. Lack of knowledge of the disease interferes with the sense of belonging and sustains feelings of helplessness and isolation. Disease invisibility in everyday life reduces the chances of patients and caregivers receiving social support.^198^ A study^204^ showed that strong feelings of misunderstanding may drive patients and caregivers to create adaptive responses that restore familiarity and belonging in religious environments.

Gaps in the medical knowledge of FCS have been shown to hinder communication in clinical practice.^204^ Not understanding the objectives of a therapeutic proposal may lead patients to have unrealistic expectations of treatment scope. The most frequent expectations regarding adherence to treatment among patients with FCS are shown in Figure 1 .^202^

**Figure 1:** – Expectations of patients with FCS regarding adherence to treatment

Having described the physical and psychosocial aspects most severely affected by FCS from a patient perspective, it is worth mentioning that patients’ hopes of restoring a normal lifestyle with treatment can be better managed if professionals are up to date and capable of communicating the diagnosis and the evidence supporting therapeutic recommendations, as well as talk about expected results.^204^

### 13.2. Psychological Aspects in Familial Chylomicronemia Syndrome

Feelings of impotence in the face of the disease, fatigue, and mental confusion are interdisciplinary symptoms that may persist throughout the lives of patients with FCS. Concerns about the impact of the disease on health and life over time, the desire to restore a normal lifestyle, and concerns about the financial impact of the disease greatly affect the emotional stability of patients and caregivers and may produce feelings of low self-esteem and anxiety, interfere with the ability to reason and come up with solutions, and reduce sleep quality.^206^ Depression, feelings of embarrassment, shame, and social inadequacy, and perception of changes in cognitive function due to concentration and memory problems contribute to the decline in the personal and professional quality of life of those affected.^207^ According to patients, living with FCS is time-consuming and drains physical and mental energy, making them unable to plan their lives.^208^ A systematic review^199^ suggests that adults diagnosed with FCS may express significant psychological damage related to the lack of autonomy and freedom in controlling their lives beyond the disease. Those treating or caring for patients with FCS need to be more aware of the psychological aspects associated with the disease.

#### 13.2.1. Parents of children diagnosed with Familial Chylomicronemia Syndrome

FCS manifests in late childhood and adolescence, but some cases have been reported to occur in the first years of life and in neonates.^209^ Rare diseases are challenging not only for patients, but also for family members who care for them. A study^210^ found an increased frequency of parental reports of lack of social support and empathy from health professionals, including complaints about general lack of information and guidance and lack of advice regarding the appropriate way to interact/act with the child. The study showed that fathers tend to be more concerned about the future, whereas mothers are more concerned about the present. The study also revealed that mothers are more likely to report impairments in the quality of social, family, and professional relationships, as they tend to occupy more of their time with basic care and daily routine. Such differences among genders need to be more well known.

## 13.3. Reducing the Impact of the Disease: Ways of Coping

Adherence to general recommendations, usually presented as medical consensus, is essential for health promotion and prevention in primary and secondary health care, as well as for rehabilitation processes. Among health behaviors, therapeutic adherence is one of the most studied self-regulation behaviors, and refers to patients’ active participation in disease management to preserve health and quality of life in the setting of illness.^211^ It should be noted that the proposal of total and permanent therapeutic adherence may generate personal and social conflicts, as well as find resistance on the part of patients or lack of social collaboration, insofar as it can impact interpersonal life projects by interfering with the decision to have children, ability to work, free leisure time, etc.^207^

### 13.3.1. Active and Passive Models for Coping: Focus on the Patient

Aiming at greater success in therapeutic adherence to FCS treatment, patient involvement in decision-making is essential. For this purpose, the use of passive and active coping strategies is recommended. Data from a systematic review^199^ show that passive coping approaches include obtaining/searching for information, clinical advice, genetic counseling, and health education. Examples of active coping approaches in behavioral performance/action in FCS include self-control in restricting fat, alcohol, and carbohydrate consumption; self-regulation in self-medication, avoiding harmful drug interactions; self-administration of drugs for reduction of plasma triglyceride concentrations; and attending follow-up consultations as indicated by the primary care physician.

A study^211^ investigating the effect of Internet use on the adaptive capacity of parents of children with rare diseases showed that gaining knowledge is essential for gradually adapting to the new health reality. The study emphasized that searching for information about the disease can both increase patients’ sense of efficacy and increase anxiety symptoms. As a contemporary reality, the impact of searching for training/information on the Internet by patients and caregivers on the adaptive process of FCS needs to be better understood.

### 13.3.2. Social Model for Coping: Focus on Peers

Obtaining social support through peer groups is known to help improve the perception of general well-being and promote motivation for self-regulation.^212^ The CONNEC study^213^ showed that people affected by FCS may benefit from having contact with other people affected by the disease. The study suggests that participating in support groups, whether by reading texts, joining websites and face-to-face or online conversation circles, interacting with or just learning about other patients, positively affects perception of quality of life and reduces perception of symptom severity and psychological distress, in addition to mitigating psychosocial stress. The implementation of comprehensive measures for coping with and managing the disease such as filling technical and scientific gaps, encouraging patients to engage in therapeutic socialization, and disseminating information about the adverse psychosocial effects of FCS can help promote the construction of a social identity for the disease and establish expertise in health care.^93 , 94^

## 13.4. Cost-effectiveness in the Management of Psychosocial Risks

Knowing that the cost-effectiveness evaluation of health interventions seeks solutions with lower disease-related costs, through which investment allocation can achieve the best results,^214^ it can be assumed that investing in collaborative therapeutic resources for interventions focused on clinical aspects and psychoemotional symptoms^215^ would have a cost-effective return, given that although psychiatric manifestations are not specific to FCS, they hinder therapeutic adherence and lead to recurrent urgencies and hospital admissions.^219^In this sense, it supports data from literature review^216^ showing robust evidence that investing in combined interventions for cardiovascular diseases and anxiety and depression conditions leads to a positive cost-effective result. Finally, the ReFOCUS^196^ study shows that adequate pharmacological treatment can promote symptom control, reduce stress generated by severe dietary restriction, and modify expectations regarding the future. From this perspective, there is no doubt about the close link between FCS and psychosocial aspects and about the potential cost-effectiveness projected in studies and interventions that seek to develop effective drug treatments for patients diagnosed with FCS.^217^

## 14. Summary of Recommendations

**Table:** 

	Class of recommendation	Level of evidence
Blood collection for triglyceride measurements in adults should be performed after a 12-hour fast. Patients should maintain their usual diet but should not consume alcohol (72 hours) or engage in physical exercise (24 hours). In children, fasting duration varies according to the age group. In infants up to 1 year, blood should be collected after a 3-hour fast or immediately before the next feeding. In noninfants from 2 to 5 years, blood should be collected after a 6-hour fast. Children over 5 years old and adolescents should fast for 12 hours	I	C
To confirm a suspected case of FCS after excluding secondary causes of HTG, triglyceride levels should be: 1) > 1,000 mg/dL, in 3 different measurements, for adults after a 12-hour fasting; 2) > 880 mg/dL, in 3 different measurements, for children and adolescents irrespective of fasting time; 3) in children or adults, a triglyceride level < 170 mg/dL **excludes** the investigation of hyperchylomicronemia	I	C
Triglycerides > 1,000 mg/dL increase the risk of pancreatitis in patients with FCS	IIa	C
Levels of LDL-C in FCS may be underestimated irrespective of measurement method. However, if measured, Martin’s formula or, preferably, direct LDL testing should be performed	I	C
The FCS diagnostic score is a useful tool for suspected FCS and is recommended as a screening tool for genetic testing	I	C
This document does not recommend measuring LPL activity with heparin, as it may have limited discriminative capacity in carriers of common variants	III	C
Genetic sequencing of the *LPL* , *APOC2* , *APOA5* , *GPIHBP1* , and *LMF1* genes provides a definitive diagnosis of FCS in case of homozygosis or double/compound heterozygosis for pathogenic or probably pathogenic variants	I	C
For confirmed cases of FCS, genetic counseling should be conducted to calculate the risk of condition occurrence or recurrence, both for decision-making and for choosing the contraceptive method, especially in consanguineous unions	I	C
Nutritional therapy should include the following general recommendations: Restriction of fat consumption (10% to 15% of TEI) Exclusion of added sugars (sucrose and corn syrup) Exclusion of concentrated fruit juices Exclusion of alcoholic beverages Consumption of complex carbohydrates in adequate amounts Ensuring the adequacy of essential fatty acids Monitoring consumption of fat-soluble vitamins, with supplementation when necessary Inclusion of MCT for the purpose of calorie intake, according to tolerance	I	C
Intravenous heparin infusion for HP is not recommended in patients with FCS	III	C
The use of low-molecular-weight heparin is indicated as prophylaxis for deep venous thrombosis in HP in patients with FCS	IIa	C
In patients with FCS and HP, intravenous insulin should only be used in those with decompensated type 1 and 2 diabetes, for glycemic control	IIa	C
Plasmapheresis should be indicated for patients with FCS and HP on an individual basis. Potential candidates include patients with severe HP or who persist with triglycerides > 1,000 mg/dL after the first 24 to 48 hours	IIb	C
The indication of plasmapheresis during pregnancy, although safe and effective, should be individualized due to the scarcity of evidence to date	IIb	C
The use of antisense treatment against ApoC3 is recommended for adults aged > 18 years with genetic confirmation of FCS who did not respond to usual treatment and have high risk of pancreatitis	I	C
Platelet monitoring during antisense treatment against ApoC3 should be done initially every 2 weeks and subsequently adjusted according to platelet count	I	B
Antisense treatment against ApoC3 should be spaced if platelets < 100,000/uL, and the drug should be discontinued if platelet count < 75,000/uL	I	B
